# Linking Skin and Joint Inflammation in Psoriatic Arthritis through Shared CD8^+^ T Cell Clones

**DOI:** 10.1002/art.43286

**Published:** 2025-09-21

**Authors:** Lucy E. Durham, Frances Humby, Nora Ng, Roman Laddach, Elizabeth H. Gray, Sarah E. Ryan, Kathryn J. A. Steel, Rosie Ross, Giovanni A. M. Povoleri, Rosamond Nuamah, Kathy Fung, Athul Menon Kallayil, Pawan Dhami, Bruce W. Kirkham, Leonie S. Taams

**Affiliations:** ^1^ Centre for Inflammation Biology and Cancer Immunology, Department of Inflammation Biology School of Immunology and Microbial Sciences, King's College London London United Kingdom; ^2^ Department of Rheumatology Guy's and St Thomas’ NHS Foundation Trust London United Kingdom; ^3^ Spatial Biology Facility, Guy's Cancer Centre, Faculty of Life Sciences and Medicine and Faculty of Dentistry, Oral and Craniofacial Sciences King's College London London United Kingdom; ^4^ Spatial Biology Facility, Guy's Cancer Centre, Faculty of Life Sciences and Medicine and Faculty of Dentistry, Oral and Craniofacial Sciences King's College London and Genomics Facility, Guy's and St Thomas’ NHS Foundation Trust London United Kingdom; ^5^ Genomics Facility Guy's and St Thomas’ NHS Foundation Trust London United Kingdom

## Abstract

**Objective:**

Psoriatic arthritis (PsA) is an HLA class I–associated inflammatory arthritis that develops in up to 30% of people with psoriasis. We tested the hypothesis that skin and joint inflammation in PsA is linked in terms of CD8^+^ T cell phenotype and clonality.

**Methods:**

Using single‐cell RNA sequencing (n = 6 skin samples with n = 5 paired synovial tissue samples and/or n = 5 paired synovial fluid samples) and spatial transcriptomics (n = 1 paired skin and synovial biopsy sample, n = 4 unpaired biopsy samples), we compared the transcriptional signature, T cell receptor repertoire, and cell neighborhoods of T cells from skin and synovial tissue and/or fluid samples from patients with PsA.

**Results:**

We identified an enrichment of type 17 CD8^+^ tissue‐resident memory T (Trm) cells in both the skin and joint, with a stronger interleukin‐17 signature in the skin than the joint. CD8^+^ Trm cells resided in distinct cell neighborhoods in the skin and joint but were located adjacent to antigen‐presenting cells in both sites. Several T cell clones were shared between the skin and joint. Across the six patients, 155 CD8^+^ T cell clones were shared between the two sites, comprising 1,071 CD8^+^ T cells and taking up a median of 13% of the skin and 8% of the joint CD8^+^ T cell receptor repertoire. CD8^+^ skin–joint shared clones tended to have a similar phenotype at both sites, characterized by increased expression of genes associated with a cytotoxic, tissue‐resident phenotype.

**Conclusion:**

Our findings support the hypothesis that skin and joint inflammation in PsA is linked in terms of CD8^+^ T cell clonality and that specific T cells migrate between these compartments to propagate inflammation across both sites.

## INTRODUCTION

Psoriatic arthritis (PsA) is an inflammatory disease that affects both skin and joints and develops in up to 30% of people with the skin condition psoriasis.^1^ It is unclear what initiates chronic inflammation in PsA and whether the immune mechanisms underlying skin and joint inflammation are the same. We hypothesized that skin and joint inflammation in PsA is linked in terms of CD8^+^ T cell gene signature and clonality.

Evidence indicates a role for both interleukin‐17 (IL‐17) and antigen‐driven activation of CD8^+^ T cells in psoriasis and PsA. Type 17 tissue‐resident CD8+ T cells are enriched in the skin in psoriasis and in the joints in PsA, pointing to a key role for these cells in driving inflammation in both sites.^2^, ^3^, ^4^, ^5^, ^6^, ^7^, ^8^ Monoclonal antibodies that target IL‐17A and IL‐17F treat both psoriasis and PsA, confirming the clinical relevance of the IL‐17 pathway in these diseases.^9^, ^10^ The genetics of both psoriasis and PsA furthermore supports a role for the IL‐17 pathway as well as for major histocompatibility complex (MHC) class I–mediated presentation of antigens to CD8^+^ T cells.^11^, ^12^, ^13^, ^14^, ^15^

The T cell receptor (TCR) repertoire in inflamed joints is distinct from that in blood, suggesting antigen‐driven recruitment and/or activation of synovial T cells in PsA.^16^ Identical TCRβ complementarity‐determining region 3 (CDR3) sequences have been identified in paired skin and synovium samples from the same patients.^17^, ^18^ In both psoriasis and PsA, effective treatment of inflammation is associated with dispersion of the polyclonal T cell infiltrate, leaving a comparatively oligoclonal population, which in the skin has been shown to express IL‐17.^19^, ^20^ This resident population of clonally expanded IL‐17^+^ T cells is thought to be a critical driver of disease recurrence upon cessation of treatment.^3^, ^20^, ^21^ Previous studies also suggest that CD8^+^ T cells recirculate between inflamed tissue and blood in patients with psoriatic disease: CD8^+^ T cells that express skin‐homing receptors are increased in the peripheral blood (PB) of patients with psoriasis and PsA at levels that correlate with disease activity and markers of systemic inflammation.^22^, ^23^ Furthermore, circulating CCR4^+^CD8^+^ T cells in blood share TCRs with clonally expanded CD8^+^ Teff cells in the joints of patients with PsA, raising the possibility of circulation of T cells between the skin and joint.^24^

Despite compelling evidence for a link between psoriasis and PsA, differences have been noted, including different HLA class I gene associations and risk alleles identified by genome‐wide association studies.^11^, ^25^, ^26^ Clinically, the two diseases can have different therapy responses, particularly to ciclosporin and sulfasalazine.^27^, ^28^ Lastly, in rare studies in which paired skin and synovial tissue (ST) cellular phenotypes were directly compared, gene expression profiling revealed a stronger IL‐17/IL‐23 signature in the skin compared to tumor necrosis factor α (TNFα), IL‐6, and interferon‐γ (IFNγ) signatures in ST.^29^, ^30^ The limitation of the few studies to date that have directly compared skin and synovium in PsA is that they have compared either the TCR sequence or the phenotype of bulk cells. To address this knowledge gap, we used single‐cell RNA sequencing (scRNAseq) and spatial transcriptomics to directly compare the gene signature, TCR repertoire, and cell neighborhoods of T cells from paired skin and ST samples from patients with PsA.

## METHODS

### Patients and sample processing

Six patients with active PsA donated PB samples with paired samples of inflamed skin (n = 6), ST (n = 5), and/or synovial fluid (SF; n = 5) from an inflamed knee joint for scRNAseq analysis; four of these patients also donated skin (n = 2) and/or ST (n = 3) samples for spatial transcriptomics (Supplementary Table S1). A seventh patient donated an ST sample for spatial transcriptomics only (Supplementary Table S1). Most patients had longstanding PsA and had developed skin psoriasis before arthritis, three were not receiving treatment, one took low‐dose prednisolone, and one each was taking methotrexate, bimekizumab, and adalimumab (Supplementary Table S1). PB and SF mononuclear cells were isolated using Lymphoprep. Skin punch biopsy samples were incubated at 37°C in 10 mg/mL of Dispase II for one hour to allow separation of the epidermis. The epidermis and ST were then dissociated in 0.3 mg/mL of Liberase and 0.1 mg/mL of DNase. Samples were stained with fluorescently labeled, TotalSeqC and hashtag antibodies (Supplementary Tables S2 and S3). Memory T cells (CD45RA^−^CD27^+^, CD45RA^−^CD27^−^, and CD45RA^+^CD27^−^) were sorted, and cells from all tissues were pooled and retained on ice pending library preparation (Supplementary Figure S1). Biopsy samples for spatial transcriptomics were fixed in formalin and embedded in paraffin. Detailed methods and reagents are included in the Supplementary Methods. All participants gave voluntary informed consent to participate in the trial, and ethical approval was granted by the London ‐ Harrow Research Ethics Committee (ref 17/LO/1940) and the National Research Ethics Service Committee London ‐ Bromley (ref 07/H0809/35).

### scRNAseq and spatial transcriptomics

5′ Gene expression, TCR, and feature barcoding libraries were prepared using 10x Genomics Chromium Single‐Cell 5′ Reagent Kits v1.1 and were sequenced using NextSeq2000. Data were normalized with regression of mitochondrial gene percentages, the cell cycle score, and the digestion module score (to correct for changes in gene expression arising from tissue dissociation^31^, ^32^), then integrated and analyzed using Seurat (Supplementary Figures S2 and S3). The scRNAseq data are available in the GEO database^33^: GSE250242 (PsA patients 1–4) and GSE250243 (PsA patients 5 and 6). An independent analysis of blood, SF, and ST T cells from PsA patients 1, 2, 3, and 4 in combination with two additional patients has been published separately.^32^

The spatial transcriptomics analysis of fixed tissue sections was performed using the CosMx platform using the 1,000‐plex panel. Data were analyzed using AtoMx, CosMx‐lite, Seurat, and CatsCradle. The scRNAseq and spatial transcriptomics data analyses are described in detail in the Supplementary Methods.

## RESULTS

### Enrichment of CD8 ^+^ tissue‐resident memory T cells in the skin and joint compared to blood

**Figure 1:**
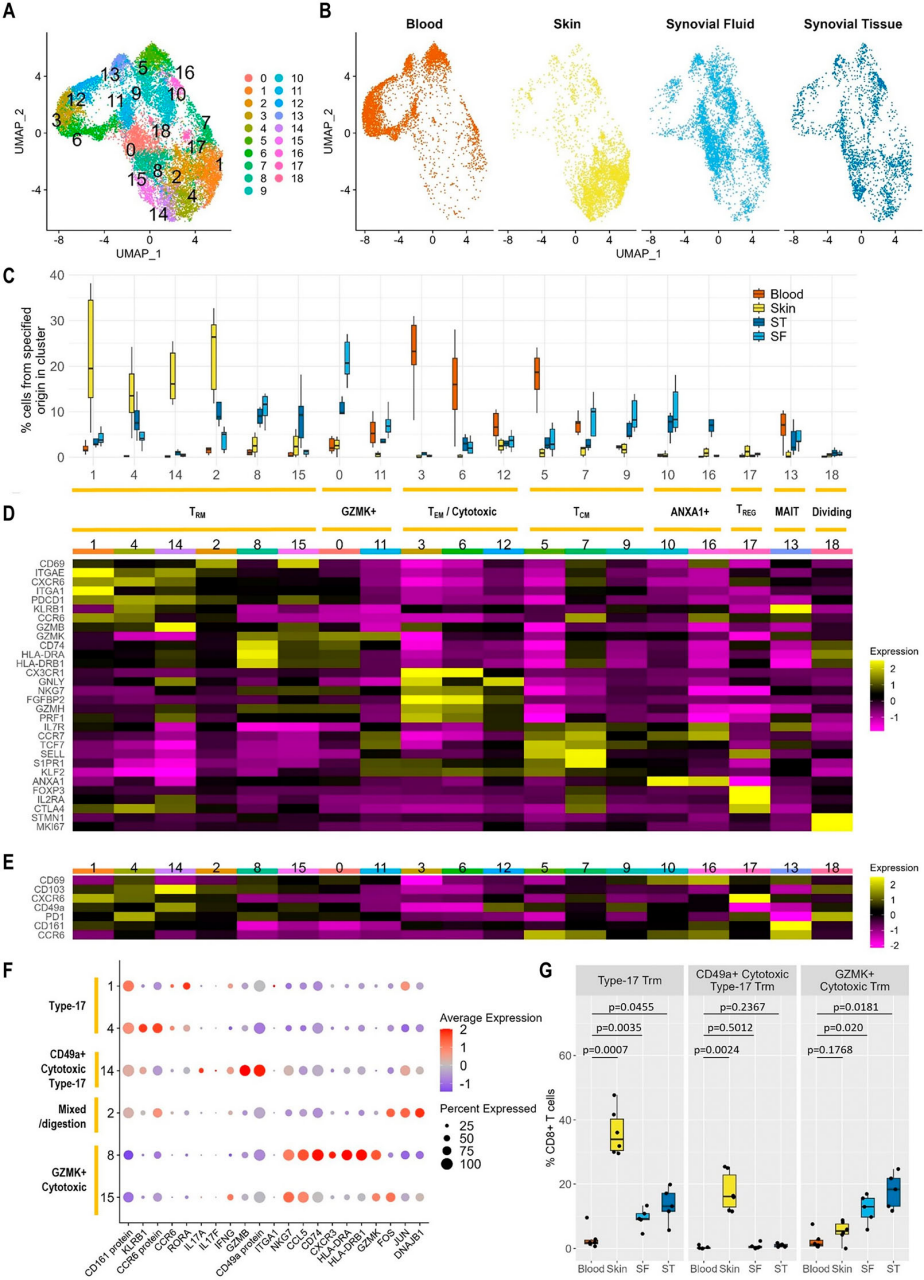
Signature of memory CD8^+^ T cells in PsA. (A) UMAP of integrated analysis of 15,335 memory CD8^+^ T cells from blood, the lesional skin epidermis, and ST and/or SF from an inflamed joint (n = 6 patients). Each dot represents a cell, colored according to the 19 clusters obtained after Seurat clustering. The proximity of the dots to each other indicates similarity of the cells. (B) UMAPs split by tissue of origin. (C) Distribution of CD8^+^ T cells from each tissue across the 19 clusters (n = 6 patients). Clusters are ordered in groups with similar phenotypes. (D and E) Heat maps visualizing averaged expression of specified genes (D) and proteins (E) in each cluster. (F) Dot plot depicting expression of specified genes across CD8^+^ Trm clusters. The size of the dots indicates the percentage of cells within the cluster that express the indicated gene. Color indicates the scaled expression of the indicated gene. (G) Frequency of total memory CD8^+^ T cells from blood, skin, SF, and ST within type 17 (clusters 1 and 4 combined), CD49a^+^ cytotoxic type 17 (cluster 14), and GZMK^+^ cytotoxic (clusters 8 and 15) Trm subsets. Boxplots show the median ± interquartile range. Mixed‐effects analysis comparing skin (n = 6) to blood (n = 6), SF (n = 5) to blood (n = 6), and ST (n = 5) to blood (n = 6) with Dunnett's correction for multiple comparisons. MAIT, mucosal‐associated invariant T; PsA, psoriatic arthritis; SF, synovial fluid; ST, synovial tissue; Tcm, central memory T; Tem, effector memory T; Trm, tissue‐resident memory T; UMAP, uniform manifold approximation and projection.

Seurat clustering of 15,335 memory CD8^+^ T cells from blood, skin epidermis, ST, and/or SF samples from six patients yielded 19 clusters (Figure 1A, Supplementary Data S2). Whereas skin and blood CD8^+^ T cells clustered distinctly from each other on the uniform manifold approximation and projection (UMAP), ST and SF T cells had overlapping clustering profiles (indicating similarity^32^) and were spread more diffusely across the whole UMAP, indicating that some synovial cells had a gene signature similar to cells from blood, some had a gene signature similar to cells from skin, and others had a gene signature more specific to the joint (Figure 1B and C).

Clusters were annotated by examination of differentially expressed genes and gene set enrichment analysis (GSEA) (Figure 1C–E, Supplementary Figure S4, Supplementary Data 2). The major CD8^+^ T cell subsets known to be present in inflamed tissues in psoriasis and PsA were identified, including tissue‐resident memory T (Trm) cells (clusters 1, 2, 4, 8, 14, and 15), granzyme K^+^ (GZMK^+^; clusters 0 and 11), cytotoxic T cells (clusters 3, 6, and 12), central memory T cells (clusters 5, 7, and 9), annexin A1^+^ (*ANXA1* ^+^; clusters 10 and 16), Treg‐like cells (cluster 17), mucosal‐associated invariant T cells (cluster 13, with significantly up‐regulated expression of *TRAV1/2* and *KLRB1*; Supplementary Data 2), and dividing (cluster 18) cells.^4^, ^7^, ^8^, ^16^, ^34^

Because Trm cells are enriched at sites of inflammation in both psoriasis and PsA^3^, ^4^, ^6^, ^7^, ^8^ and are hypothesized to contribute to the persistence of both diseases,^3^, ^7^, ^20^, ^35^ these cells were investigated in more detail. To identify Trm cell clusters, we used differential gene expression in combination with GSEA.^32^ Under homeostatic conditions, human Trm cells are frequently identified as CD69^+^CD103^+/−^.^36^ However, in our data set, CD69 RNA/protein was diffusely expressed across most clusters that contained skin and synovial cells and likely reflected T cell activation rather than residency (Supplementary Figure S5). Therefore, we identified clusters that might represent Trm clusters (“potential Trm” clusters, clusters 0, 1, 2, 4, 8, 14, and 15) as clusters with significant differential expression of three or more genes/proteins associated with the core transcriptional signature of human Trm cells (increased *ITGAE*/CD103, *CD69*/CD69, *CXCR6*/CXCR6, *ITGA1*/CD49a, *PDCD1*/PD‐1; decreased *S1PR1*, *SELL*, *KLF2*, with at least one gene/protein increased and one decreased)^36^ (Supplementary Figure S6A, Supplementary Data 2). GSEA was then performed for each potential Trm cluster compared to pooled cells from nonpotential Trm clusters. Clusters that were positively enriched for a core Trm signature from homeostatic lungs^36^ (clusters 1, 2, 4, 8, 14, and 15) were then classified as Trm clusters (Supplementary Figure S6B).

Examination of gene expression (Figure 1F, Supplementary Data 2) and GSEA comparing each Trm cluster to pooled cells from the other Trm clusters (Supplementary Figure S6C) identified three key CD8^+^ Trm cell subsets: type 17 Trm cells (clusters 1 and 4), which were significantly enriched in the skin and also in joints (as we have previously shown^6^, ^32^) compared to blood; CD49a^+^ cytotoxic type 17 Trm cells (cluster 14), which were significantly enriched in skin compared to blood; and GZMK^+^ cytotoxic Trm cells (clusters 8 and 15), which were significantly enriched in joints compared to blood (Figure 1G). Trm cell cluster 2 was not enriched for either a type 17 or a CD49a^+^GZMK^+^ Trm signature but was positively enriched for a signature associated with tissue that has undergone collagenase digestion^31^, ^32^ and was therefore classified as a “mixed/digestion” signature subset (Figure 1F).

### Differential gene signatures between skin and joint CD8 ^+^ T cells

**Figure 2:**
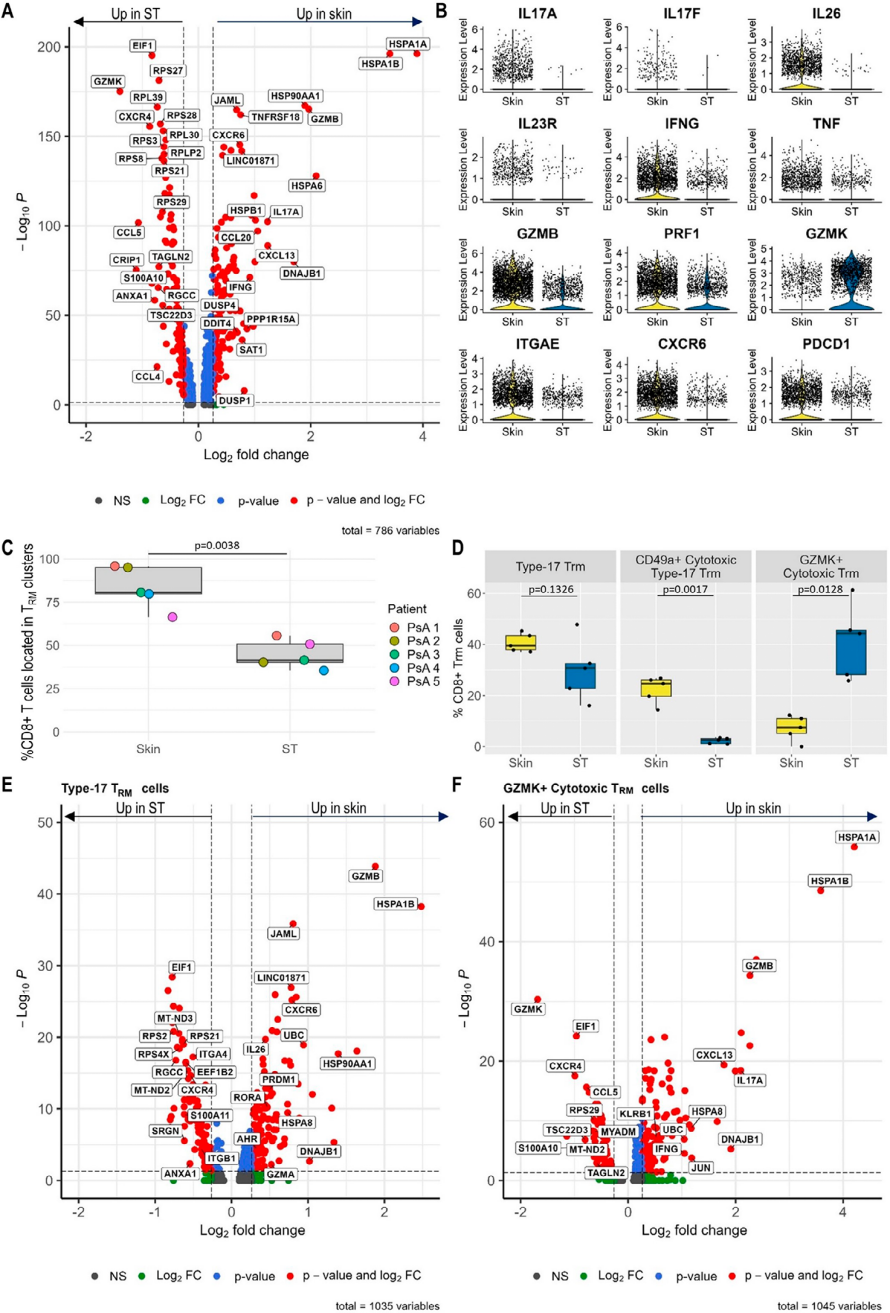
Comparison of skin and ST CD8^+^ T cells in PsA. (A) Volcano plot depicting differentially expressed genes between memory CD8^+^ T cells from paired skin and ST samples (n = 5). Differential expression of pooled cells from all patients was calculated with the Wilcoxon signed rank test using FindMarkers() applied to SCTransformed RNA in Seurat. To mitigate potential batch effect/variability, only genes that were also significantly differentially expressed by FindConservedMarkers() (which performs differential gene expression testing for each patient then combines *P* values using meta‐analysis methods from the MetaDE package) or significantly differentially expressed in three or more of five patients after applying FindAllMarkers to each patient sample individually are labeled. The top 20 increased and decreased genes meeting these criteria are labeled. (B) Violin plots depicting expression of specified genes in skin (n = 6) and ST (n = 5) samples. (C) The percentage of CD8^+^ memory T cells from paired skin and ST (n = 5) that are Trm cells. (D) The percentage of CD8^+^ Trm cells from paired skin and ST (n = 5) belonging to each Trm subset. (E and F) Volcano plots visualizing differentially expressed genes between paired skin and ST within type 17 (E) and GZMK^+^ cytotoxic (F) Trm cells (n = 5). The top 10 increased and decreased genes and genes that are mentioned in the text are labeled. Calculation of differential genes and the criteria for labeling were as described for panel A. Boxplots show the median ± interquartile range (paired *t*‐tests; two‐tailed, n = 5). NS, not significant; PsA, psoriatic arthritis; ST, synovial tissue; Trm, tissue‐resident memory T. Color figure can be viewed in the online issue, which is available at http://onlinelibrary.wiley.com/doi/10.1002/art.43286/abstract.

Compared to joint CD8^+^ T cells, epidermal CD8^+^ T cells had significantly increased expression of *CXCR6*, *IFNG*, and *GZMB*; a stronger IL‐17 signature with significantly increased expression of *IL17A* and *CCL20* compared to ST CD8^+^ T cells (Figure 2A, Supplementary Data 3); and increased expression of other type 17 genes, including *IL17F*, *IL26*, and *IL23R* (Figure 2B). In contrast, ST CD8^+^ T cells had significantly increased expression of *GZMK*, *CXCR4*, *CCL4*, *CCL5*, *ANXA1*, and several ribosomal genes. There were also differences in the frequency of CD8^+^ Trm cells between skin and ST: a median of 81% of skin CD8^+^ T cells resided in Trm cell clusters, compared to 41% of ST CD8^+^ T cells (Figure 2C). Within CD8^+^ Trm cells, the frequency of type 17 Trm cells was not significantly different between skin and ST. However, CD49a^+^ cytotoxic type 17 Trm cells were significantly increased in skin compared to ST (where they were virtually absent), whereas GZMK^+^ cytotoxic Trm cells were significantly increased in ST (Figure 2D).

There were also differences between skin and ST within individual Trm subsets. Epidermal type 17 CD8^+^ Trm cells had gene expression consistent with a stronger type 17 (*IL26* ^37^, *RORA* ^37^), cytotoxic (*GZMB*, *GZMA*), and tissue‐resident (*CXCR6* ^36^, *PRDM1* ^38^, *AHR* ^39^) phenotype than ST type 17 CD8^+^ Trm cells (Figure 2E). In contrast, ST type 17 Trm cells expressed higher levels of the chemokine receptor *CXCR4* and integrins, including *ITGB1* and *ITGA4*, encoding the α4 subunit of the gut‐homing receptor α4β7. Moreover, although less frequent in number, compared to those in the ST, skin CD49a^+^GZMK^+^CD8^+^ Trm cells showed significantly increased expression of *GZMB*, potentially indicating greater cytotoxicity, and higher *IL17A* expression, suggesting a coexisting type 17 phenotype, whereas those from ST showed higher expression of *GZMK* (Figure 2F).

These data show that skin CD8^+^ T cells have a stronger IL‐17 signature and higher frequency of Trm cells compared to joint CD8^+^ T cells. Within Trm cell clusters, the frequency of type 17 Trm cells was similar in skin and ST, but the majority of cytotoxic Trm cells from the skin also had a type 17 signature, whereas those from the joint coexpressed *GZMK* instead. GZMK and IL‐17A were also detected at the protein level in the cell‐free SF of patients with PsA, suggesting that the observed gene expression translates to protein secretion in the inflamed joint (Supplementary Figure S7).

### CD8 ^+^ Trm cells interact with antigen‐presenting cells in the skin and joint

**Figure 3:**
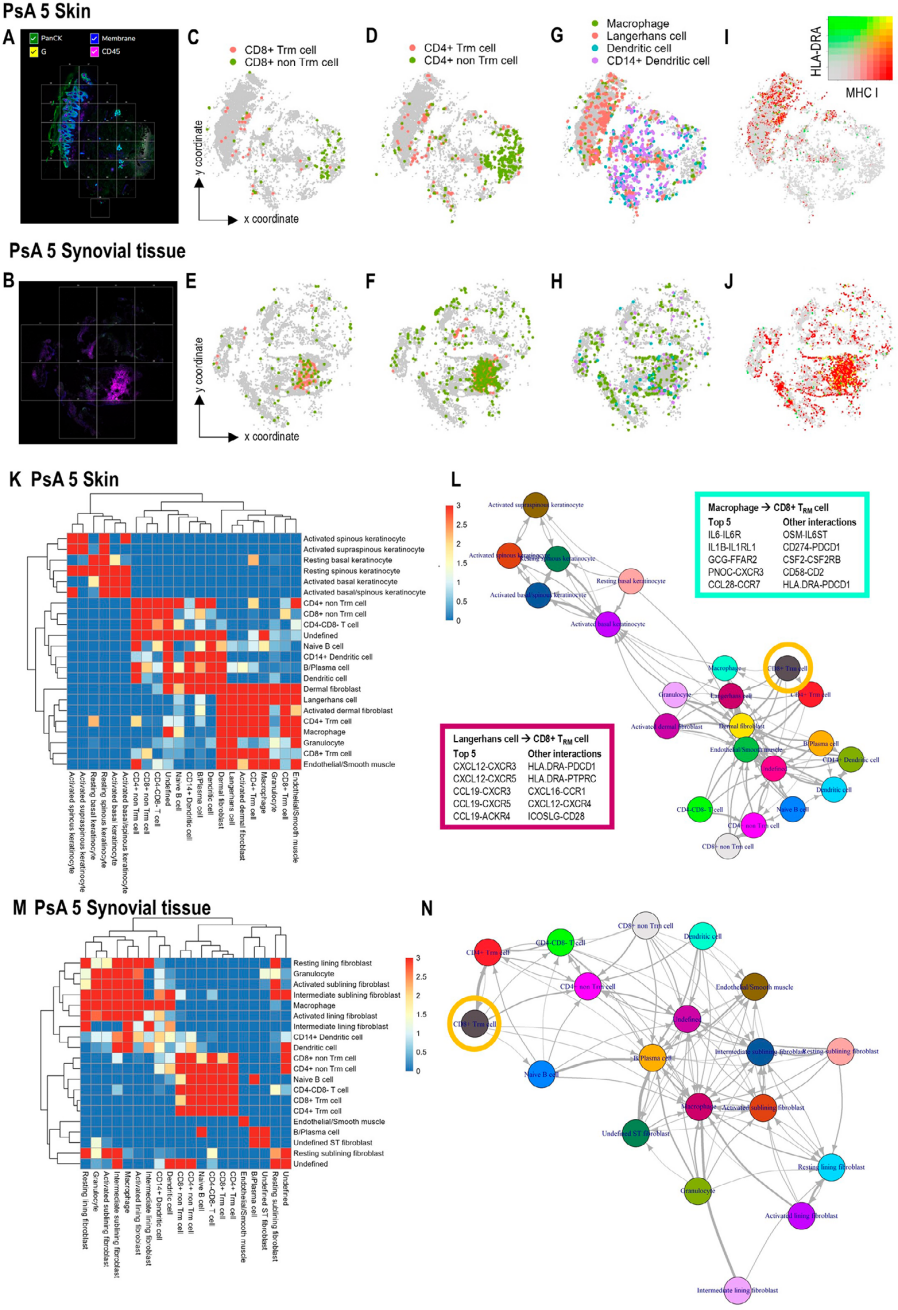
Neighborhood analysis in inflamed skin and joints in PsA. (A–J) Tissue sections from paired skin (A, C, D, G, and I) and ST (B, E, F, H, and J) from PsA patient 5. (A and B) Tissue sections imaged on the CosMx analyzer. Grid lines indicate 0.51 × 0.51 mm fields of view selected for data acquisition. Location of CD8^+^ (C and E) and CD4^+^ (D and F) Trm and non‐Trm cells, myeloid cell subsets (G and H), and MHC class I expression (red), HLA–DR expression (green), and coexpression (yellow) (I and J). (K and M) Heat maps depicting significance of contact‐based interactions between cell types in paired skin (K) and ST (M). The color of the square indicates the −Log_10_ *P* value (upper tail, one‐sided) for cell types that are more frequently neighbors than expected by chance. (L and N) Force‐directed graphs depicting contact‐based interactions between cell types in paired skin (L) and ST (N). Arrows between cell types indicate that the neighborhoods of the cell type the arrow originates from comprise ≥5% of the cell type that the arrow points to. Yellow circles highlight nodes corresponding to CD8^+^ Trm cells. Selected significantly enriched ligand–receptor interactions between macrophages and CD8^+^ Trm cells and Langerhans cells and CD8^+^ Trm cells are listed in boxes. MHC, major histocompatibility complex; PsA, psoriatic arthritis; ST, synovial tissue; Trm, tissue‐resident memory T. Color figure can be viewed in the online issue, which is available at http://onlinelibrary.wiley.com/doi/10.1002/art.43286/abstract.

We sought to use spatial transcriptomics to characterize the location and cell neighborhoods of CD8^+^ Trm cells in paired and unpaired skin and ST samples. This analysis used biopsy samples from five patients comprising six biopsy samples in total: two skin biopsy samples and four ST biopsy samples (one patient donated paired skin and ST samples for spatial transcriptomics; Figure 3A and B, Supplementary Table S1). First, each section was analyzed independently, and canonical gene expression was used to identify broad cell types, including immune cells, fibroblasts, endothelial/smooth muscle cells, and keratinocytes (Supplementary Figures S8 and S9). Then cells from all six sections that belonged to the same broad cell type were integrated and clustered to allow identification of specific cell subsets based on differentially expressed genes between clusters (Supplementary Figures S10 and S11). Cell annotations corresponded with the tissue architecture of sequential hematoxylin and eosin (H&E)–stained sections, for example, keratinocytes were located in the epidermis, and the location of immune cells identified in the spatial transcriptomics analysis corresponded with areas of dense cell infiltration in H&E‐stained tissue (Supplementary Figure S12). A cluster of CD8^+^ T cells with a Trm signature (cluster 1) was identified, but we did not have the granularity to identify individual CD8^+^ Trm cell subsets (Supplementary Figure S10G).

As expected, CD8^+^ and CD4^+^ Trm cells in skin were located primarily in the epidermis, whereas non‐Trm cells were predominantly in the dermis (Figure 3C and D, Supplementary Figure S13).^3^, ^4^ In contrast, Trm and non‐Trm cells tended to colocalize in the joint (Figure 3E and F, Supplementary Figure S13). Similarly, myeloid cells were spatially segregated in the skin and not in the joint (Figure 3G and H, Supplementary Figure S13). In both the skin and joint, CD8^+^ Trm cells tended to cluster in areas that contained cells that coexpressed HLA class I and II (including Langerhans cells and macrophages in the skin and B cells in joints), suggesting antigen presentation to CD8^+^ Trm cells in both sites (Figure 3I and J, Supplementary Figure S13). This is consistent with previous work with skin biopsy samples in psoriasis that reports colocation of T cells and dendritic cells in psoriasis and the role of these interactions in polarizing T cells toward a type 17 phenotype.^40^, ^41^, ^42^ In contrast, in both the skin and the joint, CD8^+^ non‐Trm cells were not restricted to areas containing cells that coexpressed HLA class I and II. Analysis of cell neighborhoods and cell–cell interactions revealed that CD8^+^ Trm cells in skin were located adjacent to antigen‐presenting cells, including Langerhans cells and macrophages (Figure 3K and L, Supplementary Figures S14 and S15). Ligand–receptor interaction analysis in the skin suggested a role for Langerhans cells and macrophages in both recruitment of CD8^+^ Trm cells to the epidermis through several chemokine receptor interactions and costimulation of CD8^+^ Trm cells (eg, ICOSLG‐CD28^43^ and CD58‐CD2 interactions for Langerhans cells and macrophages, respectively, with CD8^+^ Trm cells) (Figure 3L, Supplementary Figure S16). Macrophages also exhibited the potential to drive type 17 differentiation of CD8^+^ Trm cells through IL‐6 and IL‐1β signaling.^37^ In contrast, CD8^+^ Trm cells in the joint were predominantly located in neighborhoods with other lymphocytes, including non‐Trm cells, CD4^+^ T cells, and B cells (Figure 3M and N, Supplementary Figures S14 and S15).

### CD8 ^+^ T cell clones are shared between the skin and joint

Given the evidence for colocalization of CD8^+^ Trm cells with antigen‐presenting cells, we investigated for clonal sharing between the skin and joint. A clone was defined as a group of T cells expressing identical TCRα and β chains. Skin–joint shared clones were defined as T cells from the same clone present in the skin and joint. We recently showed that SF and ST T cells show considerable similarities in terms of TCR repertoire and signature^32^; therefore, SF and ST T cells were pooled to increase the power to detect clones shared between the skin and joint.

**Figure 4:**
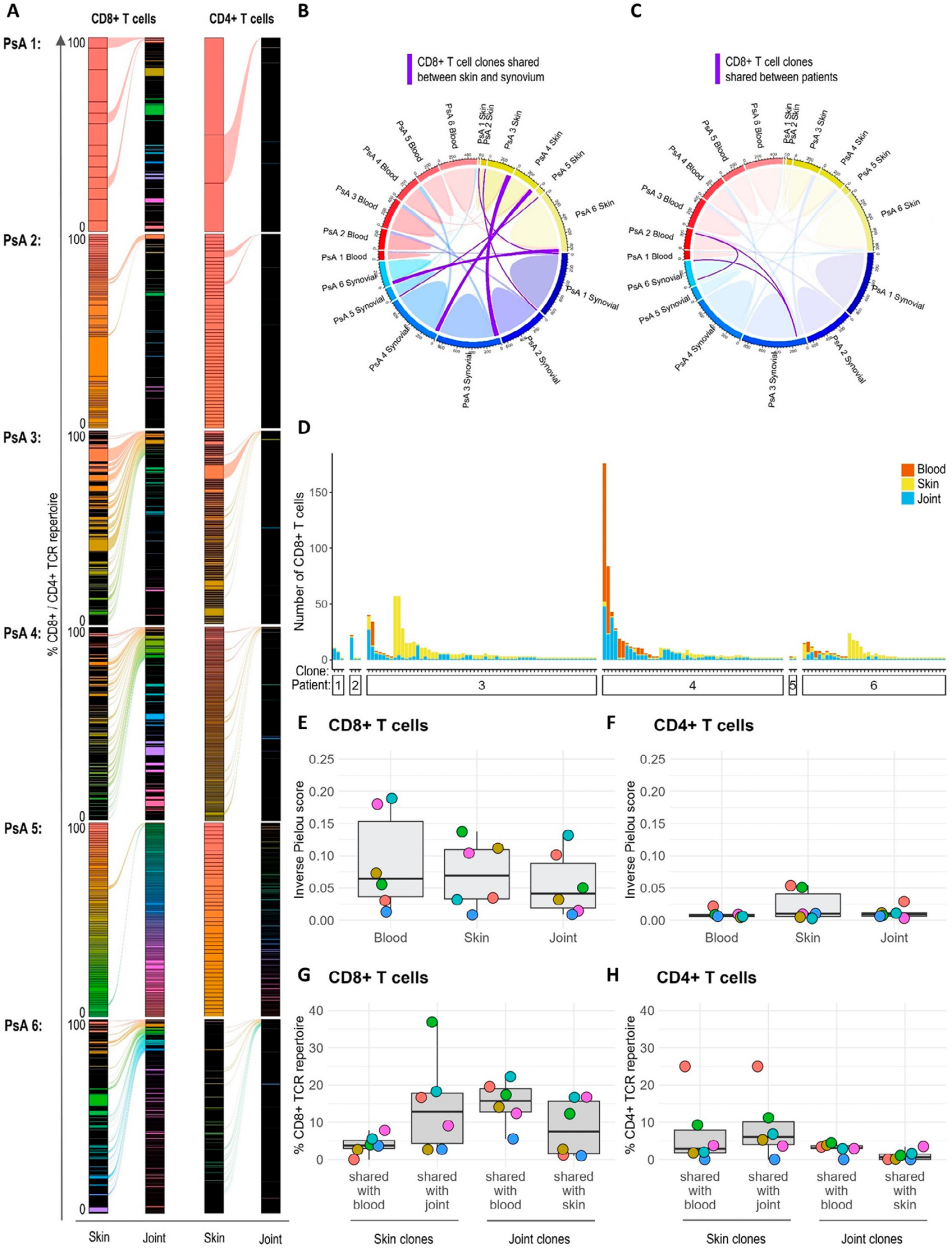
T cell clones are shared between the skin and joint. (A) Alluvial plots visualizing CD8^+^ and CD4^+^ TCR repertoires in the skin and joint (SF and ST combined) (n = 6). Each block represents a T cell clone (single/group of T cells with same TCR). The height of the block represents the percentage of skin/joint CD8^+^ TCR repertoires taken up by clones. Alluvials represent the skin–joint shared clones. (B and C) Circle plots visualizing sharing of CD8^+^ T cell clones between the skin (yellow bar), joint (blue), and blood (red) for each patient. The size of the circumferential bar is proportional to the number of TCRs. (B) Skin–joint, skin–blood, and joint–blood shared clones are represented as purple, red, and blue lines, respectively. (C) Clones shared between patients are represented as purple lines. (D) Bar chart visualizing the size (number of cells) and tissue location of the 155 skin–joint shared clones. Each bar represents a skin–joint shared clone. The height of the bar represents the number of CD8^+^ T cells within that clone, and the color represents the tissue location of cells. Clones are ordered by patient then size. (E and F) Inverse Pielou scores (range: 0, indicating polyclonal, to 1, indicating monoclonal population) for blood, skin, and joint CD8^+^ (E) and CD4^+^ (F) T cells. (G and H) The percentage of skin/joint CD8^+^ (G) and CD4^+^ (H) TCR repertoires taken up by shared clones. PsA, psoriatic arthritis; SF, synovial fluid; ST, synovial tissue; TCR, T cell receptor Color figure can be viewed in the online issue, which is available at http://onlinelibrary.wiley.com/doi/10.1002/art.43286/abstract.

CD8^+^ skin–joint shared T cell clones were detected in all patients, with a total of 155 CD8^+^ T cell clones shared between the skin and joints across patients, comprising 1,071 CD8^+^ T cells (Figure 4A and B). Some CD8^+^ T cell clones were also shared between PsA patients 2, 3, and 6 (Figure 4C). HLA genotyping was not available for PsA patient 2, but PsA patients 3 and 6 were both HLA–A*02:01 ^+^; therefore, it is possible that clones shared between these patients may recognize antigens presented by HLA–A*02:01. Of note, the clones shared between patients were different from the clones shared between the skin and joints within patients.

Of the 155 skin–joint shared clones, 36 were also detected in blood (“triple shared clones”; Figure 4D). In PsA patient 4 (who received a single dose of bimekizumab the week before participating in the study), some triple shared clones were very highly expanded in the blood. For example, the most expanded triple shared clone comprised 124 CD8^+^ T cells from the blood, 48 from the joint, and 4 from the skin. However, most of the 36 triple shared clones contained more cells located within the skin and joint than in blood. Despite the presence of shared clones, the majority of clones in all patients were only detected in a single tissue, and across most patients there were clones that were highly expanded in one tissue but not detected in others (Supplementary Figure S17). This suggests that the detection of clones shared between tissues is not simply related to the increased probability of capturing highly expanded clones.

There were fewer CD4^+^ skin–joint shared clones (Figure 4A). A potential reason for reduced clone sharing by CD4^+^ T cells is that CD4^+^ T cells are more polyclonal than CD8^+^ T cells^44^ (compare Figure 4F vs 4E). Therefore, the likelihood of capturing two cells belonging to the same clone is lower for CD4^+^ T cells than for CD8^+^ T cells. Overall, a median of 13% of the skin and 8% of the synovial CD8^+^ TCR repertoire and 6% of the skin and 1% of the synovial CD4^+^ TCR repertoire was made up of shared clones (Figure 4G and H).

**Figure 5:**
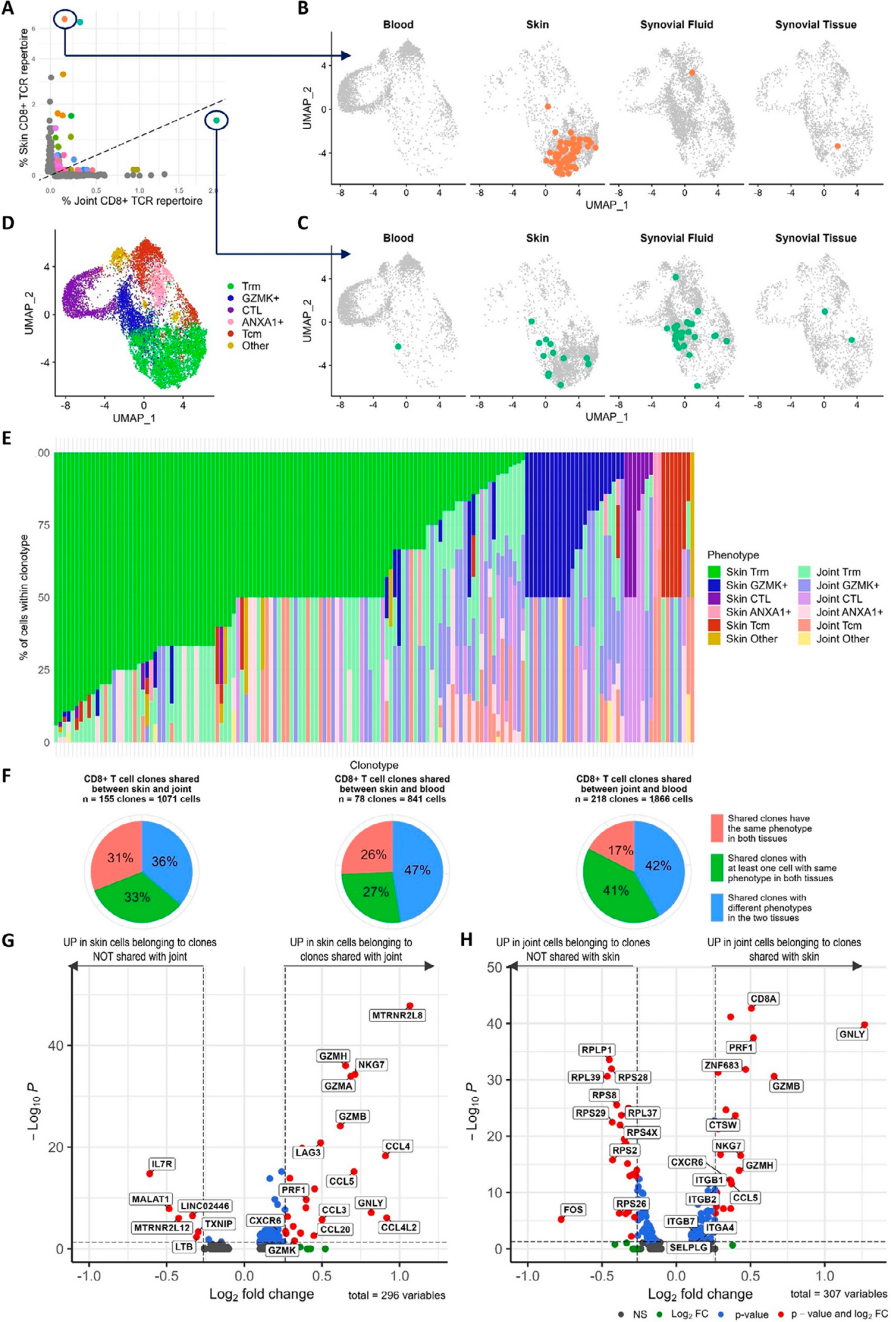
Signature of CD8^+^ skin–joint shared clones (A) Scatterplot showing frequency of CD8^+^ T cell clones in the skin and joint from one patient (PsA patient 3). (B and C) Circled clones from panel A are highlighted on UMAPs. (D) UMAP with clusters grouped into meta‐clusters with similar phenotypes. Clusters not defined as Trm, GZMK^+^, CTL, *ANXA1* ^+^, or Tcm cells are grouped as “other.” (E) Bar chart depicting meta‐cluster location and tissue origin of cells in each of the 155 skin–joint shared clones. Each vertical bar represents a CD8^+^ T cell clone. Color represents meta‐cluster and tissue location of cells within that clone. (F) Pie charts indicating similarity of skin–joint, skin–blood and joint–blood shared clones. Clones are defined as having the same (all cells located in the same meta‐cluster in both compartments), different (cells located in different meta‐clusters), or similar (at least one cell from each compartment located within the same meta‐cluster) signatures in the two compartments. (G and H) Volcano plot showing differentially expressed genes between skin (G) and joint (H) cells belonging to skin–joint shared clones versus non shared clones. Low frequency of shared clones in some patients limited utility of FindConservedMarkers(); therefore, differentially expressed genes were calculated with the Wilcoxon signed rank test using FindMarkers(). CTL, cytotoxic lymphocyte; NS, not significant; PsA, psoriatic arthritis; Tcm, central memory T; Trm, tissue‐resident memory T; UMAP, uniform manifold approximation and projection. Color figure can be viewed in the online issue, which is available at http://onlinelibrary.wiley.com/doi/10.1002/art.43286/abstract.

We mapped CD8^+^ skin–joint shared clones back onto the UMAP to identify which clusters they were associated with (Figure 5A–D). Skin–joint shared clones tended to have a similar signature in the skin and the joint, with the majority located in clusters with a Trm or GZMK^+^ signature (Figure 5E). Cells from 31% of skin–joint shared CD8^+^ clones had the same signature in the skin and joint (defined as all cells of that clone falling within the same meta‐cluster in both the skin and joint), 33% of clones had a similar signature in the skin and joint (defined as clones with at least one cell from the skin and one from the joint falling within the same meta‐cluster), and 36% had completely different signatures (Figure 5F).

Skin–joint shared clones had significantly increased expression of genes associated with cytotoxicity (*GZMB*, *GZMH*, *GNLY*, *PRF1*, *NKG7*) compared to nonshared clones (Figure 5G and H, Supplementary Figure S18). In the joint, shared clones also had up‐regulation of *CXCR6* and the transcription factor *ZNF683* (which encodes HOBIT), both of which are associated with tissue residency, suggesting a stronger Trm phenotype in skin–joint shared clones present in the joint compared to nonshared clones (Figure 5H). The integrins *ITGB1* and *ITGB2* were also significantly increased in joint cells belonging to shared clones (Figure 5H). We investigated the expression of the skin‐homing receptor cutaneous lymphocyte‐associated antigen (CLA) (encoded by *SELPLG*) and the gut‐homing receptor α4β7 (encoded by *ITGA4, ITGB7*) in shared versus nonshared clones. Expression of both receptors was slightly but significantly increased in shared clones in the joint compared to nonshared clones (fold change < 1.2; Figure 5H).

### CD4 ^+^ Trm cell frequency and subset composition in the skin and the joint is different from CD8 ^+^ Trm cells

**Figure 6:**
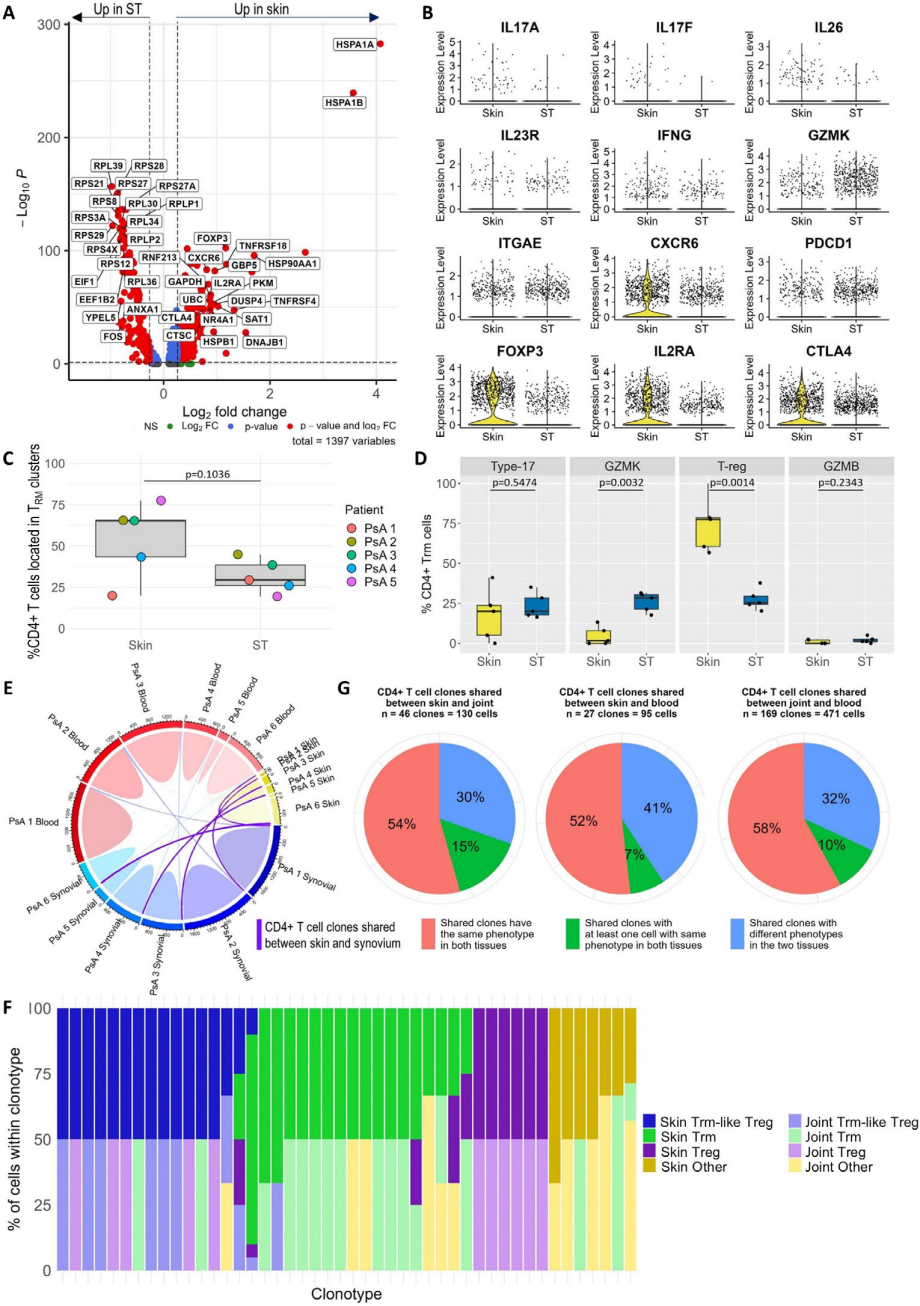
Signature of memory CD4^+^ T cells in PsA. (A) Volcano plot depicting differentially expressed genes between memory CD4^+^ T cells from paired skin epidermis and ST samples (n = 5). Differential expression of pooled cells from all patients were calculated with the Wilcoxon signed rank test using FindMarkers() applied to SCTransformed RNA. The top 20 increased and decreased genes that were also identified as significantly differentially expressed by FindConservedMarkers() or in three or more of five patients after applying FindAllMarkers to each patient individually are labeled. (B) Violin plots comparing expression of specified genes in skin (n = 6) and ST (n = 5) samples. (C) The percentage of CD4^+^ memory T cells from paired skin and ST samples (n = 5) located in Trm cell clusters. (D) The percentage of CD4^+^ Trm cells belonging to each Trm subset. (E) Circle plots visualizing sharing of CD4^+^ T cell clones between the skin (yellow bar), joint (blue), and blood (red). The size of the circumferential bar is proportional to the number of TCRs. Skin–joint, skin–blood, and joint–blood shared clones are represented with purple, red, and blue lines, respectively. (F) Bar chart depicting meta‐cluster location and tissue origin of cells in each of the 46 skin–joint shared CD4^+^ T cell clones. Clusters not defined as Trm, Trm‐like Treg, or Treg are grouped as “other.” (G) Pie charts indicating similarity of skin–joint, skin–blood, and joint–blood shared clones. Boxplots show the median ± interquartile range (paired *t*‐tests; two‐tailed, n = 5). NS, not significant; PsA, psoriatic arthritis; ST, synovial tissue; TCR, T cell receptor; Trm, tissue‐resident memory T. Color figure can be viewed in the online issue, which is available at http://onlinelibrary.wiley.com/doi/10.1002/art.43286/abstract.

In contrast to CD8^+^ T cells, there was no difference in *IL17A* expression between CD4^+^ T cells from the lesional skin epidermis and ST. Instead, there was significant up‐regulation of *FOXP3*, *IL2RA*, and *CTLA4* in the skin compared to the joint, suggesting a stronger Treg signature (Figure 6A and B, Supplementary Data 4). Seurat clustering yielded 17 clusters (Supplementary Figure S19A). Similar to CD8^+^ T cells, skin and blood CD4^+^ T cells clustered separately from each other on the UMAP, whereas ST and SF T cells were spread more diffusely across the UMAP, indicating a broader range of signatures within CD4^+^ T cells from the joint than those from either blood or skin (Supplementary Figure S19B).

Analysis of differentially expressed genes between clusters (Supplementary Figure S20, Supplementary Data 5) enabled grouping of clusters with similar gene signatures, including Trm cells (clusters 6, 9, 10, and 15) and Treg cells, both with (clusters 11 and 13) and without (clusters 2 and 12) a Trm‐like signature (Supplementary Figure S19C and D). CD4^+^ Trm cell clusters were identified and characterized using the same approach as for CD8^+^ T cells (Supplementary Figure S19E–G). Unlike CD8^+^ Trm cells, there was no significant difference in the frequency of CD4^+^ Trm cells between the skin epidermis and ST (Figure 6C). Examination of gene expression (Supplementary Figure S19F) and GSEA comparing each CD4^+^ Trm cluster to pooled cells from other Trm clusters (Supplementary Figure S19G) identified four Trm subsets: type 17 (cluster 9), CD49a^+^ *GZMK* ^+^ (cluster 6), Treg cells (clusters 11 and 13), and a small cluster of CD49a^+^ *GZMB* ^+^ Trm cells (cluster 15). Type 17 (cluster 9) and Treg (clusters 11 and 13) Trm subsets were also significantly positively enriched for signatures of peripheral helper T cells^45^ (Supplementary Figure S19G). There was also a Trm cluster with a mixed signature (cluster 10). The frequency of type 17 CD4^+^ Trm cells was similar in the skin and ST, whereas CD49a^+^GZMK^+^ Trm cells were increased in ST, and Treg Trm‐like cells were increased in the skin (Figure 6D).

Across all patients, there were 46 CD4^+^ skin–joint shared clones comprising 130 cells (Figures 4A and 6E). Most shared clones had a Trm or Treg Trm‐like signature, and as with CD8^+^ T cells, cells belonging to CD4^+^ shared clones tended to have the same signature in the skin and the joint (Figure 6F and G).

## DISCUSSION

In this study, we demonstrate shared T cell clonality and signatures between inflamed skin and joints in PsA. The colocalization of epidermal CD8^+^ Trm cells with macrophages and Langerhans cells and the potential for T cell costimulation through ligand–receptor interaction support antigen‐driven activation of CD8^+^ Trm cells in the skin. The identification of CD8^+^ skin–joint shared T cell clones supports the hypothesis that the same antigen is present in both sites and/or that CD8^+^ T cells migrate between the skin and joint to propagate inflammation across both sites. Despite differences in the overall gene signature of CD8^+^ T cells in the skin and joint, skin–joint shared clones tend to have similar signatures in both sites, characterized by increased expression of genes associated with cytotoxicity (*GZMB*, *GNLY*, *PRF1*, *GZMH*, *NKG7*). This could indicate increased cytotoxic potential of shared clones and suggests that they are likely to contribute to inflammation.

It is highly unlikely that two independent T cell precursors would rearrange their TCR into the exact same sequence.^46^ Thus, two T cells expressing the same TCR sequence in the skin and joint in PsA will almost certainly be descended from the same parent T cell. Therefore, the identification of activated shared T cell clones in the skin and joints of patients with PsA supports the hypothesis that skin and joint inflammation in PsA is linked, thereby confirming and extending previous work.^17^, ^18^, ^22^, ^23^, ^24^

A recent study of patients with ankylosing spondylitis (AS) and patients with anterior uveitis, which share many clinical, genetic, and immunologic characteristics with PsA, identified CD8^+^ T cell clones that are shared between the eye and joint.^47^ Analysis of TCR and HLA–B27 interactions identified potential self and/or microbial antigens that might drive both joint and eye inflammation in AS.^47^ This raises the possibility of (self) antigen‐driven recruitment and/or activation of T cells in the skin and joint in PsA. Two putative autoantigens have been reported in psoriasis, the melanocyte peptide ADAMTSL5^48^ and the antimicrobial peptide LL37.^49^ Both peptides are presented by the psoriasis‐associated HLA–C*06:02, activate T cells in psoriatic skin, and can induce type 17 responses.^48^, ^49^, ^50^ Although Trm cells are typically described as permanently resident within tissue, a subset can egress from tissue and travel in the blood to establish residency at distant sites or even transdifferentiate into different memory subtypes.^51^, ^52^, ^53^, ^54^, ^55^, ^56^ Furthermore, studies in mice have shown that antigen‐independent inflammation in tissue is sufficient to drive T cell recruitment and Trm cell differentiation.^54^, ^57^ Thus, inflammation of a joint arising from biomechanical injury may nonspecifically recruit circulating ex‐skin Trm T cells into the joint, where they could take up residence and drive the development and/or persistence of PsA.

The finding that skin CD8^+^ T cells have a stronger IL‐17 signature than those from the joint correlates with clinical trial data and real‐world experience that IL‐17 inhibition produces a more profound improvement in psoriatic skin inflammation than in joint inflammation.^9^, ^10^ It is also consistent with previous studies comparing skin and synovium by bulk RNAseq, which reported stronger IL‐17/IL‐23 signatures in the skin.^29^, ^30^ Despite the stronger IL‐17 signature in the skin, CD8^+^ type 17 Trm cells are present in the joint at frequencies consistent with previous work.^6^, ^7^ Our findings therefore suggest that although IL‐17 may be a dominant driver of inflammation in the skin, other factors, in addition to IL‐17, contribute to driving inflammation in the joints. This may explain why blocking IL‐17 is often less effective at reducing joint inflammation than skin inflammation. Treatment of refractory PsA may require combination therapy with drugs that block multiple cytokines (eg, IL‐17 and TNF) or entirely novel targets such as GZMK. CD8^+^GZMK^+^ T cells have been detected in inflamed synovium in rheumatoid arthritis, the inflamed bowel in Crohn disease and ulcerative colitis, and in labial glands in primary Sjögren disease,^58^, ^59^ suggesting these cells may drive inflammation in multiple diseases and across multiple tissues.

Our analysis also identified four subsets of CD4^+^ Trm cells: (1) CD4^+^ type 17 Trm cells present at equal frequencies in the skin and joint, (2) CD4^+^ CD49a^+^GZMK^+^ Trm cells enriched in the joint, (3) CD4^+^ Treg Trm‐like cells enriched in the skin, and (4) a small population of CD4^+^ CD49a^+^GZMB^+^ Trm cells. scRNAseq enabled identification of CD103^−^CD4^+^ Trm cells, which extends our previous work using cytometry by time‐of‐flight (CyTOF) mass spectrometry, which identified only one cluster of CD103^+^CD69^+^CD4^+^ Trm cells.^7^ In humans, CD4^+^ Trm cells have a higher propensity to egress from the skin and migrate to distant sites than CD8^+^ Trm cells.^54^, ^56^ Studies in mice show that tissue Treg cells readily recirculate between the vasculature and skin,^60^ and in patients with juvenile idiopathic arthritis, the presence of shared CD4^+^ Treg clones between different joints of the same patient suggests they migrate between tissues in humans.^61^ Most CD4^+^ skin–joint shared clones in our data set had a Trm or Treg signature suggesting that CD4^+^ Trm and/or Treg cells can also migrate between the skin and joint. Because most CD4^+^ T cells in the skin are located in the dermis, further work comparing the TCR repertoire of full‐thickness skin with that of the joint may identify greater numbers of shared CD4^+^ T cell clones.

Our study has some limitations. We enrolled six patients with different disease durations and taking different treatments. Increased patient numbers will be required to confirm that the results can be generalized to the population of patients with PsA. Further work will be required to investigate the importance of GZMK in driving inflammation in joints in PsA and to validate and investigate the functional impact of the ligand–receptor interactions identified between CD8^+^ Trm cells and antigen‐presenting cells in the skin. Lastly, although this work has focused on T cells, other cell types, including other immune cell subsets and stromal cells, are known to contribute to the pathogenesis of psoriasis and PsA.^62^, ^63^

Our finding that CD8^+^ Trm cells, particularly in the skin, colocalize with antigen‐presenting cells supports a role for antigen‐driven activation of T cells in PsA, which may originate in the skin. The presence of activated cytotoxic Trm cells in the joint, which share TCR sequences with their counterparts in the skin, supports the concept that T cells migrate between the skin and the joint. Further sampling and deeper analysis of the antigen/TCR/MHC interactions are necessary and could support the potential for antigen‐specific immunotherapy in PsA to induce long‐term tolerance. Further sophisticated investigation will also be required to confirm whether T cells migrate between the skin and joint in humans and the mechanisms by which this is happening. Nonetheless, the demonstration that skin and joint inflammation in PsA is linked through the presence of phenotypically similar T cell clones will inform novel translational approaches toward treatment of PsA.

## AUTHOR CONTRIBUTIONS

All authors contributed to at least one of the following manuscript preparation roles: conceptualization AND/OR methodology, software, investigation, formal analysis, data curation, visualization, and validation AND drafting or reviewing/editing the final draft. As corresponding author, Dr Taams confirms that all authors have provided the final approval of the version to be published and takes responsibility for the affirmations regarding article submission (eg, not under consideration by another journal), the integrity of the data presented, and the statements regarding compliance with institutional review board/Declaration of Helsinki requirements.

## Supporting information


**Supplementary methods**:

**Supplementary Figures**:

**Supplementary Table 1**:

**Supplementary Table 2**:

**Supplementary Table 3**:

**Supplementary Data 1**:

**Supplementary Data 2**:

**Supplementary Data 3**:

**Supplementary Data 4**:

**Supplementary Data 5**:

**Supplementary Data 6**:

**Supplementary Data 6**:
