# Supplementary material for: Linking Skin and Joint Inflammation in Psoriatic Arthritis through Shared CD8+ T Cell Clones
Source: Arthritis Rheumatol. 2025 Sep 21;78(1):152–65. doi: 10.1002/art.43286 (PMC12854012; doi:10.1002/art.43286)
Supplement: Supplementary file 1 — Supplementary methods: [file ART-78-152-s008.docx]

**Supplementary methods**

**Supplementary Methods Table 1:** Reagents, equipment and analysis tools used to carry out the work described in this manuscript

| Reagent | Manufacturer |
| --- | --- |
| 4′,6-diamidino-2-phenylindole, DAPI | Thermofisher Scientific, Waltham, MA, USA |
| Chromium Next GEM Chip G Single Cell Kit, | 10x Genomics, Pleasanton, CA, USA |
| Chromium Next GEM Single Cell 5' Library and Gel Bead Kit v1.1 |  |
| Chromium Single Cell V(D)J Enrichment Kit, Human T Cell |  |
| Chromium Single Cell 5' Library Construction Kit, |  |
| Chromium Single Cell 5' Feature Barcode  Library Kit |  |
| Single Index Kit T Set A |  |
| Single Index Kit N Set A |  |
| Cell staining buffer | Biolegend, Sandiego, CA, USA |
| Compensation beads | BD Biosciences, Oxford, UK and Miltenyi Biotech, Bergisch Gladbach, Germany |
| Complete culture media:  Roswell Park Memorial Institute medium, RPMI  1% Penicillin & streptomycin  1% L-glutamine | Gibco, Waltham, MA, US |
| Dimethyl sulfoxide, DMSO | Fisher Scientific, Waltham, MA, USA |
| Dispase II | Sigma-Aldrich |
| DNase | Roche, Basel, Switzerland |
| Fc block | Biolegend |
| Fetal bovine serum, FBS | Gibco |
| Hanks Balanced Salt Solution, HBSS | Gibco |
| Liberase | Roche |
| Lymphoprep | Axis Shield, Dundee, Scotland |
| Phosphate buffered saline, PBS | Sigma-Aldrich, St Louis, MO, USA |
| Trypan Blue | Sigma-Aldrich |
| Laboratory items | Manufacturer |
| 10ml Vacutainer blood tubes coated in heparin | BD Biosciences |
| 16G Quick Core biopsy needle | It’s Interventional, Sheffield, UK |
| 2ml cryovials | Corning, NY, USA |
| 50ml sterile falcon tubes | Appleton Woods, Birmingham, UK |
| 6ml Vacutainer serum tubes | BD Biosciences |
| 70μm cell strainer | Corning |
| C-tubes | Miltenyi Biotech |
| Cell-counting chambers | VWR International, Leicestershire, UK |
| Lo-Bind Eppendorfs | Eppendorf, Hamburg, Germany |
| Pasteur pipettes | Starlab, Milton Keynes, UK |
| Skin punch biopsy needles | Kai Medical |
| Sterile disposable forceps | VWR |
| Sterile disposable scalpels | Swann-Morton, Sheffield, UK |
| Equipment | Manufacturer |
| 10x Chromium | 10x Genomics |
| BD FACSAria cell sorter | BD Biosciences |
| gentleMACS dissociator | Miltenyi Biotech |
| Cos-Mx | Nanostring |
| NextSeq2000 | Illumina, San Diego, CA, USA |
| Analysis tools | Manufacturer |
| AtoMx v1.2 | Nanostring |
| CatsCradle v0.99.8 | https://github.com/AnnaLaddach/CatsCradle |
| CosMx_Lite | https://github.com/cancerbioinformatics/CosMx_Lite |
| CellRanger v6.1.1 | 10x Genomics |
| Circlize v0.4.16 | Gu *et al.*^1^ |
| Clustree v0.5.1 | Zappia *et al.*^2^ |
| Flowjo | TreeStarInc |
| Gene Set Enrichment Analysis, GSEA v4.2.3 | Subramanian *et al.*^3^ |
| GraphPad Prism v10 | ThermoFisher |
| Harmony v1.2.0 | Korsunsky *et al.*^4^ |
| Microsoft Excel | Microsoft |
| NoButter | https://github.com/cancerbioinformatics/NoButter |
| R programming v4.2.2 | R Core Team (2022) Vienna, Austria https://www.R-project.org/ |
| RStudio v2022.07.1+554 | RStudio Team (2020). RStudio: Integrated Development for R. RStudio, Boston, MA |
| SCRepertoire | Borcherding *et al.*^5^ |
| Seurat v4.3.0 (scRNAseq) v5 (Spatial transcriptomics) | Satija lab |

# Patients

Six patients with PsA donated paired samples of blood (n=6) inflamed skin (n=6), ST (n=5) and/or SF (n=5) from an inflamed knee joint (**Table S1**). One additional patient donated ST for spatial transcriptomics only. Six patients donated ST by ultrasound guided needle biopsy during which 8-12 biopsies, each measuring ~1x1x2mm, were taken for this study. One patient (PsA 1) donated ST at the time of knee replacement surgery. HLA genotyping was performed by Synnovis at Guy's and St Thomas’ NHS Foundation Trust and was available for 5 of the 7 patients. Patients with HLA genotypes that are associated with psoriasis or PsA are reported in **Table S1**. Of note, PsA 1 and PsA 5 expressed the major risk allele for psoriasis, *HLA-C*06:02*; PsA 1 and PsA 3 expressed the haplotype *HLA-B*08:01-HLA-C*07:01* which is associated with severe PsA; and PsA 4 and PsA 6 expressed *HLA-B*38:01-HLA-C*12:03* which has also been associated with PsA in some studies^6–8^.

# Sample collection

Peripheral blood (PB) was collected in Vacutainer blood tubes coated in heparin. Synovial fluid (SF) was collected in sterile plastic pots. Ultrasound guided ST biopsies from inflamed knees (8-12 biopsies per patient) were performed using a 16G Quick Core biopsy needle. 6-9 biopsies from each patient were placed in an Eppendorf containing 1ml serum-free RPMI media supplemented with 1% penicillin, streptomycin and L-glutamine and the remaining 2-3 biopsies were placed in 4% formalin. Skin punch biopsies (4-6mm diameter) were obtained from lesional psoriatic skin and collected in Eppendorfs containing 1ml 1% Dispase II in Hanks Balanced Salt Solution (HBSS). ST samples from the patient undergoing orthopaedic surgery were obtained at the time of surgery and collected in a sterile plastic pot.

# Sample processing

## Dissociation

In the five patients where ST was obtained by ultrasound-guided needle biopsy, samples were processed immediately and cells were loaded onto the 10x Genomics chip within 8 hours of collection. Peripheral blood and SF mononuclear cells (PBMC and SFMC respectively) were isolated by density gradient centrifugation using Lymphoprep^TM^. 6-9 ST biopsies were placed directly into serum-free RPMI media containing 0.3mg/ml Liberase and 0.1mg/ml DNase and processed using a custom 30-minute programme on the gentleMACS dissociator which combines enzymatic and mechanical digestion to extract cells from tissue. The reaction was then quenched by the addition of 2.5ml RPMI media supplemented with 10% fetal bovine serum and 1% penicillin, streptomycin and L-glutamine and samples were strained. Skin punch biopsies were incubated at 37°c in 10mg/ml Dispase II in Hanks Balanced Salt Solution for one hour. The epidermis was then peeled off and processed using the gentleMACS dissociator as described above for ST.

In the patient where ST was obtained at knee replacement surgery, ST was minced into ~1-2mm^3^ sized pieces and PBMC and SFMC were isolated and skin epidermis separated from the dermis as described above. Samples were then cryopreserved in culture media containing 45% FCS and 10% DMSO in liquid nitrogen until use. Upon thawing, all subsequent steps were the same as those described above.

## Sample staining

Following cell isolation, PBMC, SFMC and ST and skin epidermis digests underwent identical protocols for staining and sorting. Cells were transferred to LoBind Eppendorfs, washed in PBS, resuspended in 50ul/staining volume of Fc block mastermix (5ul Fc block and 45ul cell staining buffer; one staining volume = up to 2 x 10^6^ cells) and incubated at room temperature for 10 minutes. Following this, 49ul/staining volume of staining mastermix (containing fluorescently labelled antibodies and Cellular Indexing of Transcriptomes and Epitopes by sequencing (CITE-Seq) antibodies, **Table S1**) and 1ul/staining volume of hashtag TotalSeqC antibody master-mix (**Table S2**) was added to each sample and cells were incubated for 30 minutes at 4°C. For each patient, the two samples with the highest cell number were stained in duplicate to enable the use of 6 separate hashtags across the 4 samples to enhance doublet detection (**Table S2**). Cells were then washed twice in cell staining buffer and a third time in PBS containing 1% FCS, then resuspended in 300ul PBS containing 1% FCS and transferred to sterile FACS tubes. Replicate cells from the same tissue which were stained with different hashtags were pooled at this point.

## Fluorescent-Activated Cell Sorting

Memory T-cells (CD45RA-CD27+, CD45RA-CD27+ and CD45RA+CD27-) were sorted on BD Aria cell sorter using an 85um nozzle for ST and skin epidermis digests and a 70um nozzle for PBMC and SFMC. Gating strategy is shown in **Figure S1**. DAPI was added to label non-viable cells immediately prior to sorting. Memory T-cells from all four tissue compartments were pooled in a single LoBind Eppendorf that had been pre-coated with FCS and were retained on ice pending library preparation.

## Single cell library preparation and sequencing

A maximum of 20,000 sorted memory T-cells per well were immediately loaded onto the 10x Genomics chip. Single cell libraries were created using the Chromium Single-Cell 5’ Reagent Kits v1.1, Libraries were sequenced on the NextSeq 2000 platform.

## Spatial transcriptomics

Spatial transcriptomics was performed on sections from formalin fixed paraffin embedded biopsies from paired skin and ST from one patient and unpaired skin and ST from one and three patients respectively (**Supplementary Table 1**). This was done using the Nanostring CosMx 1000-plex panel according to manufacturers’ instructions.

# Data analysis

## Single cell RNA sequencing

### Pre-processing of single cell RNAseq data and QC

Raw reads were aligned to the human transcriptome and “multi” option of CellRanger software package (v6.1.1) with default parameters. Outputs from CellRanger were loaded into Seurat. TCR genes were removed from the RNA assay and added to the Seurat object as a separate assay (“TCR assay”). This was to prevent TCR genes impacting on sample integration or cell clustering. Samples were demultiplexed (using MULTIseqDemux()). Poor quality cells (defined as cells with very high or low numbers of genes (nFeature_RNA), very high or low number of absolute RNA counts (nCount_RNA), a high percentage of mitochondrial genes or absolute ADT counts > 10,000 (which likely indicates the presence of a clump of CITE-seq antibodies)) were removed (**Figure S2**). The thresholds for nFeature_RNA, nCount_RNA and %mitochondrial QC metrics were defined as three times the mean absolute deviation of each metric in each independent sample^9^. The advantage of calculating automatic thresholds for each sample rather than using a “one size fits all” threshold for all samples is that automatically calculated thresholds take into account variation between samples. Doublets, which were identified by MULTIseqDemux and/or cells which expressed more than one TCRβ gene or more than two TCRα genes, were also removed. An additional QC step was performed for PsA 5 which, despite high cell viability during cell sorting, had higher ambient RNA in the gene expression library. For this sample, only cells which had paired TCR sequences (and therefore most likely represented true, viable cells) were taken forward to QC.

### Single cell RNAseq data analysis

Single cell RNAseq data was analysed in R using Seurat. The 7 datasets (two libraries were created for PsA 3, one library for each of the other 5 patients) were individually normalised using SCTransform v2 with regression of cell cycle score, the percentage of mitochondrial genes and the digestion module score^10^ (RNA assay) and NormalizeData() and ScaleData (ADT and TCR assays) and integrated^11,12^. The digestion module score was created using the list of 512 genes which comprised the “core digestion signature” reported in ^13^. Dimensionality reduction was performed using the integrated RNA assay to calculate 30 principal components and construct a SNN graph using 30 dimensions. Clustering was performed on the integrated assay using resolution 0.9 with otherwise default parameters yielding 24 clusters. Clustree analysis was used to guide selection of resolution for clustering^2^. Marker expression of resultant clusters was then explored and compared with published datasets to ensure biological plausibility of the selected resolution.

### Identification of CD8+ and CD4+ T-cells

CD8+ and CD4+ T-cells were extracted from the integrated dataset and analysed separately. Clusters were broadly classified as “CD4+” (clusters 0, 1, 3, 7, 8, 12, 13, 15 and 22), “CD8+” (clusters 2, 4, 5, 6, 10, 14, 17, 18, 19, 20 and 21) or “mixed” (clusters 9, 11, 16, and 23) based on expression of *CD4* and *CD8A* (**Figure S3C**). However, some clusters, for example cluster 6, predominantly comprised *CD8A*+ cells but did contain some cells that expressed *CD4* (**Figure S3D**). Similarly, there were *CD4*+ clusters which contained some cells that expressed *CD8A.* Therefore, *CD8A+CD4-* cells which were located in CD4+/mixed clusters were extracted and added to the CD8+ T-cell dataset. And, *CD4+CD8A-* cells which were located in CD8+/mixed clusters were extracted and added to the CD4+ T-cell dataset. The separate CD8+ and CD4+ datasets were then split up into their constituent sample libraries and re-normalised, integrated and clustered (with resolution 1 and 0.9 for the CD8+ and CD4+ dataset respectively) using the same approach as described above.

### Differential Gene Expression

Wilcoxon rank sum tests were performed using the SCTransformed assays to identify differentially expressed genes for each cluster/tissue using the FindConservedMarkers(), FindMarkers() and FindAllMarkers() functions. Combined p values (FindConservedMarkers) and adjusted p-values (FindMarkers and FindAllMarkers) <0.05 were considered statistically significant. FindMarkers/FindAllMarkers pools cells from all samples in an integrated object and therefore, the results may be subject to batch effect between samples or be skewed by one sample. In contrast, FindConservedMarkers() performs differential gene expression testing for each sample (in this case, patient) separately and combines the p-values using meta-analysis methods from the MetaDE R package. For this reason, where feasible, we report differentially expressed genes that are identified by either FindConservedMarkers() or which are significant in the majority of patients when FindMarkers/FindAllMarkers is performed on each patient individually.

### Gene Set Enrichment Analysis (GSEA)

GSEA was performed using GSEA v4.2.3^3^. Lung CD4+ and CD8+ core T_RM_ signatures were obtained from Kumar et al ^14^. With regards to other gene signatures used for GSEA, signatures from psoriatic skin T-cell subsets comprised T_RM_, Tc1, Tc17 and Tc17/Tc22 signatures from ^15^, CD49a+ and CD49a- T_RM_ signatures from ^16^, and psoriatic and healthy skin type-17 CD4+ and CD8+ T_RM_ cells and psoriatic and healthy skin CD4+ and CD8+ T_REG_s from ^17^. Signatures from PsA synovial fluid T-cell subsets comprised type-17 and CD49a+GZMK+ T_RM_ signatures from our previous work^18^, CD8+ HLA-DR high T-cells from ^19^, canonical T_REG_s and CCR4+Helios+ T_REG_s signatures from ^20^, and T_PH_ cells from ^21^.

### Single cell TCR sequencing analysis

TCR repertoire analysis was performed in R using the scRepertoire and circulize packages^1,5^.

## Spatial transcriptomics

### Data preprocessing

Raw data was obtained from AtoMx, with default cell segmentation parameters. Raw transcript file was used to perform z-stack cleaning using NoButter package. Z-stack 7 was removed as it contained highest levels of transcripts outside of cells, and subsequently the gene expression matrix was reconstructed using cleaned transcript file. CosMx-lite pipeline, based on Seurat R package was used to analyse data in R. Datasets were filtered to contain cells with at least 10 transcript and less than 5% of negative probes. SCTransform() function was used for normalization with default parameters.

### Cell annotation

First, each section was analysed independently, and canonical gene expression was used to identify broad cell types including immune cells, fibroblasts, endothelial/smooth muscle cells and keratinocytes (**Figure S8, S9**). Then, cells from all 6 sections which belonged to the same broad cell type, were integrated using Harmony with batch correction using theta = 2. Dimensionality was then reduced using RunUMAP() and FindClusters() was applied. Clustree^2^ analysis was used to guide selection of resolution for clustering and otherwise clustering used default parameters. Resolution was set as 0.6 for immune cells, 0.5 for keratinocytes, 0.4 for ST fibroblasts and 0.4 for dermal fibroblasts. Wilcoxon rank sum tests were performed using the SCTransformed assays to identify differentially expressed genes for each cluster/tissue using the FindMarkers(). Specific cell subsets were then identified based upon differentially expressed genes between clusters and expression of canonical genes (**Figures S10, S11).**

To enable more granular annotation of T-cells and myeloid cells, clusters with a gene signature consistent with either a T-cell or myeloid cell phenotype were subsetted from the integrated immune cell analysis. The separate T-cell and myeloid cell datasets were then split up into their constituent sample libraries and re-normalised and integrated. The integrated T-cell dataset and myeloid dataset were then clustered using resolution 0.9 and 0.5 respectively. The myeloid cell clusters were then annotated using the same approach as described above (**Figure S11A**).

### Identification of CD8+ and CD4+ T_RM_ cell clusters

To identify CD4+ and CD8+ T-cells in the CosMx dataset, FindTransferAnchors() and TransferData() were used to project the integrated T-cell (CosMx) dataset on to the single cell RNA sequencing total memory T-cell Seurat object (**Figure S10C, D**). Cells in the CosMx integrated T-cell dataset that were predicted to be CD8+ T-cells were then subsetted, split up into their constituent sample libraries and re-normalised, integrated and clustered using resolution 0.9.

AddModuleScore() was then applied to the CosMx CD8+ T-cell dataset using the gene signatures from CD8+ T_RM_ cell subsets identified by single cell RNA sequencing (see section 4.2.4). AddModuleScore() calculates the average expression levels of each gene signature on a single cell level, subtracted by the aggregated expression of control feature sets. Cluster 1 in the CosMx CD8+ T-cell dataset contained cells that had high expression of genes contained within all CD8+ T_RM_ cell subset gene signatures (**Figure S10G**). However, it was not possible to differentiate individual CD8+ T_RM_ cell subsets within the CosMx dataset. Therefore, cells located in cluster 1 were annotated as CD8+ T_RM_ cells and cells located in all other clusters were annotated as CD8+ non-T_RM_ cells.

The same approach was taken to identify CD4+ T_RM_ cells in the CosMx dataset. Cells in the CosMx integrated T-cell dataset that were predicted to be CD4+ T-cells were subsetted, split up into their constituent sample libraries and re-normalised, integrated and clustered using resolution 0.7. AddModuleScore() was then applied using the gene signatures from the CD4+ T_RM_ cell subsets identified in the single cell RNA sequencing dataset (see section 4.2.4). Clusters 1, 3 and 7 had highest expression of the global CD4+ T_RM_ cell signatures and therefore were classified as CD4+ T_RM_ cell clusters (**Figure S10H**).

### T_RM_ gene signatures from single cell RNA sequencing dataset

Gene expression signatures for each CD8+ T_RM_ subset identified in the single cell RNA sequencing analysis were calculated as described below:

- Global CD8+ T_RM_ signature:

Genes significantly upregulated in CD8+ T_RM_ cells vs. CD8+ non T_RM_ cells

- CD8+ type-17 T_RM_ signature:

Genes significantly upregulated in Type-17 CD8+ T_RM_ cells vs. GZMK+ cytotoxic CD8+ T_RM_ cells

- CD8+ GZMK+ T_RM_ signature:

Genes significantly upregulated in GZMK+ cytotoxic CD8+ T_RM_ cells vs. Type-17 CD8+ T_RM_ cells

- CD8+ cytotoxic type-17 T_RM_ signature:

Genes significantly upregulated in CD49a+ cytotoxic type-17 CD8+ T_RM_ cells vs. Type-17 and GZMK+ cytotoxic CD8+ T_RM_ cells

Gene expression signatures for each CD4+ T_RM_ subset identified in the single cell RNA sequencing analysis were calculated as described below:

- Global CD4+ T_RM_ signature:

Genes significantly upregulated in CD4+ T_RM_ cells vs. CD4+ non T_RM_ cells

- CD4+ type-17 T_RM_ signature:

Genes significantly upregulated in Type-17 CD4+ T_RM_ cells vs. GZMK+ CD4+ T_RM_ cells

- CD4+ GZMK+ T_RM_ signature:

Genes significantly upregulated in GZMK+ CD4+ T_RM_ cells vs. Type-17 CD4+ T_RM_ cells

- CD4+ T_REG_ T_RM_ signature:

Genes significantly upregulated in T_REG_ T_RM_ cells vs. all other T_RM_ cells

### Cell neighbourhood analysis

Cells from each individual tissue were named according to the annotations allocated by the analyses described above. Cell neighbourhood analysis was then performed using CatsCradle using default parameters. For ligand-receptor analysis, CatsCradle employs the curated list of ligand-receptor interactions from Nichenet^22^.

## Statistical analysis

Statistical analysis was performed in R and GraphPad Prism. Statistical tests, p values and number of samples are detailed in relevant figure legends. Detailed results of statistical analysis are reported in **Supplementary Data 6**.

# Data availability

Single cell RNA sequencing data is available in the Gene Expression Omnibus (GEO) database^23^: [GSE250242](https://www.ncbi.nlm.nih.gov/geo/query/acc.cgi?acc=GSE250242) **(PsA 1, 2, 3, 4) and** [GSE250243](https://www.ncbi.nlm.nih.gov/geo/query/acc.cgi?acc=GSE250243) (PsA 5, 6). An independent analysis of blood, SF and ST T-cells from patients PsA 1, 2, 3, 4 in combination with two additional patients has been published separately^10^.

1. Gu, Z., Gu, L., Eils, R., Schlesner, M. & Brors, B. circlize implements and enhances circular visualization in R. *Bioinformatics* **30**, 2811–2812 (2014).

2. Zappia, L. & Oshlack, A. Clustering trees: a visualization for evaluating clusterings at multiple resolutions. *Gigascience* **7**, 1–9 (2018).

3. Subramanian, A. *et al.* Gene set enrichment analysis: a knowledge-based approach for interpreting genome-wide expression profiles. *Proc Natl Acad Sci U S A* **102**, 15545–15550 (2005).

4. Korsunsky, I. *et al.* Fast, sensitive and accurate integration of single-cell data with Harmony. *Nature Methods 2019 16:12* **16**, 1289–1296 (2019).

5. Borcherding, N. & Bormann, N. L. scRepertoire: An R-based toolkit for single-cell immune receptor analysis. *F1000Res* **9**, 47 (2020).

6. Haroon, M., Winchester, R., Giles, J. T., Heffernan, E. & FitzGerald, O. Certain class I HLA alleles and haplotypes implicated in susceptibility play a role in determining specific features of the psoriatic arthritis phenotype. *Ann Rheum Dis* **75**, 155–162 (2016).

7. Winchester, R. *et al.* HLA associations reveal genetic heterogeneity in psoriatic arthritis and in the psoriasis phenotype. *Arthritis Rheum* **64**, 1134–1144 (2012).

8. Winchester, R. *et al.* Implications of the diversity of class I HLA associations in psoriatic arthritis. *Clinical Immunology* **172**, 29–33 (2016).

9. Siu, J. H. Y. *et al.* Two subsets of human marginal zone B cells resolved by global analysis of lymphoid tissues and blood. *Sci Immunol* **7**, eabm9060 (2022).

10. Durham, L. E. *et al.* Substantive Similarities Between Synovial Fluid and Synovial Tissue T cells in Inflammatory Arthritis Via Single-Cell RNA and T cell Receptor Sequencing. *Arthritis & Rheumatology* (2024) doi:10.1002/ART.42949.

11. Choudhary, S. & Satija, R. Comparison and evaluation of statistical error models for scRNA-seq. *Genome Biol* **23**, 1–20 (2022).

12. Stuart, T. *et al.* Comprehensive Integration of Single-Cell Data. *Cell* **177**, 1888-1902.e21 (2019).

13. O’Flanagan, C. H. *et al.* Dissociation of solid tumor tissues with cold active protease for single-cell RNA-seq minimizes conserved collagenase-associated stress responses. *Genome Biol* **20**, 210 (2019).

14. Kumar, B. V. *et al.* Human Tissue-Resident Memory T Cells Are Defined by Core Transcriptional and Functional Signatures in Lymphoid and Mucosal Sites. *Cell Rep* **20**, 2921–2934 (2017).

15. Liu, J. *et al.* Single-cell RNA sequencing of psoriatic skin identifies pathogenic Tc17 cell subsets and reveals distinctions between CD8+ T cells in autoimmunity and cancer. *Journal of Allergy and Clinical Immunology* **147**, 2370–2380 (2021).

16. Cheuk, S. *et al.* CD49a expression defines tissue-resident CD8+ T cells poised for cytotoxic function in human skin. *Immunity* **46**, 287–300 (2017).

17. Cook, C. P. *et al.* A single-cell transcriptional gradient in human cutaneous memory T cells restricts Th17/Tc17 identity. *Cell Rep Med* **3**, 100715 (2022).

18. Povoleri, G. *et al.* Psoriatic and rheumatoid arthritis joints differ in the composition of CD8+ tissue-resident memory T-cell subsets. *Cell Rep* **42**, 112514 (2023).

19. Penkava, F. *et al.* Single-cell sequencing reveals clonal expansions of pro-inflammatory synovial CD8 T cells expressing tissue-homing receptors in psoriatic arthritis. *Nat Commun* **11**, 4767 (2020).

20. Simone, D. *et al.* Single cell analysis of spondyloarthritis regulatory T cells identifies distinct synovial gene expression patterns and clonal fates. *Commun Biol* **4**, 1395 (2021).

21. Argyriou, A. *et al.* Single cell sequencing identifies clonally expanded synovial CD4+ TPH cells expressing GPR56 in rheumatoid arthritis. *Nat Commun* **13**, 4046 (2022).

22. Browaeys, R., Saelens, W. & Saeys, Y. NicheNet: modeling intercellular communication by linking ligands to target genes. *Nature Methods 2019 17:2* **17**, 159–162 (2019).

23. Barrett, T. *et al.* NCBI GEO: archive for functional genomics data sets—update. *Nucleic Acids Res* **41**, D991–D995 (2013).
