# Supplementary material for: Linking Skin and Joint Inflammation in Psoriatic Arthritis through Shared CD8+ T Cell Clones
Source: Arthritis Rheumatol. 2025 Sep 21;78(1):152–65. doi: 10.1002/art.43286 (PMC12854012; doi:10.1002/art.43286)
Supplement: Supplementary file 2 — Supplementary Figures: [file ART-78-152-s012.pdf]

Fig. S1 Durham et al.

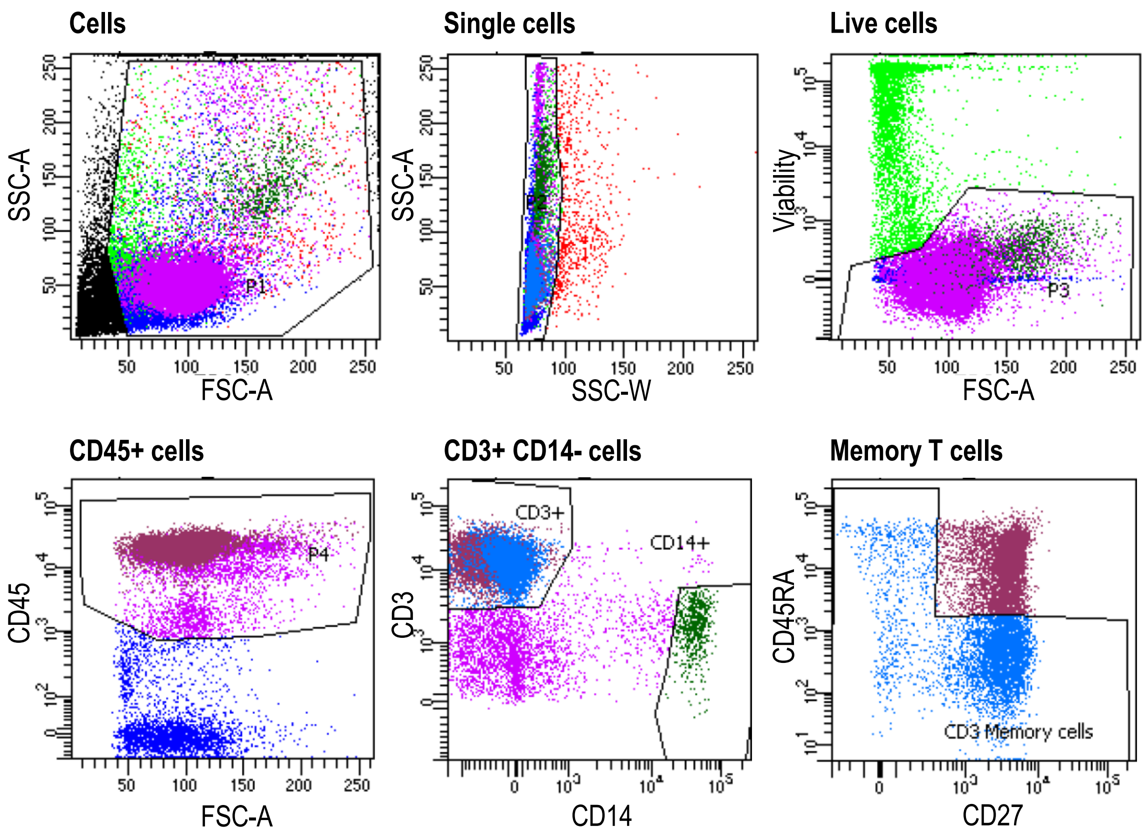

Figure S1: Gating strategy for the identification of memory T-cells.
Gating strategy used to sort live memory (CD45RA-CD27-, CD45RA-CD27+ and CD45RA+CD27-) T-cells from PBMC, SFMC, skin epidermis and synovial tissue digests. Cells were stained with fluorescent-conjugated antibodies and CITE-seq antibodies prior to sorting. DAPI was added immediately prior to sorting to enable exclusion of dead cells. The gating strategy is shown for cells (FSC-A vs SSC-A), singlets (SSC-W vs SSC-A), live cells (DAPI-), CD45+ cells, CD3+CD14- cells and CD45RA-CD27-/CD45RA-CD27+/CD45RA+CD27- memory T-cells. Representative example using PBMC from patient PsA 2.

Fig. S2 Durham et al.

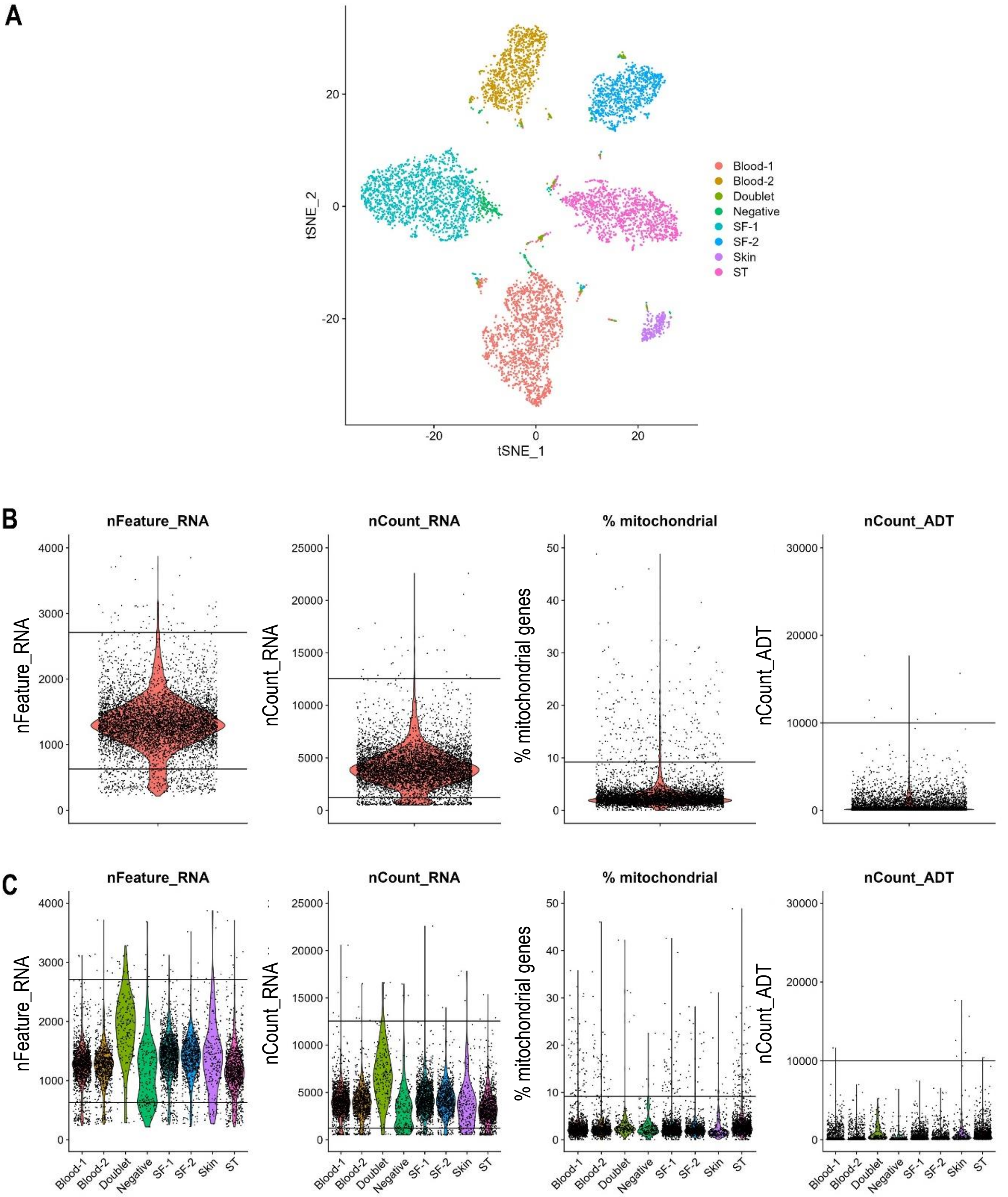

Figure S2: Quality control of single cell sequencing.
QC plots from one representative patient (PsA 2). Samples were demultiplexed then filtered to remove poor quality cells (defined as cells with very high or low numbers of genes, very high or low number of absolute counts or a high percentage of mitochondrial genes). The thresholds for nFeature_RNA, nCount_RNA and %mitochondrial QC metrics were defined as three times the mean absolute deviation of each metric in each independent sample[65]. **A)** tSNE plot showing the results of de-multiplexing the hashtags. Cells coloured by assigned identity. Blood and synovial fluid were split in half and each half was stained with a different hashtag. This was to increase the overall number of hashtags and thereby improve doublet detection. **B)** Violin plots depicting the number of genes (nFeature_RNA) and number of RNA counts detected per cell (nCount_RNA), the percent of detected genes which are mitochondrial genes (% mitochondrial) and number of ADT counts detected per cell (nCount_ADT). Horizontal lines indicate the QC thresholds for this patient. **C)** Violin plots visualising the QC metrics grouped by the identity assigned by de-multiplexing the hashtags.

Fig. S3 Durham et al.

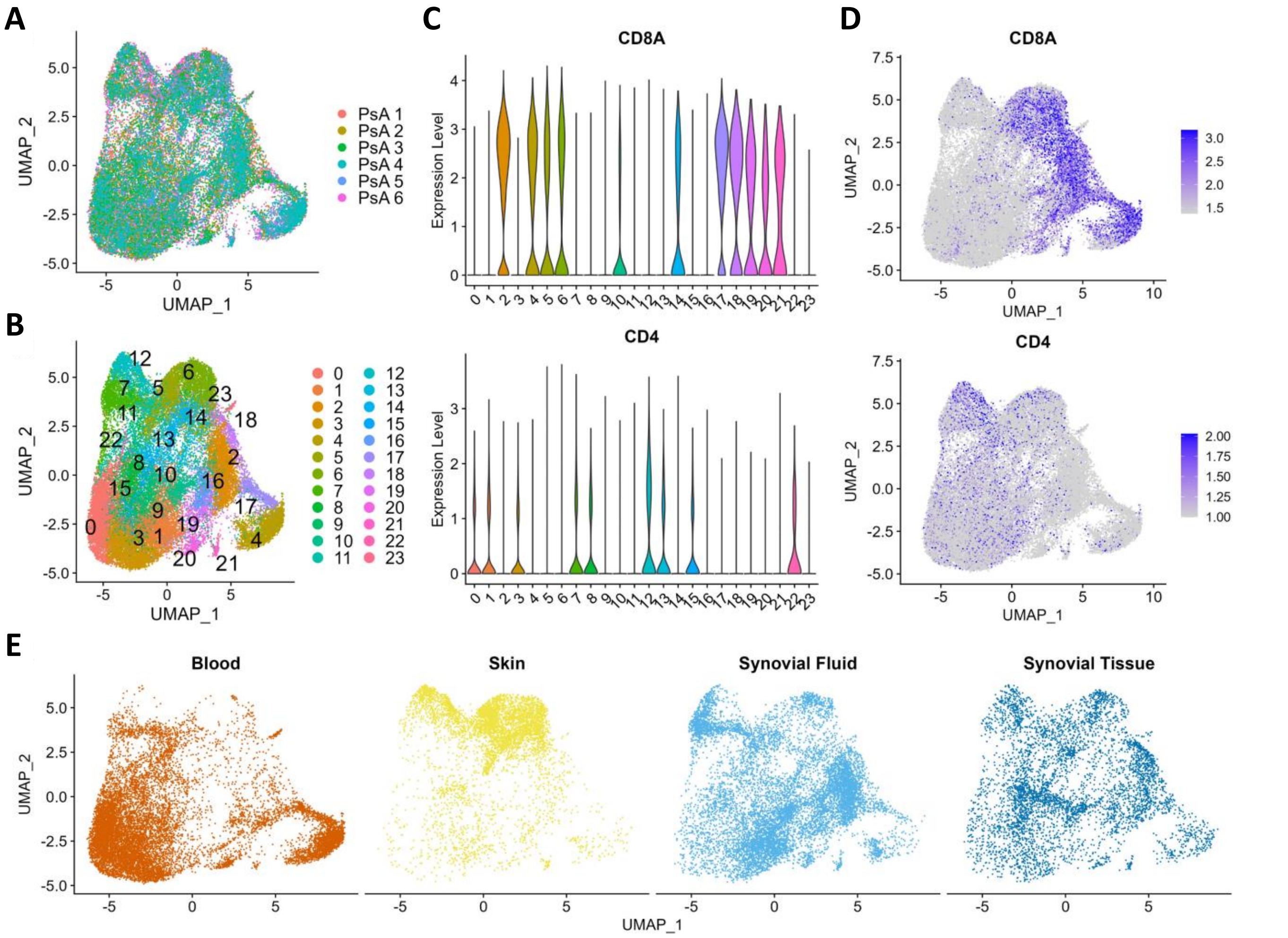

Figure S3: Integrated analysis of 35,491 memory T-cells from paired samples of blood, skin epidermis from inflamed psoriatic skin and synovial tissue and/or synovial fluid from inflamed knees from 6 patients with PsA.

A, B) UMAPs with cells coloured according to **(A)** patient and **(B)** the 24 cell populations obtained after Seurat clustering**. C)** Violin plots visualising *CD8A* and *CD4* expression within each cluster**. D)** UMAPs with cells coloured according to *CD8A* and *CD4* expression**. E)** UMAPs split by tissue of origin.

Fig. S4 Durham et al.

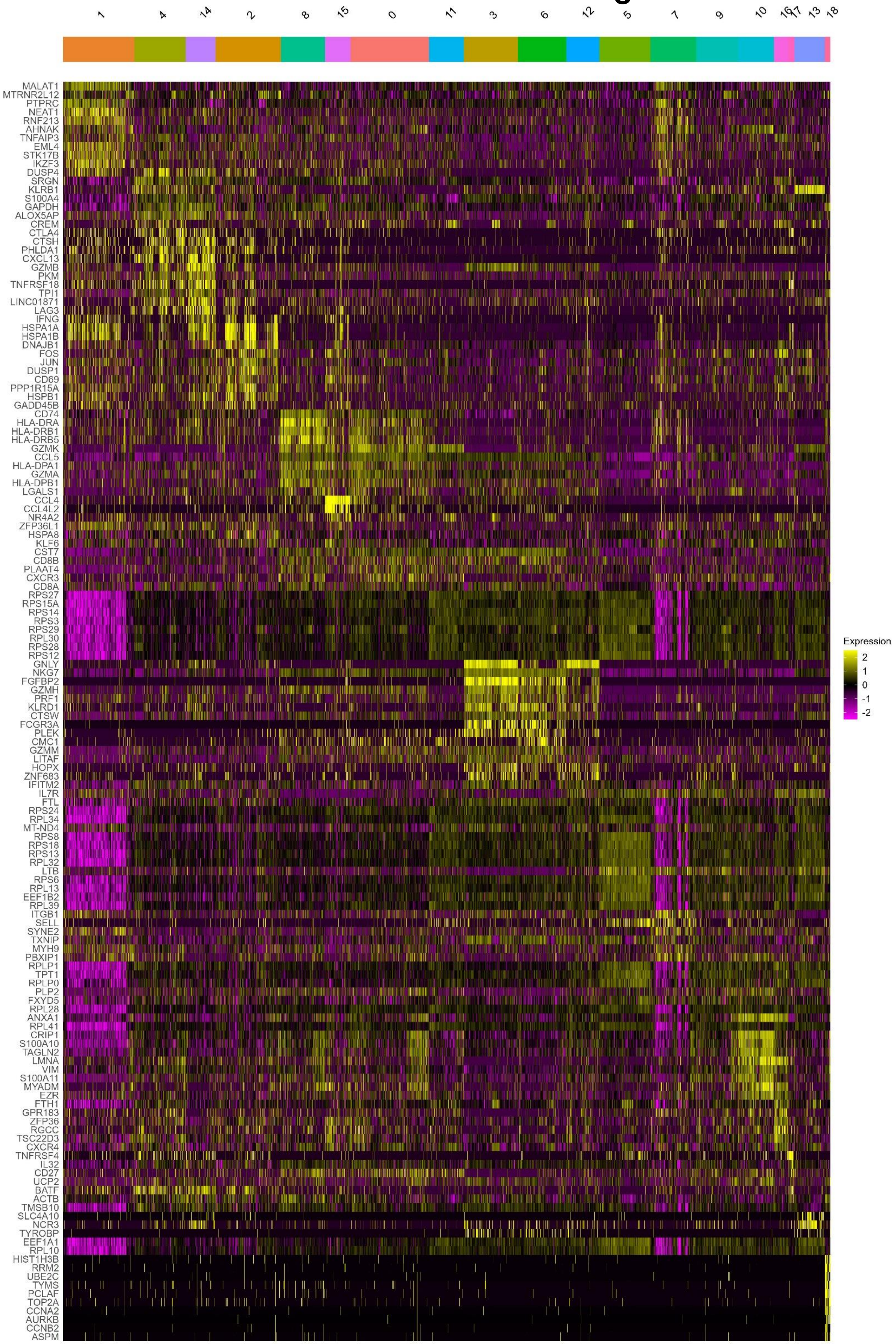

Figure S4: Heatmap visualising the top 10 genes differentially expressed by each of the 19 CD8+ T-cell clusters in the CD8+ T-cell only analysis. Clusters grouped into groups with similar phenotypes. Differential expression by SCTransformed RNA was calculated using the Wilcoxon signed rank test using the FindConservedMarkers() function Seurat. FindConservedMarkers() mitigates for potential batch effect between patients by performing differential gene expression testing for each patient separately and combining the p-values using meta-analysis methods from the MetaDE R package. Combined p value < 0.05 was used to identify significantly differentially expressed genes. Log2FC for each of the 6 patients was averaged and genes were ranked in order of average Log2FC change.

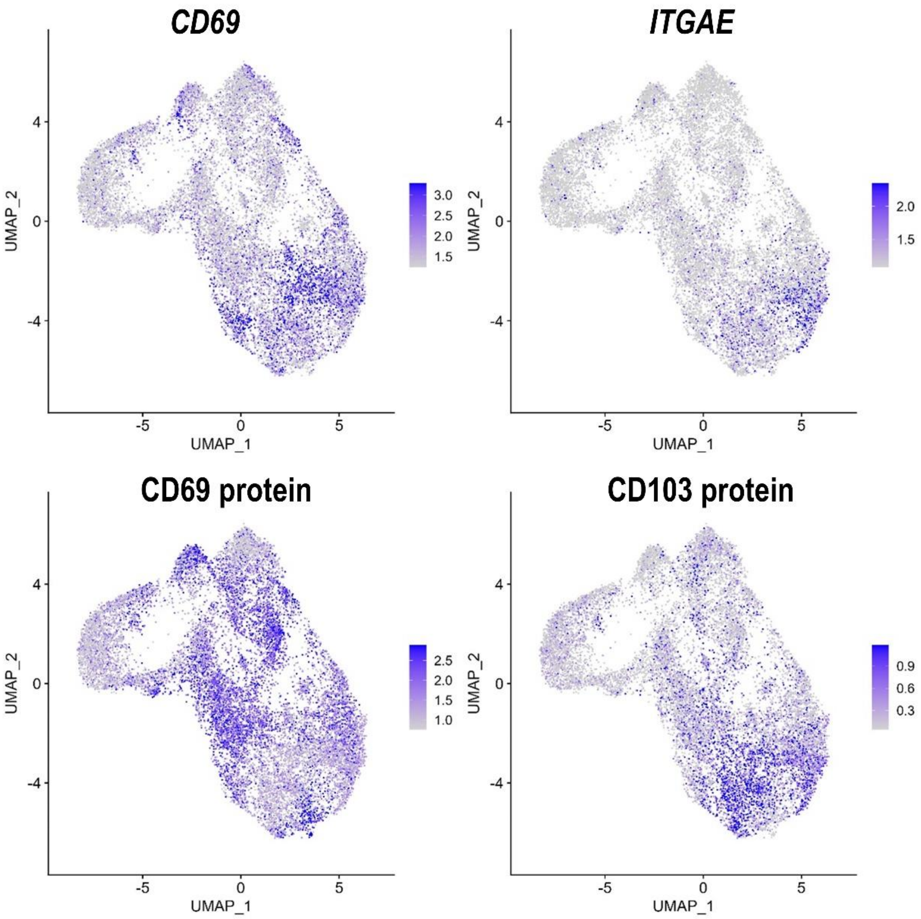

Figure S5: Expression of CD69 and CD103 by CD8+ T-cells from skin, ST, SF and blood.
UMAPs coloured by expression of *CD69* and *ITGAE* (encodes CD103) RNA (top row) and CD69 and CD103 protein (bottom row) (n=6 patients).

Fig. S6 Durham et al.

A

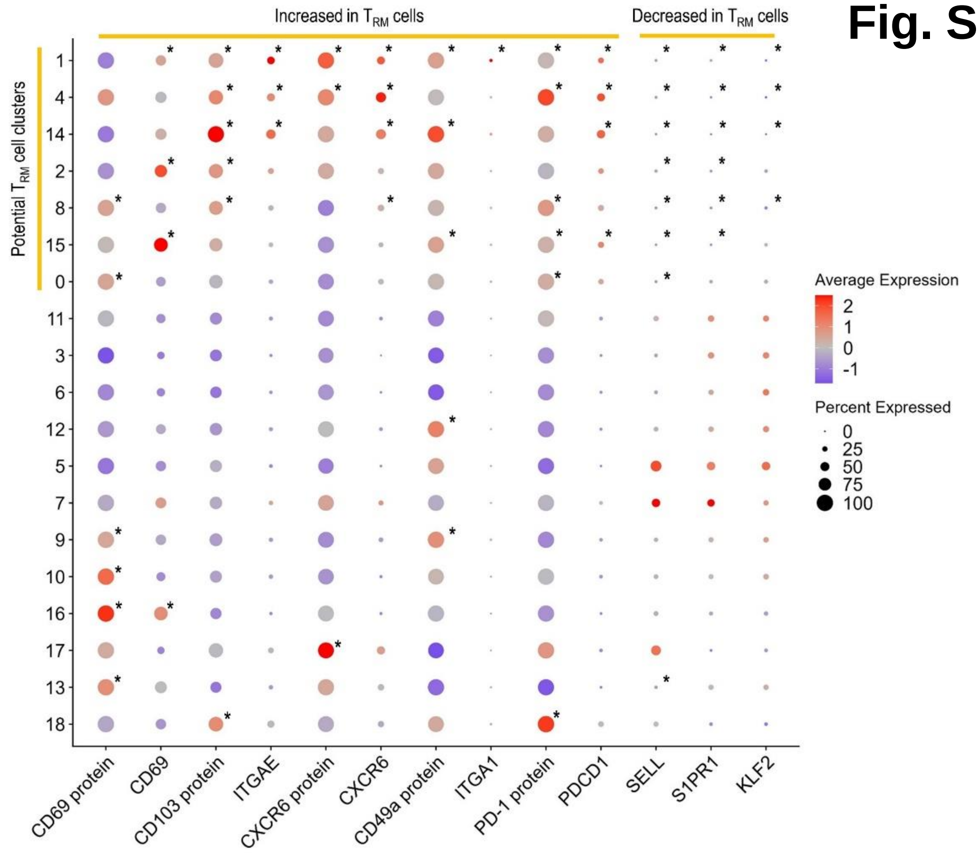

B

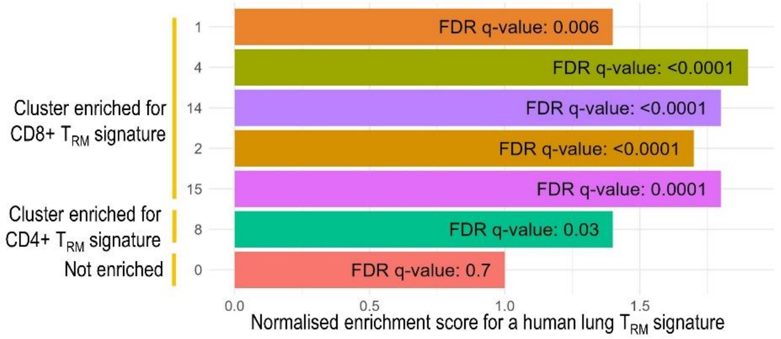

C

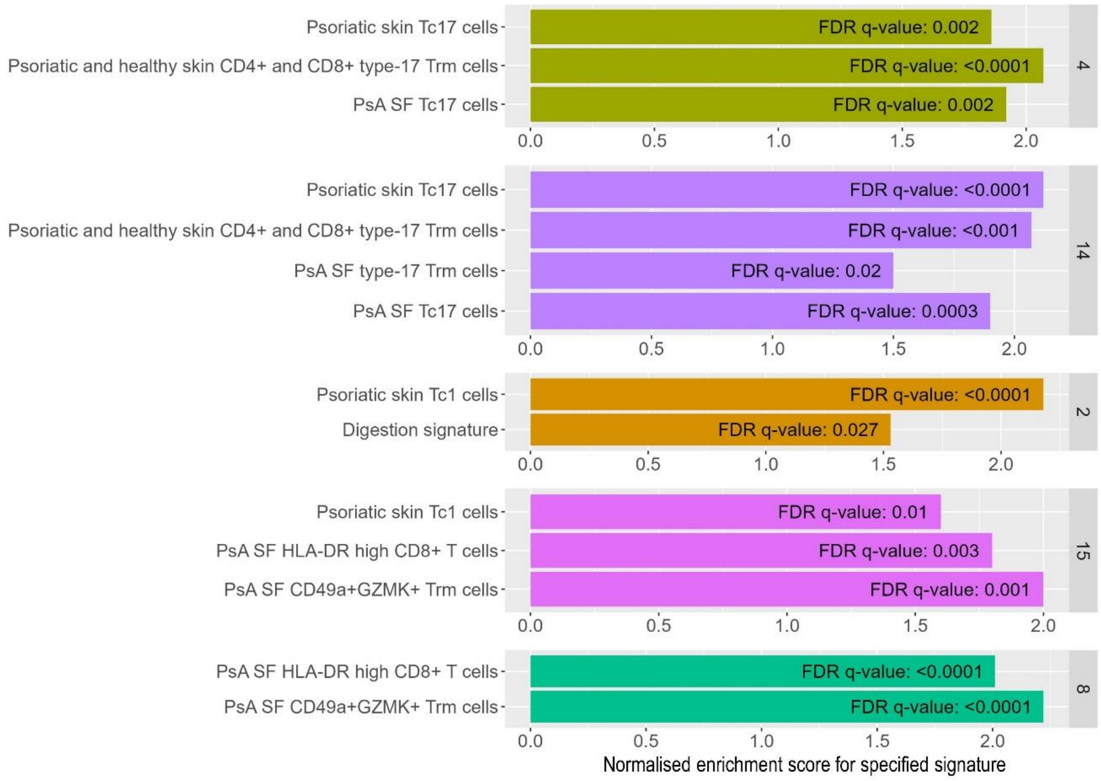

Figure S6: Identification and characterisation of CD8+ T_{RM} cell clusters.

A) Dot plot depicting the expression of specified genes/proteins across the CD8+ T-cell clusters. Size of dots indicates the % of cells within each cluster that express the indicated gene. Colour of dots indicates the scaled expression of indicated gene across the clusters. Horizontal yellow bars at the top of the dot plot indicate which genes/proteins are expected to be increased and which are expected to be decreased in T_{RM} cells[1]. For each cluster, genes/proteins which are significantly differentially expressed in the direction expected for T_{RM} cells are marked with an asterisk. Vertical yellow bar indicates clusters meeting criteria to be defined as “potential T_{RM} cell clusters”. Differential expression of SCTransformed RNA for each cluster compared to all other cells was calculated using the Wilcoxon signed rank test using the FindAllMarkers() function in Seurat. Adjusted p value <0.05 considered significant. **B)** Results (normalised enrichment score and FDR q-values) of GSEA indicating positive enrichment of human lung T_{RM} cell signature[1] in clusters 1, 2, 4, 8, 14 and 15 when compared to pooled cells from non-potential T_{RM} clusters (clusters 3, 5, 6, 7, 9, 10, 11, 12, 13, 16, 17 and 18). Cluster 0 was not enriched for any of the tested T_{RM} signatures (human lung and spleen CD4+ and CD8+ T_{RM} signatures[1], normalised enrichment score visualised for cluster 0 is for human lung CD8+ T_{RM} signature). (n=6 patients). **C)** Results (normalised enrichment score and FDR q-values) of GSEA analysis for each T_{RM} cluster compared to pooled cells from other T_{RM} clusters. Cluster 1 was not positively enriched for any of the tested gene sets compared to other T_{RM} clusters therefore is not depicted here. However, cluster 1 had significant upregulation of RORA and CCR6 and CD161 protein and therefore was classified as having a type-17 phenotype.

Note: Cluster 0 met the criteria of a “potential-T_{RM} cluster” but was not positively enriched for a T_{RM} signature on GSEA (Figure S6B). It contained predominantly synovial cells (Main Figure 1C) that expressed high levels of GZMK and HLA-DR genes (Main Figure 1D, Supplementary Data 2), similar to SF CD49a+GZMK+ T_{RM} cells in PsA[7]. Cluster 0 shared high expression of GZMK with cluster 11 and therefore was classified as “GZMK+” (Figure 1D). We speculate that cells in cluster 0 may be transitioning from a GZMK+ phenotype shared with blood (cluster 11) towards a GZMK+ T_{RM} phenotype (cluster 8 and 15).

References for gene lists used for GSEA: Human lung and spleen CD4+ and CD8+ T_{RM} signatures[1], Psoriatic skin Tc17 cells[2], Psoriatic and healthy skin CD4+ and CD8+ type-17 T_{RM} cells[3], PsA SF Tc17 cells[4], PsA SF Type-17 T_{RM} cells[5], Psoriatic skin Tc1 cells[2], Digestion signature[6], PsA SF HLA-DR high CD8+ T-cells[7], PsA SF CD49a+ GZMK+ T_{RM} cells[5].

1. Kumar, B. V., Ma, W., Miron, M., et al. Human tissue-resident memory T cells are defined by core transcriptional and functional signatures in lymphoid and mucosal sites. Cell Rep 20, 2921–2934 (2017).
2. Liu, J., Chang, H. W., Huang, Z. M., et al. Single-cell RNA sequencing of psoriatic skin identifies pathogenic Tc17 cell subsets and reveals distinctions between CD8+ T cells in autoimmunity and cancer. Journal of Allergy and Clinical Immunology 147, 2370–2380 (2021).
3. Cook, C. P., Taylor, M., Liu, Y., et al. A single-cell transcriptional gradient in human cutaneous memory T cells restricts Th17/Tc17 identity. Cell Rep Med 3, 100715 (2022).
4. Steel, K. J. A., Srenathan, U., Ridley, M., et al. Polyfunctional, proinflammatory, tissue-resident memory phenotype and function of synovial interleukin-17A+CD8+ T cells in psoriatic arthritis. Arthritis and Rheumatology 72, 435–447 (2020).
5. Povoleri, G., Durham, L. E., Gray, E. H., et al. Psoriatic and rheumatoid arthritis joints differ in the composition of CD8+ tissue-resident memory T-cell subsets. Cell Rep 42, 112514 (2023).
6. O’Flanagan, C. H., Campbell, K. R., Zhang, A. W., et al. Dissociation of solid tumor tissues with cold active protease for single-cell RNA-seq minimizes conserved collagenase-associated stress responses. Genome Biol 20, 210 (2019).
7. Penkava, F., Velasco-Herrera, M. D. C., Young, M. D., et al. Single-cell sequencing reveals clonal expansions of pro-inflammatory synovial CD8 T cells expressing tissue-homing receptors in psoriatic arthritis. Nat Commun 11, 4767 (2020).

Fig. S7 Durham et al.

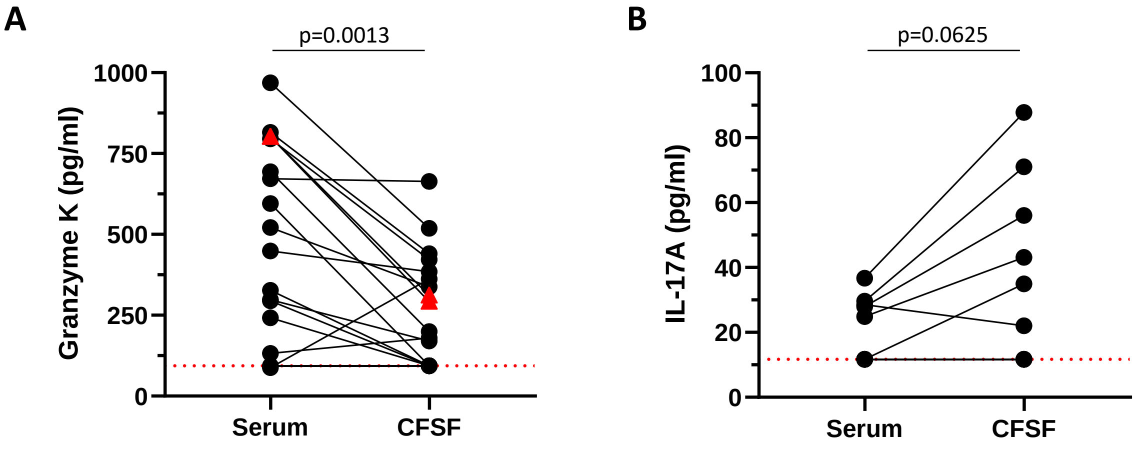

Figure S7: Presence of granzyme K and IL-17A in serum and cell-free synovial fluid in PsA.
A,B) Levels of **(A)** granzyme K (n=17) and **(B)** IL-17A (n=10) in paired serum and cell-free SF (CFSF) from patients with PsA measured by ELISA. Red triangles indicate granzyme K in CFSF obtained from left and right knees from the same patient. Wilcoxon matched pairs signed rank test (two-tailed). Red dashed line indicated the lowest detection limit. Values below the lowest detection limit (n=5 serum, n=4 CFSF) were set to this value.

Fig. S8 Durham et al.

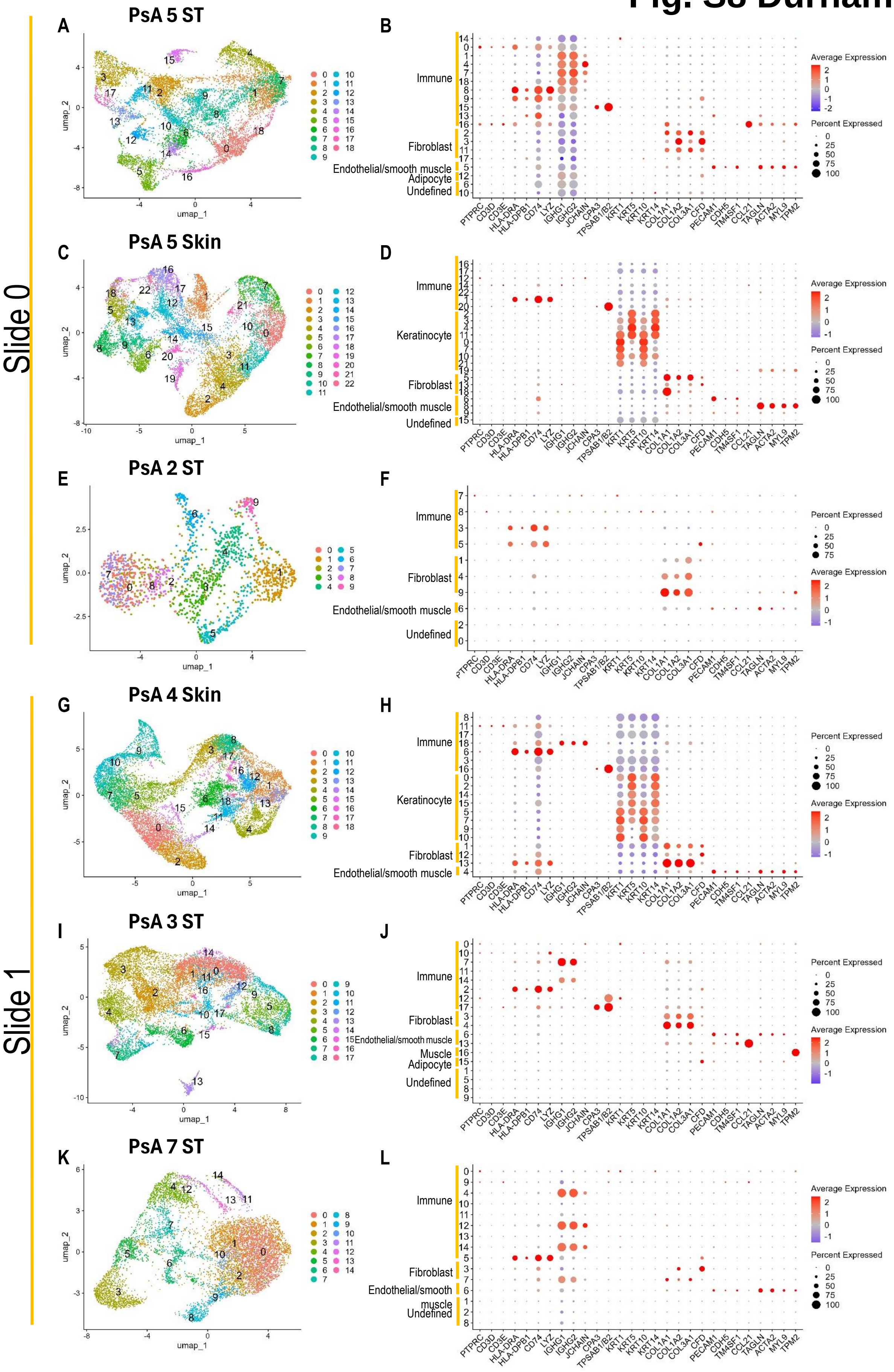

Figure S8: spatial transcriptomics cell type annotation.
Each tissue section was analysed independently to identify cell types. **A, C, E, G, I, K)** UMAPs of independent analysis of each individual tissue section, cells coloured according to cluster. **B, D, F, H, J, L)** Dot plots showing expression of canonical marker genes across clusters. Size of dots indicates the % of cells within the cluster that express the indicated gene. Colour of dots indicates the scaled expression of indicated gene across the clusters. Yellow bars indicate the annotation of each cluster.

Fig. S9 Durham et al.

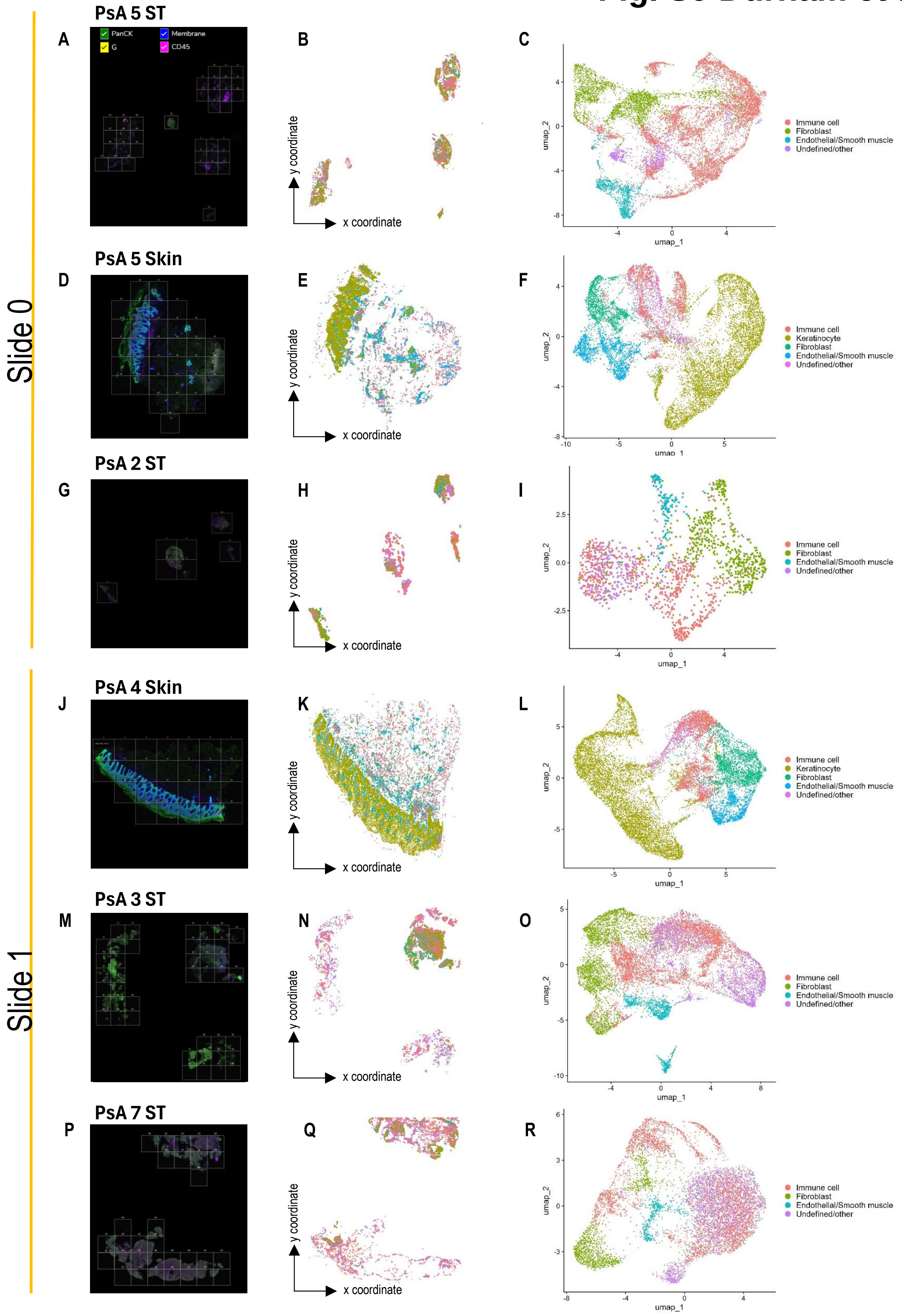

Figure S9: spatial transcriptomics cell type annotation.
A, D, G, J, M, P) Images of tissue sections used for spatial transcriptomics imaged on CosMx analyser. Grid lines indicate 0.51x0.51mm fields of view selected for data acquisition. Corresponding tissue sections **(B, E, H, K, N, Q)** and UMAPs **(C, F, I, L, O, R)** with cells coloured according to cell type annotation as defined in **Figure S8**.
Note panel D is duplicated from Figure 3, it is included in this figure for clarity.

Fig. S10 Durham et al.

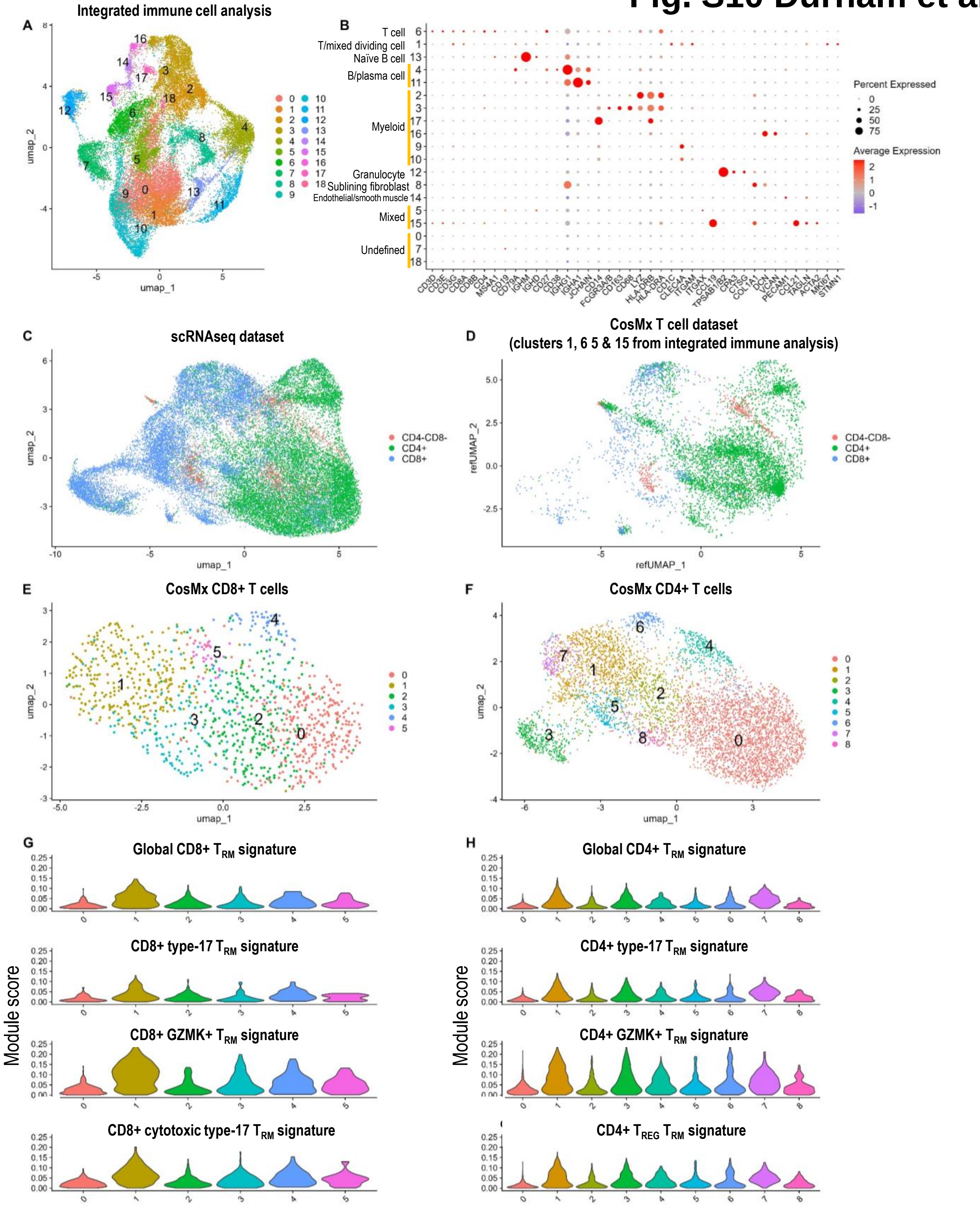

Figure S10: integrated immune cell analysis and identification of T_{RM} cells.

A) Integrated analysis of immune cells from sections of skin (n=2) and ST (n=4) from patients with PsA. **B)** Dot plot showing expression of canonical marker genes across clusters. Size of dots indicates the %cells within the cluster that express the indicated gene. Colour of dots indicates the scaled expression of indicated gene across the clusters. Labels to left of cluster number indicate the annotation of each cluster. **C, D)** Mapping CosMx T-cell dataset onto scRNAseq T-cell dataset. **C)** Integrated scRNAseq analysis of 35,491 T-cells from skin, joint and blood from patients with PsA, cells coloured according to CD4+/CD8+ T-cell status. **D)** UMAP of integrated analysis of clusters 1, 5, 6 and 15 (potential T-cell clusters comprising 7,449 cells) from integrated immune cell analysis mapped onto the scRNAseq dataset UMAP. Cells coloured according to predicted CD4+/CD8+ T-cell status. **E - H)** Identification of CD8+ and CD4+ T_{RM} cells. UMAPs of integrated analysis of **E)** 1,155 CD8+ T-cells and **F)** 5,662 CD4+ T-cells. Cells coloured according to Seurat cluster. **G, H)** Violin plots depicting module score for CD8+/CD4+ T_{RM} cell subset signatures from the scRNAseq analysis across clusters in the **G)** CD8+ and **H)** CD4+ T-cell CosMx analysis.

Fig. S11 Durham et al.

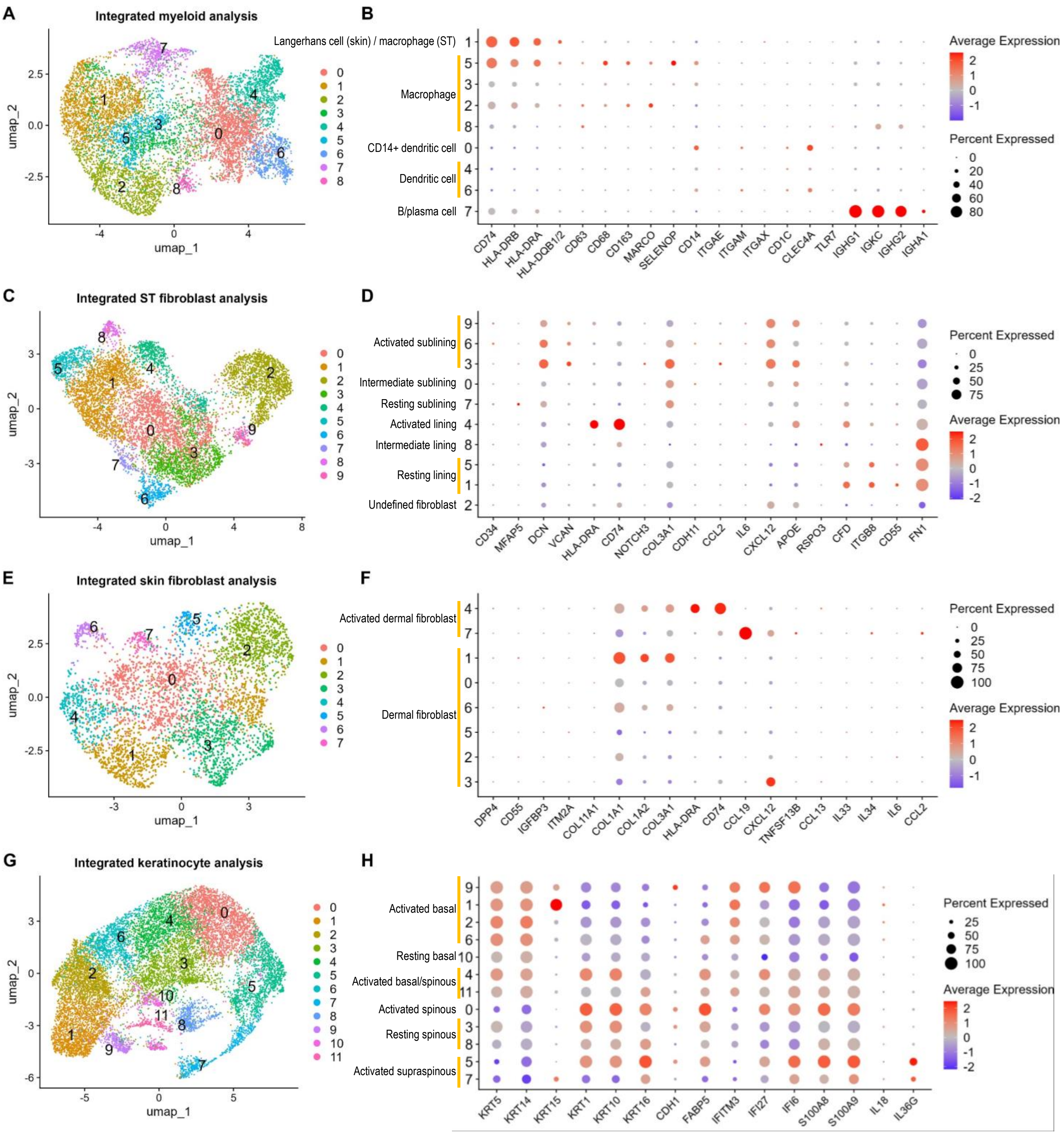

Figure S11: Integrated analysis of myeloid cells, fibroblasts and keratinocytes.
A, C, E, G) Integrated analysis of **A)** myeloid cells (n=2 skin, n=4 ST), **C)** ST fibroblasts (n=4 ST), **E)** skin fibroblasts (n=2 skin) and **G)** keratinocytes (n=2 skin) from tissue sections from patients with PsA. **B, D, F, H)** Dot plot showing expression of canonical marker genes across clusters in integrated analysis of **B)** myeloid cells, **D)** ST fibroblasts, **F)** skin fibroblasts and **H)** keratinocytes. Size of dots indicates the % of cells within the cluster that express the indicated gene. Colour of dots indicates the scaled expression of indicated gene across the clusters. Labels to left of cluster numbers indicate the annotation of each cluster.

Fig. S12 Durham et al.

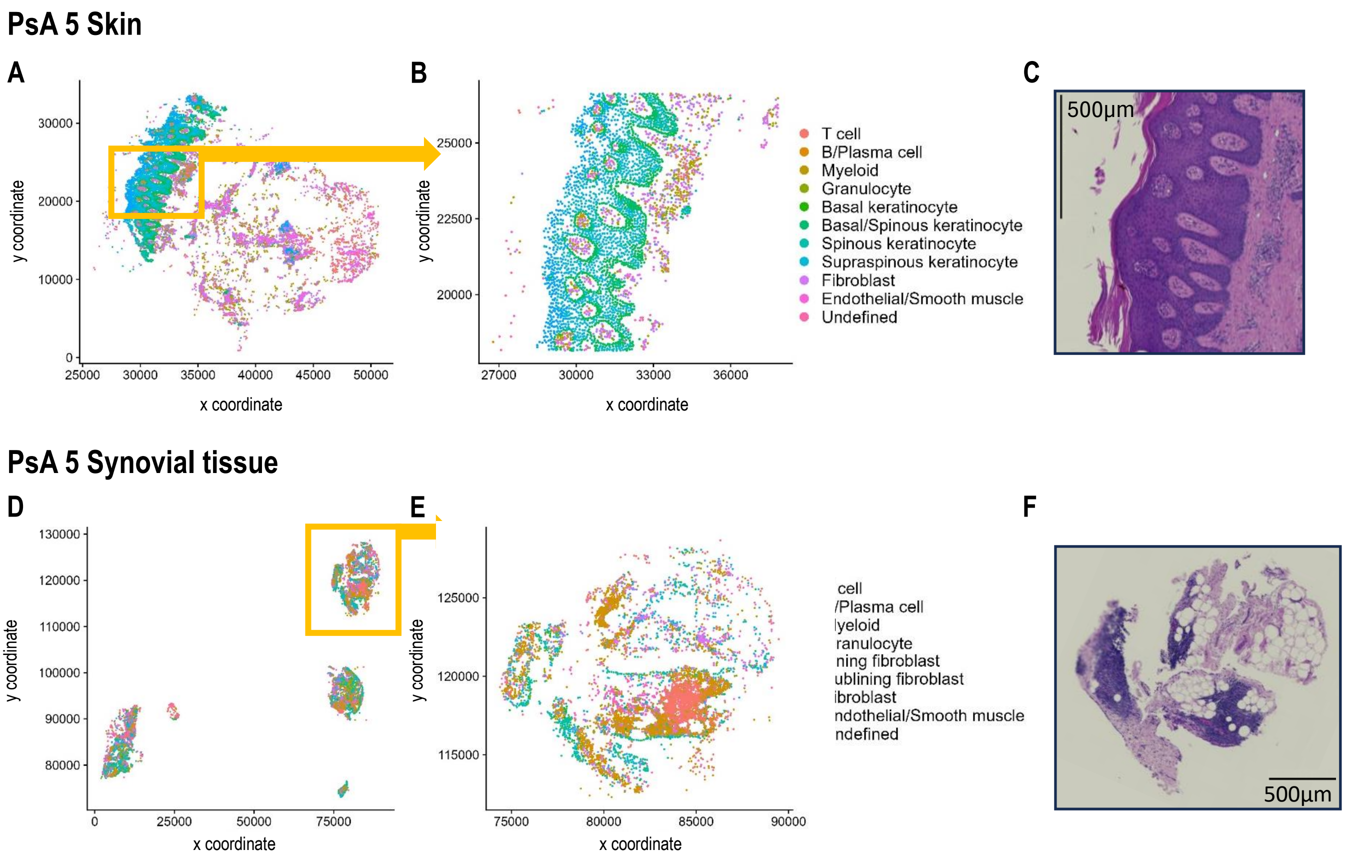

Figure S12: Spatial location of immune cell subsets in skin and joint in PsA.
Tissue sections from paired biopsies of skin **(A-C)** and ST **(D-F)** from a patient with PsA (PsA 5) analysed using CosMx with cells coloured according to cell type **(A, B, D, E)** and H&E staining **(C, F)**. **B** and **E** are close ups of tissue sections depicted in panels **A** and **D,** respectively. Note that the cell annotation which was based upon gene expression in the CosMx analysis corresponds with the tissue architecture of the sequential tissue sections stained with H&E (for example, keratinocytes are located in the epidermis in the skin section).

Fig. S13 Durham et al.

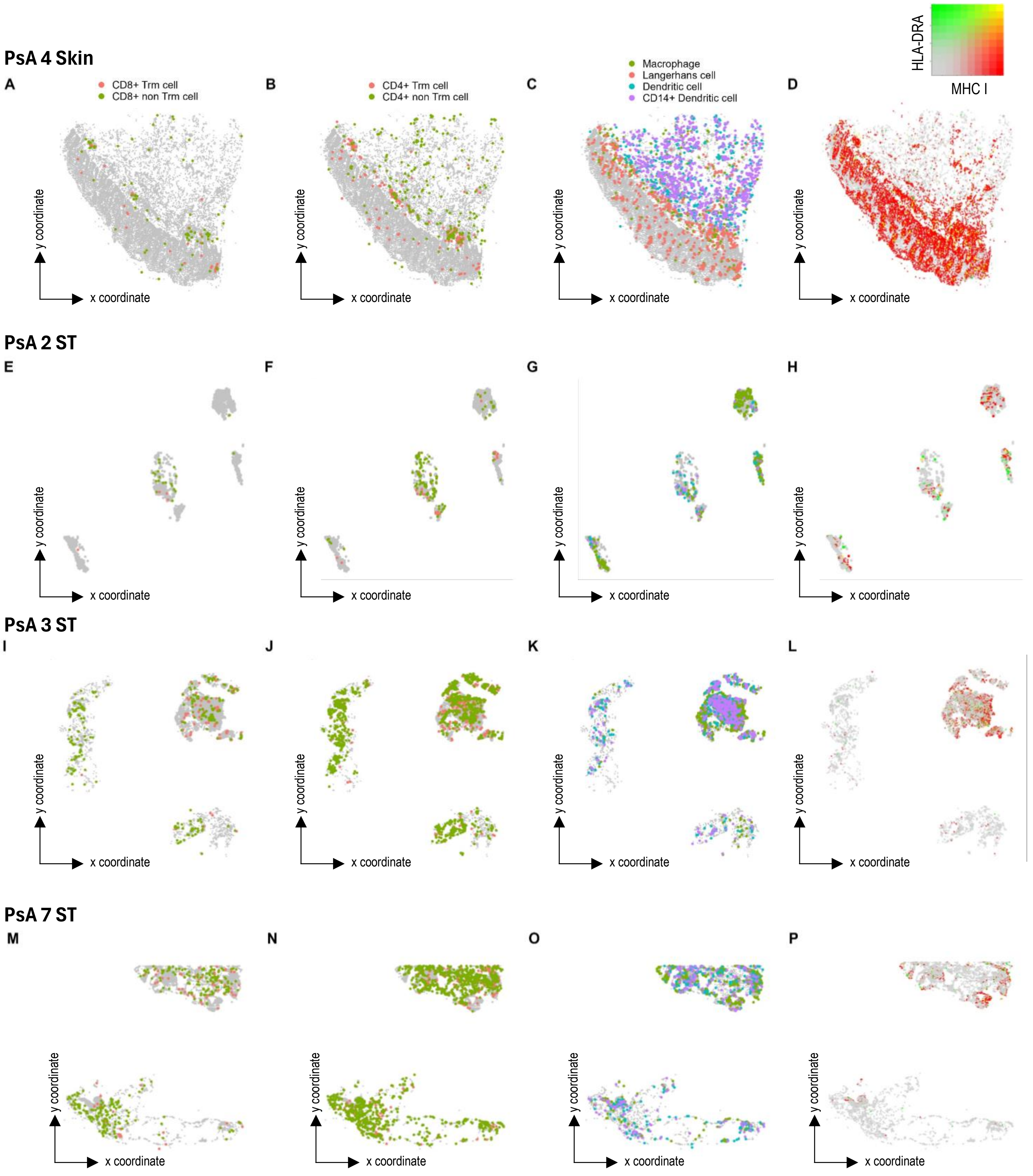

Figure S13: Spatial location of immune cell subsets in skin and synovial tissue.
Figures show spatial location of cell subsets for skin from PsA 4 **(A-D)** and ST from patients PsA 2 **(E-H)**, PsA 3 **(I-L)** and PsA 7 **(M-P)**. Spatial location of (**A, E, I, M)** CD8+ and **(B, F, J, N)** CD4+ T_{RM} and non-T_{RM} cells, **(C, G, K, O)** myeloid cell subsets and **(D, H, L, P)** MHC class I and class II expression in tissue sections.

Fig. S14 Durham et al.

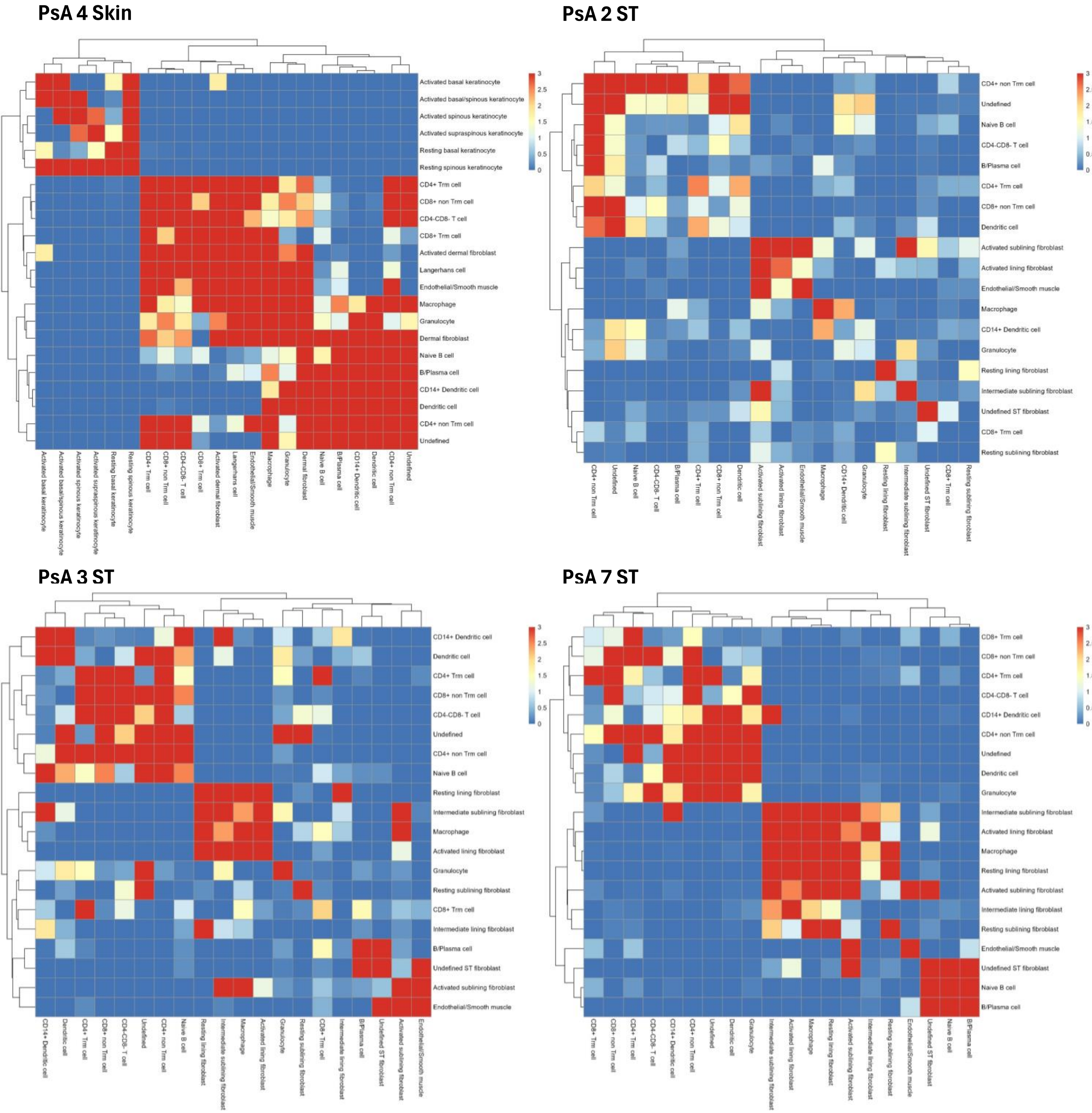

Figure S14: Neighbourhood analysis in inflamed skin and joints in PsA. Heatmaps depicting significance of contact-based interactions between cell types in skin from patient PsA 4 and ST from patients PsA 2, PsA 3 and PsA 7. Colour of square indicates the -Log$_{10}$p value (upper tail, one-sided) for whether cell types are more commonly neighbours than expected by chance.

Fig. S15 Durham et al.

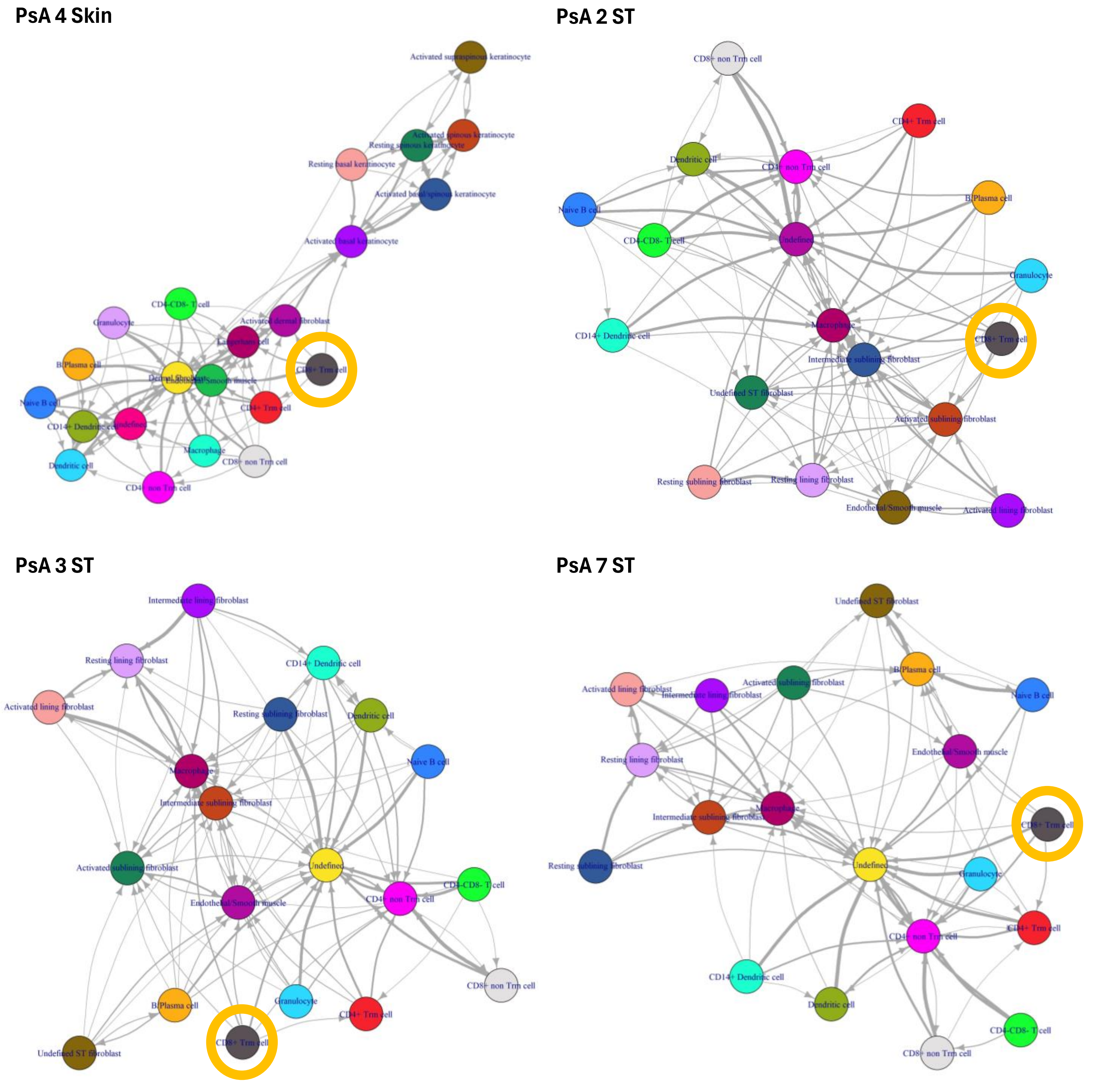

Figure S15: Neighbourhood analysis in inflamed skin and joints in PsA. Force directed graphs depicting contact-based interactions between cell type in skin from patient PsA 4 and ST from patients PsA 2, PsA 3 and PsA 7. Arrows between two cell types indicate that neighbourhoods of the cell type that the arrow originates from are composed of ≥5% of the cell type that the arrow points to. Yellow circles highlight the nodes corresponding to CD8+ T_{RM} cells.

A PsA 5 Skin CD8+ T_{RM} cell – macrophage interactions

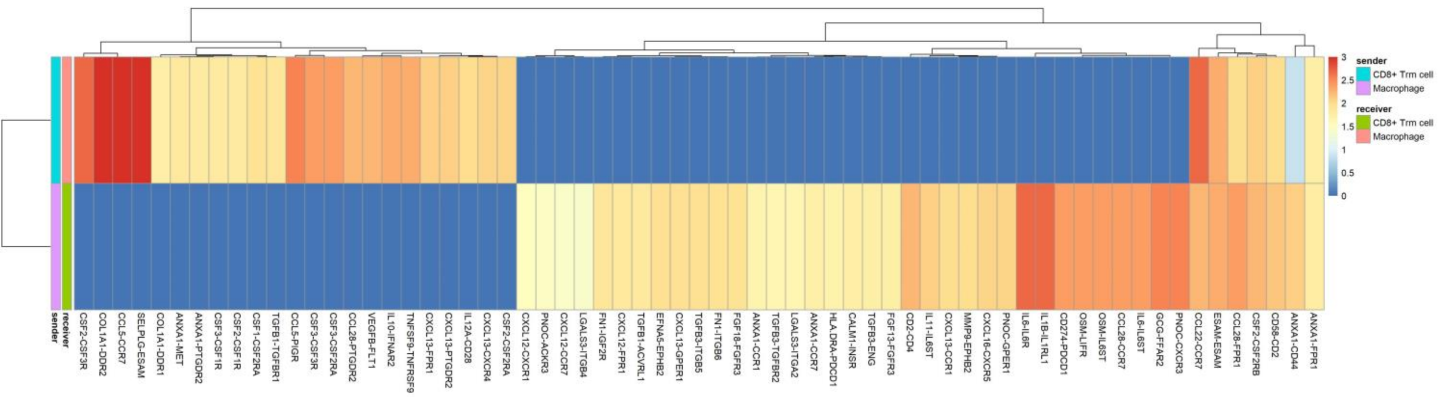

B PsA 5 Skin CD8+ T_{RM} cell – Langerhans cell interactions

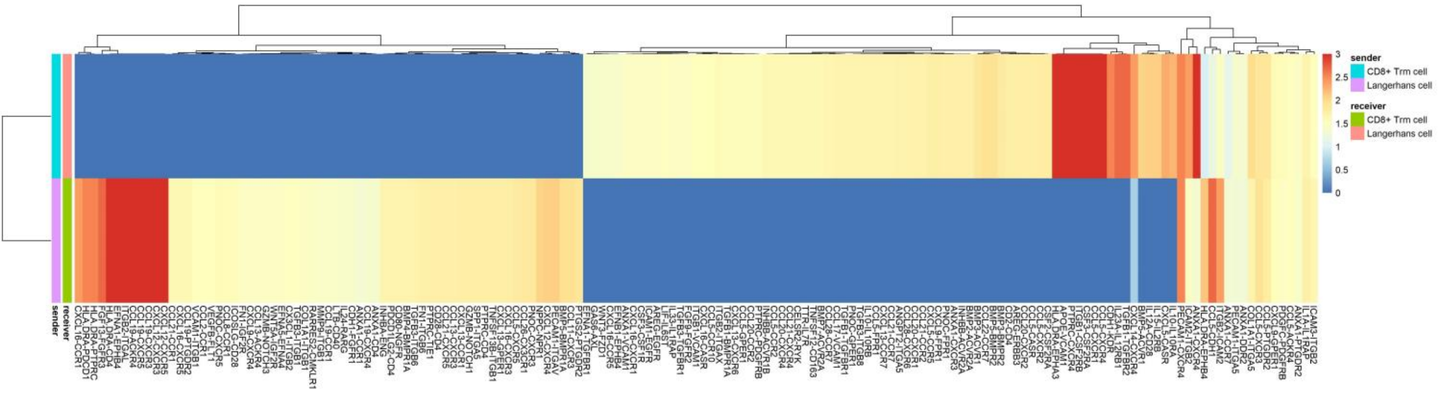

C PsA 4 Skin CD8+ T_{RM} cell – macrophage interactions

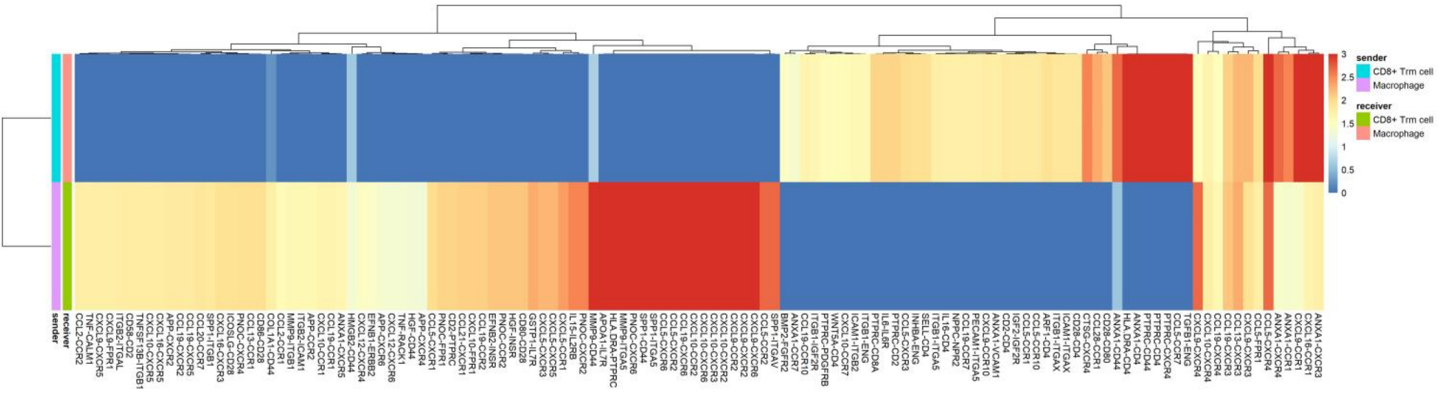

D PsA 4 Skin CD8+ T_{RM} cell – Langerhans cell interactions

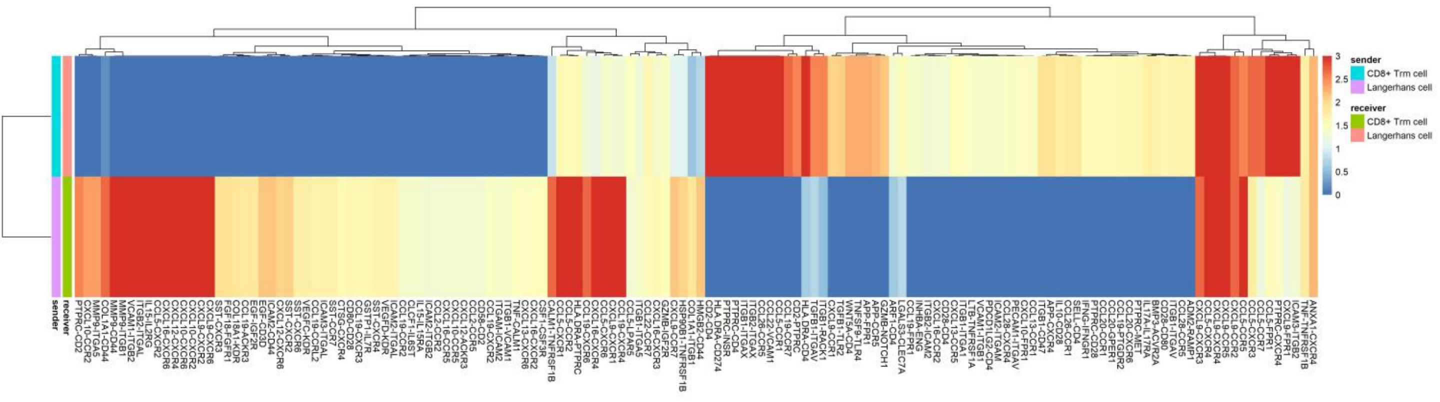

Figure S16: Ligand-receptor interactions between CD8+ T_{RM} cells and antigen-presenting cells in skin A-D) Significantly enriched ligand-receptor interactions between macrophages and CD8+ T_{RM} cells, and Langerhans cells and CD8+ T_{RM} cells. **(A)** CD8+ T_{RM} cells and macrophages in PsA 5 skin, **(B)** CD8+ T_{RM} cells and Langerhans cells in PsA 5 skin, **(C)** CD8+ T_{RM} cells and macrophages in PsA 4 skin, **(D)** CD8+ T_{RM} cells and Langerhans cells in PsA 4 skin.

Fig. S17 Durham et al.

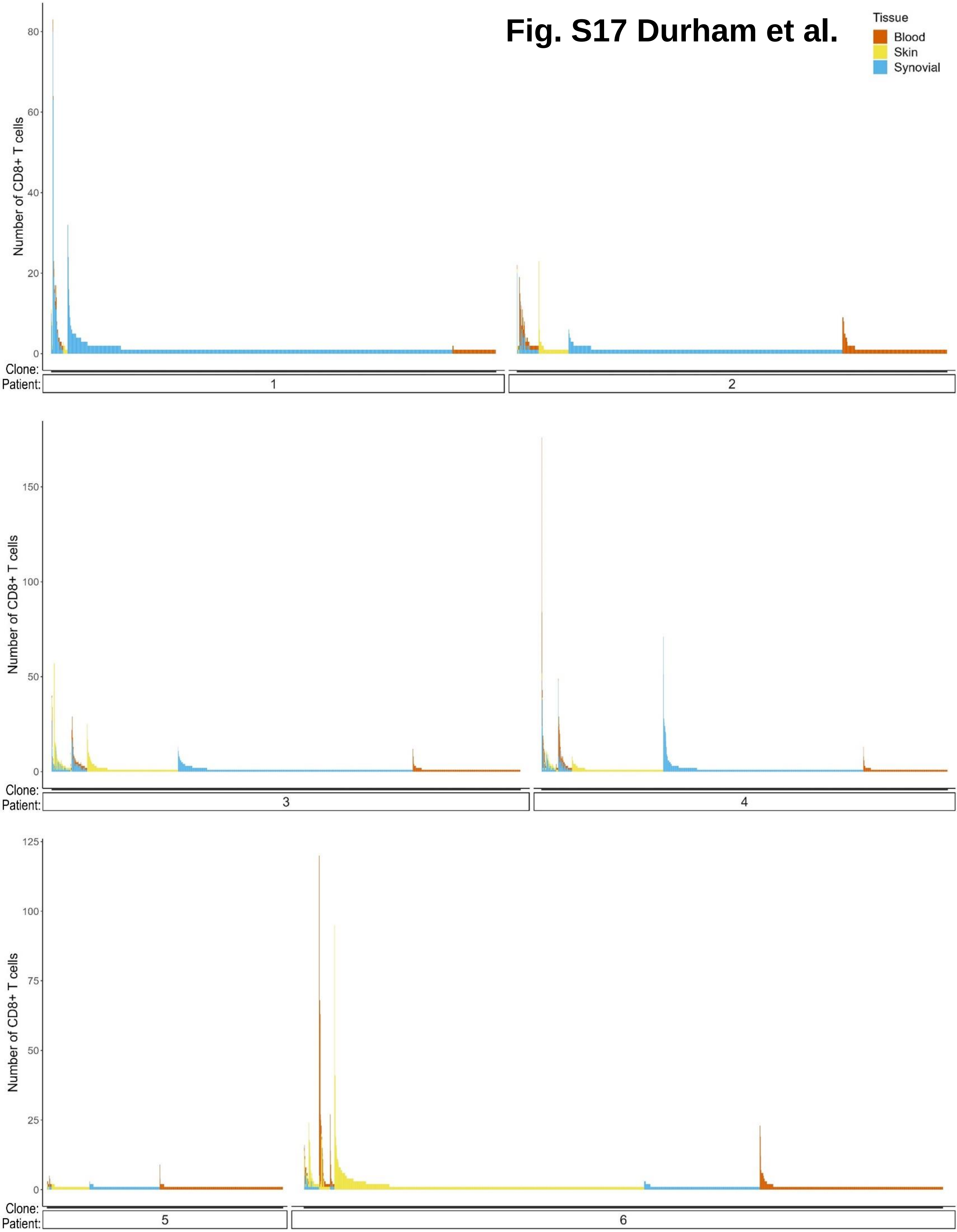

Figure S17: Tissue location of all CD8+ T-cell clones.

Bar chart visualising the size and tissue location of all CD8+ T-cell clones. Each vertical bar represents a CD8+ T-cell clone. The height of the bar represents the number of CD8+ T-cells within that clone and the colour represents the tissue that the cells were detected in (red = blood, yellow = skin, blue = joint). Clones are split by patient. Within each patient clones are ordered into triple shared clones, followed by dual shared clones (clones that were detected in the skin and the joint, in the joint and blood and in the skin and blood) followed by clones that were only detected in a single tissue (skin, joint or blood).

Fig. S18 Durham et al.

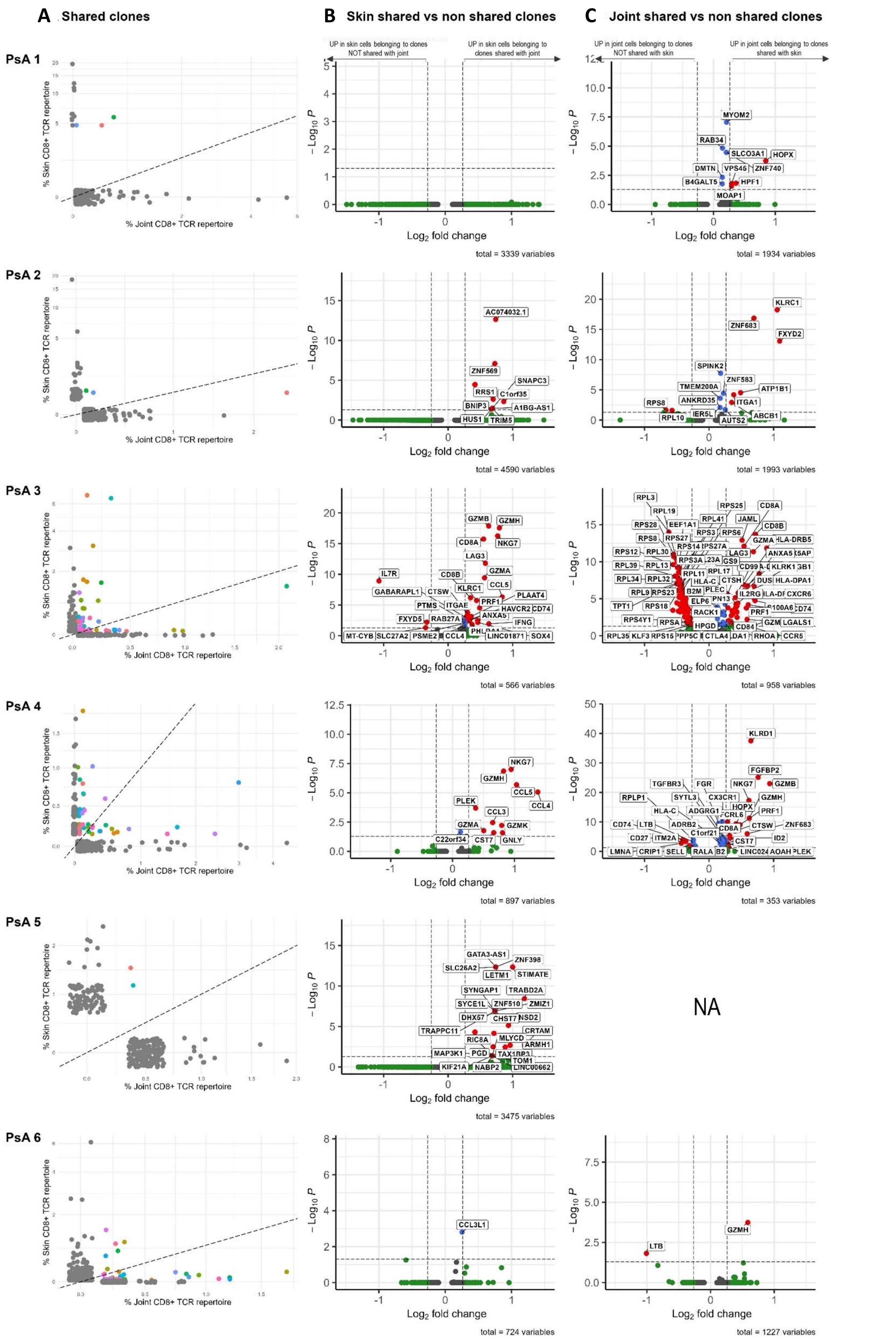

Figure S18: CD8+ T-cell clones shared between skin and synovium

A) Scatterplots for each patient showing the frequency of CD8+ T-cell clones in skin and joint. Each dot represents a CD8+ T-cell clone and the position of the dot along the x and y axes indicates the frequency of that clone in the joint and skin respectively. Scatterplot for PsA 3 is duplicated from Figure 5A for clarity. **B)** Volcano plot showing significantly differentially expressed genes between skin CD8+ T cells that belong to skin-joint shared clones vs. non-shared clones for each individual patient. **C)** Volcano plot showing differentially expressed genes between joint CD8+ T cells that belong to skin-joint shared clones vs. non-shared clones for each individual patient. Differential genes calculated using FindMarkers() in Seurat. NB: Differentially expressed genes not calculated for PsA 5 joint due to insufficient number of cells belonging to shared clones in the joint (n < 3).

Fig. S19 Durham et al.

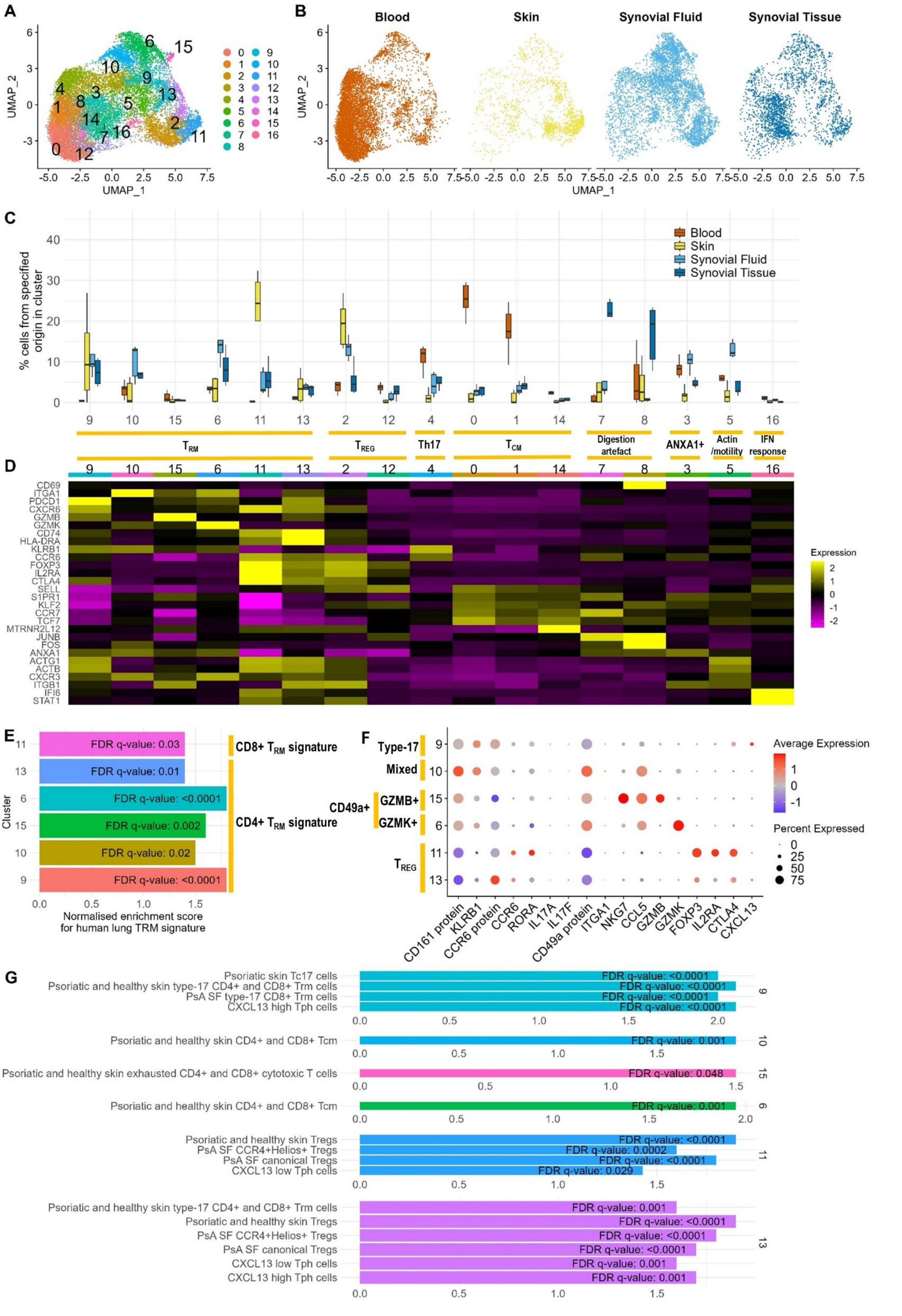

Figure S19: Integrated analysis of 17,700 memory CD4+ T-cells from paired samples of blood, lesional skin epidermis and ST and/or SF from inflamed knees from 6 patients with PsA.

A) UMAP with cells coloured according to the 17 CD4+ T-cell populations obtained after Seurat clustering. **B)** UMAPs split by tissue of origin. **C)** Boxplot visualising the distribution of CD4+ T-cells from each tissue across the 17 clusters. Clusters are grouped into groups with similar phenotypes. **D)** Heatmap visualising expression of specified genes across the clusters. **E)** Results (normalised enrichment score and FDR q-values) of gene set enrichment analysis showing positive enrichment of human lung T_{RM} cell signature[1] in clusters 6, 9, 10, 11, 13 and 15 when compared to pooled cells from non-potential-T_{RM} clusters. **F)** Dot plot depicting expression of select genes across the CD4+ T_{RM} clusters. Size of dots indicates the % of cells within each cluster that express the indicated gene. Colour of dots indicates the scaled expression of indicated gene across all of the CD4+ T_{RM} clusters. Yellow bars indicate clusters with similar phenotypes. **G)** Results of gene set enrichment analysis for each T_{RM} cluster compared to pooled cells from other T_{RM} clusters.

References for gene lists used for GSEA: Human lung and spleen CD4+ and CD8+ T_{RM} signatures[1], Psoriatic skin Tc17 cells[2], Psoriatic and healthy skin CD4+ and CD8+ type-17 T_{RM} cells[3], PsA SF type-17 CD8+ T_{RM} cells[4], CXCL13 high T_{PH} cells[5], Psoriatic and healthy skin CD4+ and CD8+ T_{CM} cells[3], Psoriatic and healthy skin exhausted CD4+ and CD8+ cytotoxic T cells[3], Psoriatic and healthy skin T_{REG}s[3], PsA SF CCR4+Helios+ T_{REG}s[6], PsA SF canonical T_{REG}s[6], CXCL13 low T_{PH} cells[5].

1. Kumar, B. V., Ma, W., Miron, M., et al. Human tissue-resident memory T cells are defined by core transcriptional and functional signatures in lymphoid and mucosal sites. Cell Rep 20, 2921–2934 (2017).
2. Liu, J., Chang, H. W., Huang, Z. M., et al. Single-cell RNA sequencing of psoriatic skin identifies pathogenic Tc17 cell subsets and reveals distinctions between CD8+ T cells in autoimmunity and cancer. Journal of Allergy and Clinical Immunology 147, 2370–2380 (2021).
3. Cook, C. P., Taylor, M., Liu, Y., et al. A single-cell transcriptional gradient in human cutaneous memory T cells restricts Th17/Tc17 identity. Cell Rep Med 3, 100715 (2022).
4. Povoleri, G., Durham, L. E., Gray, E. H., et al. Psoriatic and rheumatoid arthritis joints differ in the composition of CD8+ tissue-resident memory T-cell subsets. Cell Rep 42, 112514 (2023).
5. Argyriou, A., Wadsworth, M. H., Lendvai, A., et al. Single cell sequencing identifies clonally expanded synovial CD4+ TPH cells expressing GPR56 in rheumatoid arthritis. Nat Commun 13, 4046 (2022).
6. Simone, D., Penkava, F., Ridley, A., et al. Single cell analysis of spondyloarthritis regulatory T cells identifies distinct synovial gene expression patterns and clonal fates. Commun Biol 4, 1395 (2021).

Fig. S20 Durham et al.

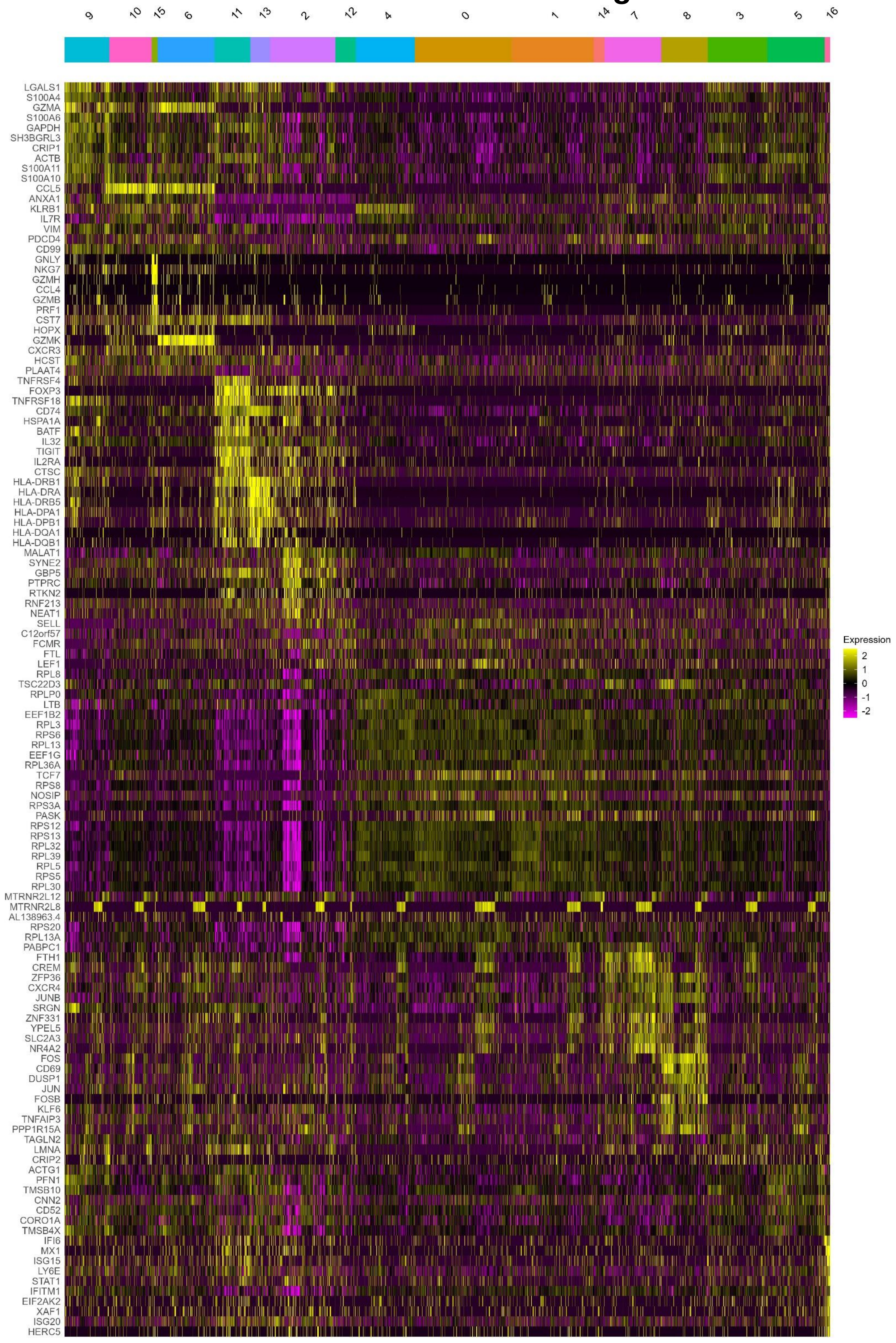

Figure S20: Heatmap visualising the top 10 genes differentially expressed by each of the 17 CD4+ T-cell clusters in the CD4+ T cell analysis. Clusters grouped into groups with similar phenotypes. Differential expression by SCTransformed RNA was calculated using the Wilcoxon signed rank test using the FindConservedMarkers() function Seurat. FindConservedMarkers() mitigates for potential batch effect between patients by performing differential gene expression testing for each patient separately and combining the p-values using meta-analysis methods from the MetaDE R package. Combined p value < 0.05 was used to identify significantly differentially expressed genes. Log2FC for each of the 6 patients was then averaged and genes were then ranked in order of average Log2FC change.
