# Supplementary material for: Linking Skin and Joint Inflammation in Psoriatic Arthritis through Shared CD8+ T Cell Clones
Source: Arthritis Rheumatol. 2025 Sep 21;78(1):152–65. doi: 10.1002/art.43286 (PMC12854012; doi:10.1002/art.43286)
Supplement: Supplementary file 3 — Supplementary Table 1: [file ART-78-152-s007.docx]

**Table S1** – Participant information, psoriasis/PsA associated HLA genotypes and single cell yield from each participant.

|  | **PsA 1** | **PsA 2** | **PsA 3** | **PsA 4** | **PsA 5** | **PsA 6** | **PsA 7** |
| --- | --- | --- | --- | --- | --- | --- | --- |
| **Demographic / clinical information** | | | | | | | |
| **Age (years)** | 55 | 37 | 30 | 59 | 48 | 49 | 35 |
| **Sex** | Female | Female | Male | Male | Male | Male | Female |
| **Psoriasis duration (years)** | ~20 | 5 | 15 | 34 | 32 | 5 | 29 |
| **PsA duration (years)** | 30 | <1 | ~15 | 11 | ~30 | <1 | 21 |
| **Current immunosuppressive medication** | Nil | Nil | Prednisolone 5mg | Bimekizumab (1 dose)  Sulfasalazine | Adalimumab (secondary failure) | Nil | Methotrexate |
| **PASI score** | 2.6 | 2.4 | 2.7 | 10.9 | 4.4 | 7.5 | 2/2 |
| **66/68 swollen/tender joint count** | 1/1 | 2/5 | 10/12 | 26/23 | 5/5 | 3/5 | 7 |
| **CRP** | NA | 11 | 19 | 39 | 21 | 9 | NA |
| **Samples for scRNAseq** | Skin, ST, SF, PB | Skin, ST, SF, PB | Skin, ST, SF, PB | Skin, ST, SF, PB | Skin, ST, PB | Skin, SF, PB | No |
| **Samples for spatial transcriptomics** | No | ST | ST | Skin | Skin & ST | No | ST |
| **HLA typing by next generation sequencing** | | | | | | | |
| **PsA/psoriasis associated HLA genotypes**^1,2^ | B*08:01-C*07:01  B*37:01-C*06:02 | Genotype unknown | B*08:01-C*07:01 | B*38:01-C*12:03 | B*57:01-C*06:02 | B*38:01-C*12:03 | Genotype unknown |
| **scRNAseq: Number memory T cells that passed QC** | | | | | | | |
| **Total, including cells which were not attributed to a tissue** | 7652 | 5627 | 6629 | 5707 | 2005 | 7871 | NA |
| **Skin epidermis** | 30 | 186 | 1196 | 772 | 176 | 2980 | NA |
| **Synovial tissue** | 2154 | 1037 | 502 | 813 | 647 | 0 | NA |
| **Synovial fluid** | 2349 | 2120 | 2258 | 2151 | 0 | 1296 | NA |
| **Blood** | 2899 | 2132 | 2495 | 1863 | 741 | 2687 | NA |
| **scRNAseq: Number of memory CD8+ T cells with paired RNA libraries and TCR sequences** | | | | | | | |
| **Skin epidermis** | 18 | 112 | 818 | 471 | 110 | 1555 | NA |
| **Synovial (tissue and fluid combined)** | 1463 | 802 | 1311 | 1586 | 199 | 388 | NA |
| **Blood** | 139 | 421 | 570 | 757 | 343 | 1002 | NA |
| **scRNAseq: Number of memory CD4+ T cells with paired RNA libraries and TCR sequences** | | | | | | | |
| **Skin epidermis** | 4 | 57 | 259 | 205 | 49 | 721 | NA |
| **Synovial (tissue and fluid combined)** | 2060 | 1953 | 1088 | 1034 | 385 | 718 | NA |
| **Blood** | 2023 | 1404 | 1603 | 868 | 270 | 1135 | NA |

1. Haroon, M., Winchester, R., Giles, J. T., *et al.* Certain class I HLA alleles and haplotypes implicated in susceptibility play a role in determining specific features of the psoriatic arthritis phenotype. *Ann Rheum Dis* 75, 155–162 (2016).

2. Winchester, R., Minevich, G., Steshenko, V., *et al.* HLA associations reveal genetic heterogeneity in psoriatic arthritis and in the psoriasis phenotype. *Arthritis Rheum* 64, 1134–1144 (2012).
