# Supplementary material for: Linking Skin and Joint Inflammation in Psoriatic Arthritis through Shared CD8+ T Cell Clones
Source: Arthritis Rheumatol. 2025 Sep 21;78(1):152–65. doi: 10.1002/art.43286 (PMC12854012; doi:10.1002/art.43286)
Supplement: Supplementary file 4 — Supplementary Table 2: [file ART-78-152-s005.docx]

**Table S2: Hashtags used to stain each patient sample**

Biolegend TotalSeq-C hashtags (anti-human, clone LNH-94 & 2M2, isotype: mouse IgG1)

| **Patient** | **Hashtag** | | | | | | |
| --- | --- | --- | --- | --- | --- | --- | --- |
|  | **Blood** | | **SF** | | **ST** | | **Skin** |
| PsA 1 | 1 | 2 | 3 | 4 | 6 | | 5 |
| PsA 2 | 1 | 2 | 3 | 4 | 6 | | 5 |
| PsA 3 | 1 | 2 | 3 | 4 | 6 | | 5 |
| PsA 4 | 1 | 2 | 3 | | 4 | 5 | 6 |
| PsA 5 | 1 | 2 | NA | | 3 | 4 | 5 |
| PsA 6 | 1 | 2 | 3 | | 6 | | 5 |
