# Supplementary material for: Linking Skin and Joint Inflammation in Psoriatic Arthritis through Shared CD8+ T Cell Clones
Source: Arthritis Rheumatol. 2025 Sep 21;78(1):152–65. doi: 10.1002/art.43286 (PMC12854012; doi:10.1002/art.43286)
Supplement: Supplementary file 5 — Supplementary Table 3: [file ART-78-152-s004.docx]

**Table S3: Antibodies used for Fluorescence Activated Cell Sorting and CITE-seq**

| **Fluorescence – conjugated antibodies** | | |
| --- | --- | --- |
| **Antibody** | **Clone** | **Manufacturer** |
| CD14 – APCcy7 | REA599 | Miltenyi |
| CD3 – PEcy7 | UCHT1 | Biolegend |
| CD45 – BUV395 | HI30 | Becton Dickinson UK Ltd |
| CD27 – APC | O323 | Biolegend |
| CD45RA – BV711 | HI100 | Biolegend |
| **TotalSeqC antibodies** | | |
| **Antibody** | **Clone** | **Manufacturer** |
| PD1 | EH12.2H7 | Biolegend |
| CCR6 | G034E3 | Biolegend |
| CD103 | Ber-ACT8 | Biolegend |
| CD69 | FN50 | Biolegend |
| CD161 | HP-3G10 | Biolegend |
| CD49a | TS2/7 | Biolegend |
| CXCR6 | K041E5 | Biolegend |
| CD8A | RPA-T8 | Biolegend |
| Anti-human Hastags | LNH-94; 2M2 | Biolegend |
