# Supplementary material for: Linking Skin and Joint Inflammation in Psoriatic Arthritis through Shared CD8+ T Cell Clones
Source: Arthritis Rheumatol. 2025 Sep 21;78(1):152–65. doi: 10.1002/art.43286 (PMC12854012; doi:10.1002/art.43286)
Supplement: Supplementary file 12 — Supplementary Data 6: [file ART-78-152-s009.pdf]

ICMJE DISCLOSURE FORM

Date: 1 February 2025
Your Name: Lucy E Durham
Manuscript Title: Clonal sharing of CD8+ T-cells links skin and joint inflammation in psoriatic arthritis
Manuscript number (if known): ar-24-1539

In the interest of transparency, we ask you to disclose all relationships/activities/interests listed below that are related to the content of your manuscript. "Related" means any relation with for-profit or not-for-profit third parties whose interests may be affected by the content of the manuscript. Disclosure represents a commitment to transparency and does not necessarily indicate a bias. If you are in doubt about whether to list a relationship/activity/interest, it is preferable that you do so.

The following questions apply to the author's relationships/activities/interests as they relate to the current manuscript only.

The author's relationships/activities/interests should be defined broadly. For example, if your manuscript pertains to the epidemiology of hypertension, you should declare all relationships with manufacturers of antihypertensive medication, even if that medication is not mentioned in the manuscript.

In item #1 below, report all support for the work reported in this manuscript without time limit. For all other items, the time frame for disclosure is the past 36 months.

		**Name all entities with whom you have this relationship or indicate none (add rows as needed)**	**Specifications/Comments (e.g., if payments were made to you or to your institution)**
		**Time frame: Since the initial planning of the work**	
1	All support for the present manuscript (e.g., funding, provision of study materials, medical writing, article processing charges, etc.) **No time limit for this item.**	MRC	To King's College London
		**Time frame: past 36 months**	
2	Grants or contracts from any entity (if not indicated in item #1 above).	AstraZeneca-MRC Industry partnership	To Kings College London<br>I have received research funding from an AstraZeneca-MRC Industry partnership grant outside of this work
3	Royalties or licenses	X	
4	Consulting fees	X	

5	Payment or honoraria for lectures, presentations, speakers bureaus, manuscript writing or educational events	X	
6	Payment for expert testimony	X	
7	Support for attending meetings and/or travel	X	
8	Patents planned, issued or pending	X	
9	Participation on a Data Safety Monitoring Board or Advisory Board	X	
10	Leadership or fiduciary role in other board, society, committee or advocacy group, paid or unpaid	X	
11	Stock or stock options	X	
12	Receipt of equipment, materials, drugs, medical writing, gifts or other services	X	
13	Other financial or non-financial interests	After completion of this work and submission of the manuscript I became an employee at AstraZeneca.	

Please place an "X" next to the following statement to indicate your agreement:

X I certify that I have answered every question and have not altered the wording of any of the questions on this form.

ICMJE DISCLOSURE FORM

Date: 1 February 2025
Your Name: Frances Humby
Manuscript Title: Clonal sharing of CD8+ T-cells links skin and joint inflammation in psoriatic arthritis
Manuscript number (if known): ar-24-1539

In the interest of transparency, we ask you to disclose all relationships/activities/interests listed below that are related to the content of your manuscript. "Related" means any relation with for-profit or not-for-profit third parties whose interests may be affected by the content of the manuscript. Disclosure represents a commitment to transparency and does not necessarily indicate a bias. If you are in doubt about whether to list a relationship/activity/interest, it is preferable that you do so.

The following questions apply to the author's relationships/activities/interests as they relate to the current manuscript only.

The author's relationships/activities/interests should be defined broadly. For example, if your manuscript pertains to the epidemiology of hypertension, you should declare all relationships with manufacturers of antihypertensive medication, even if that medication is not mentioned in the manuscript.

In item #1 below, report all support for the work reported in this manuscript without time limit. For all other items, the time frame for disclosure is the past 36 months.

		**Name all entities with whom you have this relationship or indicate none (add rows as needed)**	**Specifications/Comments (e.g., if payments were made to you or to your institution)**
**Time frame: Since the initial planning of the work**			
1	All support for the present manuscript (e.g., funding, provision of study materials, medical writing, article processing charges, etc.) **No time limit for this item.**	X	
**Time frame: past 36 months**			
2	Grants or contracts from any entity (if not indicated in item #1 above).	X	
3	Royalties or licenses	X	
4	Consulting fees	X	

5	Payment or honoraria for lectures, presentations, speakers bureaus, manuscript writing or educational events	X	
6	Payment for expert testimony	X	
7	Support for attending meetings and/or travel	X	
8	Patents planned, issued or pending	X	
9	Participation on a Data Safety Monitoring Board or Advisory Board	X	
10	Leadership or fiduciary role in other board, society, committee or advocacy group, paid or unpaid	X	
11	Stock or stock options	X	
12	Receipt of equipment, materials, drugs, medical writing, gifts or other services	X	
13	Other financial or non-financial interests	X	

Please place an "X" next to the following statement to indicate your agreement:

X I certify that I have answered every question and have not altered the wording of any of the questions on this form.

ICMJE DISCLOSURE FORM

Date: 1 February 2025
Your Name: Nora Ng
Manuscript Title: Clonal sharing of CD8+ T-cells links skin and joint inflammation in psoriatic arthritis
Manuscript number (if known): ar-24-1539

In the interest of transparency, we ask you to disclose all relationships/activities/interests listed below that are related to the content of your manuscript. "Related" means any relation with for-profit or not-for-profit third parties whose interests may be affected by the content of the manuscript. Disclosure represents a commitment to transparency and does not necessarily indicate a bias. If you are in doubt about whether to list a relationship/activity/interest, it is preferable that you do so.

The following questions apply to the author's relationships/activities/interests as they relate to the current manuscript only.

The author's relationships/activities/interests should be defined broadly. For example, if your manuscript pertains to the epidemiology of hypertension, you should declare all relationships with manufacturers of antihypertensive medication, even if that medication is not mentioned in the manuscript.

In item #1 below, report all support for the work reported in this manuscript without time limit. For all other items, the time frame for disclosure is the past 36 months.

		**Name all entities with whom you have this relationship or indicate none (add rows as needed)**	**Specifications/Comments (e.g., if payments were made to you or to your institution)**
**Time frame: Since the initial planning of the work**			
1	All support for the present manuscript (e.g., funding, provision of study materials, medical writing, article processing charges, etc.) **No time limit for this item.**	X	
**Time frame: past 36 months**			
2	Grants or contracts from any entity (if not indicated in item #1 above).	X	
3	Royalties or licenses	X	
4	Consulting fees	X	

5	Payment or honoraria for lectures, presentations, speakers bureaus, manuscript writing or educational events	X	
6	Payment for expert testimony	X	
7	Support for attending meetings and/or travel	X	
8	Patents planned, issued or pending	X	
9	Participation on a Data Safety Monitoring Board or Advisory Board	X	
10	Leadership or fiduciary role in other board, society, committee or advocacy group, paid or unpaid	X	
11	Stock or stock options	X	
12	Receipt of equipment, materials, drugs, medical writing, gifts or other services	X	
13	Other financial or non-financial interests	X	

Please place an "X" next to the following statement to indicate your agreement:

X I certify that I have answered every question and have not altered the wording of any of the questions on this form.

ICMJE DISCLOSURE FORM

Date: 1 February 2025
Your Name: Roman Laddach
Manuscript Title: Clonal sharing of CD8+ T-cells links skin and joint inflammation in psoriatic arthritis
Manuscript number (if known): ar-24-1539

In the interest of transparency, we ask you to disclose all relationships/activities/interests listed below that are related to the content of your manuscript. "Related" means any relation with for-profit or not-for-profit third parties whose interests may be affected by the content of the manuscript. Disclosure represents a commitment to transparency and does not necessarily indicate a bias. If you are in doubt about whether to list a relationship/activity/interest, it is preferable that you do so.

The following questions apply to the author's relationships/activities/interests as they relate to the current manuscript only.

The author's relationships/activities/interests should be defined broadly. For example, if your manuscript pertains to the epidemiology of hypertension, you should declare all relationships with manufacturers of antihypertensive medication, even if that medication is not mentioned in the manuscript.

In item #1 below, report all support for the work reported in this manuscript without time limit. For all other items, the time frame for disclosure is the past 36 months.

		**Name all entities with whom you have this relationship or indicate none (add rows as needed)**	**Specifications/Comments (e.g., if payments were made to you or to your institution)**
		**Time frame: Since the initial planning of the work**	
1	All support for the present manuscript (e.g., funding, provision of study materials, medical writing, article processing charges, etc.) **No time limit for this item.**	X	
		**Time frame: past 36 months**	
2	Grants or contracts from any entity (if not indicated in item #1 above).	X	
3	Royalties or licenses	X	
4	Consulting fees	X	

5	Payment or honoraria for lectures, presentations, speakers bureaus, manuscript writing or educational events	X	
6	Payment for expert testimony	X	
7	Support for attending meetings and/or travel	X	
8	Patents planned, issued or pending	X	
9	Participation on a Data Safety Monitoring Board or Advisory Board	X	
10	Leadership or fiduciary role in other board, society, committee or advocacy group, paid or unpaid	X	
11	Stock or stock options	X	
12	Receipt of equipment, materials, drugs, medical writing, gifts or other services	X	
13	Other financial or non-financial interests	X	

Please place an "X" next to the following statement to indicate your agreement:

X I certify that I have answered every question and have not altered the wording of any of the questions on this form.

ICMJE DISCLOSURE FORM

Date: 1 February 2025
Your Name: Elizabeth H Gray
Manuscript Title: Clonal sharing of CD8+ T-cells links skin and joint inflammation in psoriatic arthritis
Manuscript number (if known): ar-24-1539

In the interest of transparency, we ask you to disclose all relationships/activities/interests listed below that are related to the content of your manuscript. "Related" means any relation with for-profit or not-for-profit third parties whose interests may be affected by the content of the manuscript. Disclosure represents a commitment to transparency and does not necessarily indicate a bias. If you are in doubt about whether to list a relationship/activity/interest, it is preferable that you do so.

The following questions apply to the author's relationships/activities/interests as they relate to the current manuscript only.

The author's relationships/activities/interests should be defined broadly. For example, if your manuscript pertains to the epidemiology of hypertension, you should declare all relationships with manufacturers of antihypertensive medication, even if that medication is not mentioned in the manuscript.

In item #1 below, report all support for the work reported in this manuscript without time limit. For all other items, the time frame for disclosure is the past 36 months.

		**Name all entities with whom you have this relationship or indicate none (add rows as needed)**	**Specifications/Comments (e.g., if payments were made to you or to your institution)**
**Time frame: Since the initial planning of the work**			
1	All support for the present manuscript (e.g., funding, provision of study materials, medical writing, article processing charges, etc.) **No time limit for this item.**	X	
**Time frame: past 36 months**			
2	Grants or contracts from any entity (if not indicated in item #1 above).	X	
3	Royalties or licenses	X	
4	Consulting fees	X	

5	Payment or honoraria for lectures, presentations, speakers bureaus, manuscript writing or educational events	X	
6	Payment for expert testimony	X	
7	Support for attending meetings and/or travel	X	
8	Patents planned, issued or pending	X	
9	Participation on a Data Safety Monitoring Board or Advisory Board	X	
10	Leadership or fiduciary role in other board, society, committee or advocacy group, paid or unpaid	X	
11	Stock or stock options	X	
12	Receipt of equipment, materials, drugs, medical writing, gifts or other services	X	
13	Other financial or non-financial interests	X	

Please place an "X" next to the following statement to indicate your agreement:

X I certify that I have answered every question and have not altered the wording of any of the questions on this form.

ICMJE DISCLOSURE FORM

Date: 1 February 2025
Your Name: Sarah E Ryan
Manuscript Title: Clonal sharing of CD8+ T-cells links skin and joint inflammation in psoriatic arthritis
Manuscript number (if known): ar-24-1539

In the interest of transparency, we ask you to disclose all relationships/activities/interests listed below that are related to the content of your manuscript. "Related" means any relation with for-profit or not-for-profit third parties whose interests may be affected by the content of the manuscript. Disclosure represents a commitment to transparency and does not necessarily indicate a bias. If you are in doubt about whether to list a relationship/activity/interest, it is preferable that you do so.

The following questions apply to the author's relationships/activities/interests as they relate to the current manuscript only.

The author's relationships/activities/interests should be defined broadly. For example, if your manuscript pertains to the epidemiology of hypertension, you should declare all relationships with manufacturers of antihypertensive medication, even if that medication is not mentioned in the manuscript.

In item #1 below, report all support for the work reported in this manuscript without time limit. For all other items, the time frame for disclosure is the past 36 months.

		**Name all entities with whom you have this relationship or indicate none (add rows as needed)**	**Specifications/Comments (e.g., if payments were made to you or to your institution)**
**Time frame: Since the initial planning of the work**			
1	All support for the present manuscript (e.g., funding, provision of study materials, medical writing, article processing charges, etc.) **No time limit for this item.**	X	
**Time frame: past 36 months**			
2	Grants or contracts from any entity (if not indicated in item #1 above).	X	
3	Royalties or licenses	X	
4	Consulting fees	X	

5	Payment or honoraria for lectures, presentations, speakers bureaus, manuscript writing or educational events	X	
6	Payment for expert testimony	X	
7	Support for attending meetings and/or travel	X	
8	Patents planned, issued or pending	X	
9	Participation on a Data Safety Monitoring Board or Advisory Board	X	
10	Leadership or fiduciary role in other board, society, committee or advocacy group, paid or unpaid	X	
11	Stock or stock options	X	
12	Receipt of equipment, materials, drugs, medical writing, gifts or other services	X	
13	Other financial or non-financial interests	X	

Please place an "X" next to the following statement to indicate your agreement:

X I certify that I have answered every question and have not altered the wording of any of the questions on this form.

ICMJE DISCLOSURE FORM

Date: 1 February 2025
Your Name: Kathryn J.A. Steel
Manuscript Title: Clonal sharing of CD8+ T-cells links skin and joint inflammation in psoriatic arthritis
Manuscript number (if known): ar-24-1539

In the interest of transparency, we ask you to disclose all relationships/activities/interests listed below that are related to the content of your manuscript. "Related" means any relation with for-profit or not-for-profit third parties whose interests may be affected by the content of the manuscript. Disclosure represents a commitment to transparency and does not necessarily indicate a bias. If you are in doubt about whether to list a relationship/activity/interest, it is preferable that you do so.

The following questions apply to the author's relationships/activities/interests as they relate to the current manuscript only.

The author's relationships/activities/interests should be defined broadly. For example, if your manuscript pertains to the epidemiology of hypertension, you should declare all relationships with manufacturers of antihypertensive medication, even if that medication is not mentioned in the manuscript.

In item #1 below, report all support for the work reported in this manuscript without time limit. For all other items, the time frame for disclosure is the past 36 months.

		**Name all entities with whom you have this relationship or indicate none (add rows as needed)**	**Specifications/Comments (e.g., if payments were made to you or to your institution)**
		**Time frame: Since the initial planning of the work**	
1	All support for the present manuscript (e.g., funding, provision of study materials, medical writing, article processing charges, etc.) **No time limit for this item.**	X	
		**Time frame: past 36 months**	
2	Grants or contracts from any entity (if not indicated in item #1 above).	X	
3	Royalties or licenses	X	
4	Consulting fees	X	

5	Payment or honoraria for lectures, presentations, speakers bureaus, manuscript writing or educational events	X	
6	Payment for expert testimony	X	
7	Support for attending meetings and/or travel	X	
8	Patents planned, issued or pending	X	
9	Participation on a Data Safety Monitoring Board or Advisory Board	X	
10	Leadership or fiduciary role in other board, society, committee or advocacy group, paid or unpaid	X	
11	Stock or stock options	X	
12	Receipt of equipment, materials, drugs, medical writing, gifts or other services	X	
13	Other financial or non-financial interests	X	

Please place an "X" next to the following statement to indicate your agreement:

X I certify that I have answered every question and have not altered the wording of any of the questions on this form.

ICMJE DISCLOSURE FORM

Date: 1 February 2025
Your Name: Rosie Ross
Manuscript Title: Clonal sharing of CD8+ T-cells links skin and joint inflammation in psoriatic arthritis
Manuscript number (if known): ar-24-1539

In the interest of transparency, we ask you to disclose all relationships/activities/interests listed below that are related to the content of your manuscript. "Related" means any relation with for-profit or not-for-profit third parties whose interests may be affected by the content of the manuscript. Disclosure represents a commitment to transparency and does not necessarily indicate a bias. If you are in doubt about whether to list a relationship/activity/interest, it is preferable that you do so.

The following questions apply to the author's relationships/activities/interests as they relate to the current manuscript only.

The author's relationships/activities/interests should be defined broadly. For example, if your manuscript pertains to the epidemiology of hypertension, you should declare all relationships with manufacturers of antihypertensive medication, even if that medication is not mentioned in the manuscript.

In item #1 below, report all support for the work reported in this manuscript without time limit. For all other items, the time frame for disclosure is the past 36 months.

		**Name all entities with whom you have this relationship or indicate none (add rows as needed)**	**Specifications/Comments (e.g., if payments were made to you or to your institution)**
		**Time frame: Since the initial planning of the work**	
1	All support for the present manuscript (e.g., funding, provision of study materials, medical writing, article processing charges, etc.) **No time limit for this item.**	X	
		**Time frame: past 36 months**	
2	Grants or contracts from any entity (if not indicated in item #1 above).	X	
3	Royalties or licenses	X	
4	Consulting fees	X	

5	Payment or honoraria for lectures, presentations, speakers bureaus, manuscript writing or educational events	X	
6	Payment for expert testimony	X	
7	Support for attending meetings and/or travel	X	
8	Patents planned, issued or pending	X	
9	Participation on a Data Safety Monitoring Board or Advisory Board	X	
10	Leadership or fiduciary role in other board, society, committee or advocacy group, paid or unpaid	X	
11	Stock or stock options	X	
12	Receipt of equipment, materials, drugs, medical writing, gifts or other services	X	
13	Other financial or non-financial interests	X	

Please place an "X" next to the following statement to indicate your agreement:

X I certify that I have answered every question and have not altered the wording of any of the questions on this form.

ICMJE DISCLOSURE FORM

Date: 1 February 2025
Your Name: Giovanni Povoleri
Manuscript Title: Clonal sharing of CD8+ T-cells links skin and joint inflammation in psoriatic arthritis
Manuscript number (if known): ar-24-1539

In the interest of transparency, we ask you to disclose all relationships/activities/interests listed below that are related to the content of your manuscript. "Related" means any relation with for-profit or not-for-profit third parties whose interests may be affected by the content of the manuscript. Disclosure represents a commitment to transparency and does not necessarily indicate a bias. If you are in doubt about whether to list a relationship/activity/interest, it is preferable that you do so.

The following questions apply to the author's relationships/activities/interests as they relate to the current manuscript only.

The author's relationships/activities/interests should be defined broadly. For example, if your manuscript pertains to the epidemiology of hypertension, you should declare all relationships with manufacturers of antihypertensive medication, even if that medication is not mentioned in the manuscript.

In item #1 below, report all support for the work reported in this manuscript without time limit. For all other items, the time frame for disclosure is the past 36 months.

		**Name all entities with whom you have this relationship or indicate none (add rows as needed)**	**Specifications/Comments (e.g., if payments were made to you or to your institution)**
**Time frame: Since the initial planning of the work**			
1	All support for the present manuscript (e.g., funding, provision of study materials, medical writing, article processing charges, etc.) **No time limit for this item.**	X	
**Time frame: past 36 months**			
2	Grants or contracts from any entity (if not indicated in item #1 above).	X	
3	Royalties or licenses	X	
4	Consulting fees	X	

5	Payment or honoraria for lectures, presentations, speakers bureaus, manuscript writing or educational events	X	
6	Payment for expert testimony	X	
7	Support for attending meetings and/or travel	X	
8	Patents planned, issued or pending	X	
9	Participation on a Data Safety Monitoring Board or Advisory Board	X	
10	Leadership or fiduciary role in other board, society, committee or advocacy group, paid or unpaid	X	
11	Stock or stock options	X	
12	Receipt of equipment, materials, drugs, medical writing, gifts or other services	X	
13	Other financial or non-financial interests	X	

Please place an "X" next to the following statement to indicate your agreement:

X I certify that I have answered every question and have not altered the wording of any of the questions on this form.

ICMJE DISCLOSURE FORM

Date: 1 February 2025
Your Name: Rosamund Nuamah
Manuscript Title: Clonal sharing of CD8+ T-cells links skin and joint inflammation in psoriatic arthritis
Manuscript number (if known): ar-24-1539

In the interest of transparency, we ask you to disclose all relationships/activities/interests listed below that are related to the content of your manuscript. "Related" means any relation with for-profit or not-for-profit third parties whose interests may be affected by the content of the manuscript. Disclosure represents a commitment to transparency and does not necessarily indicate a bias. If you are in doubt about whether to list a relationship/activity/interest, it is preferable that you do so.

The following questions apply to the author's relationships/activities/interests as they relate to the current manuscript only.

The author's relationships/activities/interests should be defined broadly. For example, if your manuscript pertains to the epidemiology of hypertension, you should declare all relationships with manufacturers of antihypertensive medication, even if that medication is not mentioned in the manuscript.

In item #1 below, report all support for the work reported in this manuscript without time limit. For all other items, the time frame for disclosure is the past 36 months.

		**Name all entities with whom you have this relationship or indicate none (add rows as needed)**	**Specifications/Comments (e.g., if payments were made to you or to your institution)**
		**Time frame: Since the initial planning of the work**	
1	All support for the present manuscript (e.g., funding, provision of study materials, medical writing, article processing charges, etc.) **No time limit for this item.**	X	
		**Time frame: past 36 months**	
2	Grants or contracts from any entity (if not indicated in item #1 above).	X	
3	Royalties or licenses	X	
4	Consulting fees	X	

5	Payment or honoraria for lectures, presentations, speakers bureaus, manuscript writing or educational events	X	
6	Payment for expert testimony	X	
7	Support for attending meetings and/or travel	X	
8	Patents planned, issued or pending	X	
9	Participation on a Data Safety Monitoring Board or Advisory Board	X	
10	Leadership or fiduciary role in other board, society, committee or advocacy group, paid or unpaid	X	
11	Stock or stock options	X	
12	Receipt of equipment, materials, drugs, medical writing, gifts or other services	X	
13	Other financial or non-financial interests	X	

Please place an "X" next to the following statement to indicate your agreement:

X I certify that I have answered every question and have not altered the wording of any of the questions on this form.

ICMJE DISCLOSURE FORM

Date: 1 February 2025
Your Name: Kathy Fung
Manuscript Title: Clonal sharing of CD8+ T-cells links skin and joint inflammation in psoriatic arthritis
Manuscript number (if known): ar-24-1539

In the interest of transparency, we ask you to disclose all relationships/activities/interests listed below that are related to the content of your manuscript. "Related" means any relation with for-profit or not-for-profit third parties whose interests may be affected by the content of the manuscript. Disclosure represents a commitment to transparency and does not necessarily indicate a bias. If you are in doubt about whether to list a relationship/activity/interest, it is preferable that you do so.

The following questions apply to the author's relationships/activities/interests as they relate to the current manuscript only.

The author's relationships/activities/interests should be defined broadly. For example, if your manuscript pertains to the epidemiology of hypertension, you should declare all relationships with manufacturers of antihypertensive medication, even if that medication is not mentioned in the manuscript.

In item #1 below, report all support for the work reported in this manuscript without time limit. For all other items, the time frame for disclosure is the past 36 months.

		**Name all entities with whom you have this relationship or indicate none (add rows as needed)**	**Specifications/Comments (e.g., if payments were made to you or to your institution)**
		**Time frame: Since the initial planning of the work**	
1	All support for the present manuscript (e.g., funding, provision of study materials, medical writing, article processing charges, etc.) **No time limit for this item.**	X	
		**Time frame: past 36 months**	
2	Grants or contracts from any entity (if not indicated in item #1 above).	X	
3	Royalties or licenses	X	
4	Consulting fees	X	

5	Payment or honoraria for lectures, presentations, speakers bureaus, manuscript writing or educational events	X	
6	Payment for expert testimony	X	
7	Support for attending meetings and/or travel	X	
8	Patents planned, issued or pending	X	
9	Participation on a Data Safety Monitoring Board or Advisory Board	X	
10	Leadership or fiduciary role in other board, society, committee or advocacy group, paid or unpaid	X	
11	Stock or stock options	X	
12	Receipt of equipment, materials, drugs, medical writing, gifts or other services	X	
13	Other financial or non-financial interests	X	

Please place an "X" next to the following statement to indicate your agreement:

X I certify that I have answered every question and have not altered the wording of any of the questions on this form.

ICMJE DISCLOSURE FORM

Date: 1 February 2025
Your Name: Athul Menon Kallayil
Manuscript Title: Clonal sharing of CD8+ T-cells links skin and joint inflammation in psoriatic arthritis
Manuscript number (if known): ar-24-1539

In the interest of transparency, we ask you to disclose all relationships/activities/interests listed below that are related to the content of your manuscript. "Related" means any relation with for-profit or not-for-profit third parties whose interests may be affected by the content of the manuscript. Disclosure represents a commitment to transparency and does not necessarily indicate a bias. If you are in doubt about whether to list a relationship/activity/interest, it is preferable that you do so.

The following questions apply to the author's relationships/activities/interests as they relate to the current manuscript only.

The author's relationships/activities/interests should be defined broadly. For example, if your manuscript pertains to the epidemiology of hypertension, you should declare all relationships with manufacturers of antihypertensive medication, even if that medication is not mentioned in the manuscript.

In item #1 below, report all support for the work reported in this manuscript without time limit. For all other items, the time frame for disclosure is the past 36 months.

		**Name all entities with whom you have this relationship or indicate none (add rows as needed)**	**Specifications/Comments (e.g., if payments were made to you or to your institution)**
		**Time frame: Since the initial planning of the work**	
1	All support for the present manuscript (e.g., funding, provision of study materials, medical writing, article processing charges, etc.) **No time limit for this item.**	X	
		**Time frame: past 36 months**	
2	Grants or contracts from any entity (if not indicated in item #1 above).	X	
3	Royalties or licenses	X	
4	Consulting fees	X	

5	Payment or honoraria for lectures, presentations, speakers bureaus, manuscript writing or educational events	X	
6	Payment for expert testimony	X	
7	Support for attending meetings and/or travel	X	
8	Patents planned, issued or pending	X	
9	Participation on a Data Safety Monitoring Board or Advisory Board	X	
10	Leadership or fiduciary role in other board, society, committee or advocacy group, paid or unpaid	X	
11	Stock or stock options	X	
12	Receipt of equipment, materials, drugs, medical writing, gifts or other services	X	
13	Other financial or non-financial interests	X	

Please place an "X" next to the following statement to indicate your agreement:

X I certify that I have answered every question and have not altered the wording of any of the questions on this form.

ICMJE DISCLOSURE FORM

Date: 1 February 2025
Your Name: Pawan Dhami
Manuscript Title: Clonal sharing of CD8+ T-cells links skin and joint inflammation in psoriatic arthritis
Manuscript number (if known): ar-24-1539

In the interest of transparency, we ask you to disclose all relationships/activities/interests listed below that are related to the content of your manuscript. "Related" means any relation with for-profit or not-for-profit third parties whose interests may be affected by the content of the manuscript. Disclosure represents a commitment to transparency and does not necessarily indicate a bias. If you are in doubt about whether to list a relationship/activity/interest, it is preferable that you do so.

The following questions apply to the author's relationships/activities/interests as they relate to the current manuscript only.

The author's relationships/activities/interests should be defined broadly. For example, if your manuscript pertains to the epidemiology of hypertension, you should declare all relationships with manufacturers of antihypertensive medication, even if that medication is not mentioned in the manuscript.

In item #1 below, report all support for the work reported in this manuscript without time limit. For all other items, the time frame for disclosure is the past 36 months.

		**Name all entities with whom you have this relationship or indicate none (add rows as needed)**	**Specifications/Comments (e.g., if payments were made to you or to your institution)**
		**Time frame: Since the initial planning of the work**	
1	All support for the present manuscript (e.g., funding, provision of study materials, medical writing, article processing charges, etc.) **No time limit for this item.**	X	
		**Time frame: past 36 months**	
2	Grants or contracts from any entity (if not indicated in item #1 above).	X	
3	Royalties or licenses	X	
4	Consulting fees	X	

5	Payment or honoraria for lectures, presentations, speakers bureaus, manuscript writing or educational events	X	
6	Payment for expert testimony	X	
7	Support for attending meetings and/or travel	X	
8	Patents planned, issued or pending	X	
9	Participation on a Data Safety Monitoring Board or Advisory Board	X	
10	Leadership or fiduciary role in other board, society, committee or advocacy group, paid or unpaid	X	
11	Stock or stock options	X	
12	Receipt of equipment, materials, drugs, medical writing, gifts or other services	X	
13	Other financial or non-financial interests	X	

Please place an "X" next to the following statement to indicate your agreement:

X I certify that I have answered every question and have not altered the wording of any of the questions on this form.

ICMJE DISCLOSURE FORM

Date: 1 February 2025
Your Name: Bruce W. Kirkham
Manuscript Title: Clonal sharing of CD8+ T-cells links skin and joint inflammation in psoriatic arthritis
Manuscript number (if known): ar-24-1539

In the interest of transparency, we ask you to disclose all relationships/activities/interests listed below that are related to the content of your manuscript. "Related" means any relation with for-profit or not-for-profit third parties whose interests may be affected by the content of the manuscript. Disclosure represents a commitment to transparency and does not necessarily indicate a bias. If you are in doubt about whether to list a relationship/activity/interest, it is preferable that you do so.

The following questions apply to the author's relationships/activities/interests as they relate to the current manuscript only.

The author's relationships/activities/interests should be defined broadly. For example, if your manuscript pertains to the epidemiology of hypertension, you should declare all relationships with manufacturers of antihypertensive medication, even if that medication is not mentioned in the manuscript.

In item #1 below, report all support for the work reported in this manuscript without time limit. For all other items, the time frame for disclosure is the past 36 months.

		**Name all entities with whom you have this relationship or indicate none (add rows as needed)**	**Specifications/Comments (e.g., if payments were made to you or to your institution)**
**Time frame: Since the initial planning of the work**			
1	All support for the present manuscript (e.g., funding, provision of study materials, medical writing, article processing charges, etc.) **No time limit for this item.**	X	
**Time frame: past 36 months**			
2	Grants or contracts from any entity (if not indicated in item #1 above).	X	
3	Royalties or licenses	X	
4	Consulting fees	X	
		Eli Lilly, Novartis, Pfizer and UCB	

5	Payment or honoraria for lectures, presentations, speakers bureaus, manuscript writing or educational events	X	
		AbbVie, Eli Lilly, Galapagos, Janssen, Novartis, Pfizer and UCB	
6	Payment for expert testimony	X	
7	Support for attending meetings and/or travel	X	
		Eli Lilly, Novartis, and UCB	
8	Patents planned, issued or pending	X	
9	Participation on a Data Safety Monitoring Board or Advisory Board	X	
10	Leadership or fiduciary role in other board, society, committee or advocacy group, paid or unpaid	X	
11	Stock or stock options	X	
12	Receipt of equipment, materials, drugs, medical writing, gifts or other services	X	
13	Other financial or non-financial interests	X	

Please place an "X" next to the following statement to indicate your agreement:

X I certify that I have answered every question and have not altered the wording of any of the questions on this form.

ICMJE DISCLOSURE FORM

Date: 27 January 2025
Your Name: Leonie S. Taams
Manuscript Title: Clonal sharing of CD8+ T-cells links skin and joint inflammation in psoriatic arthritis
Manuscript number (if known): ar-24-1539

In the interest of transparency, we ask you to disclose all relationships/activities/interests listed below that are related to the content of your manuscript. "Related" means any relation with for-profit or not-for-profit third parties whose interests may be affected by the content of the manuscript. Disclosure represents a commitment to transparency and does not necessarily indicate a bias. If you are in doubt about whether to list a relationship/activity/interest, it is preferable that you do so.

The following questions apply to the author's relationships/activities/interests as they relate to the current manuscript only.

The author's relationships/activities/interests should be defined broadly. For example, if your manuscript pertains to the epidemiology of hypertension, you should declare all relationships with manufacturers of antihypertensive medication, even if that medication is not mentioned in the manuscript.

In item #1 below, report all support for the work reported in this manuscript without time limit. For all other items, the time frame for disclosure is the past 36 months.

		**Name all entities with whom you have this relationship or indicate none (add rows as needed)**	**Specifications/Comments (e.g., if payments were made to you or to your institution)**
		**Time frame: Since the initial planning of the work**	
1	All support for the present manuscript (e.g., funding, provision of study materials, medical writing, article processing charges, etc.) **No time limit for this item.**	MRC	To King's College London
		Versus Arthritis	To King's College London
		FOREUM	To King's College London
		King's Health Partners Centre for Translational Medicine	To King's College London and Guy's & St Thomas' Hospital
		CRUK City of London Award	To King's College London
		NIHR BRC	To King's College London and Guy's & St Thomas' Hospital
		**Time frame: past 36 months**	
2	Grants or contracts from any entity (if not indicated in item #1 above).	Wellcome Trust	To King's College London
		NIHR, BHF	To King's College London
		SNSF & NPF	To King's College London
3	Royalties or licenses	X	

4	Consulting fees	AbbVie	To King's College London & Leonie Taams
		IMID forum	To King's College London & Leonie Taams
		UCB	To King's College London & Leonie Taams
5	Payment or honoraria for lectures, presentations, speakers bureaus, manuscript writing or educational events	GSK	To King's College London & Leonie Taams
6	Payment for expert testimony	X	
7	Support for attending meetings and/or travel	FOCIS	Support for annual conference attendance to Leonie Taams
8	Patents planned, issued or pending	X	
9	Participation on a Data Safety Monitoring Board or Advisory Board	X	
10	Leadership or fiduciary role in other board, society, committee or advocacy group, paid or unpaid	Editor-in-Chief for Clin Exp Immunology, British Society for Immunology	To King's College London & Leonie Taams
		At large Director FOCIS	Unpaid
11	Stock or stock options	X	
12	Receipt of equipment, materials, drugs, medical writing, gifts or other services	X	
13	Other financial or non-financial interests	X	

Please place an "X" next to the following statement to indicate your agreement:

__X_ I certify that I have answered every question and have not altered the wording of any of the questions on this form.
